# Molecularly Engineered Silver(I) Gel Catalysts for Industrial-Level CO_2_ Electroreduction

**DOI:** 10.1021/acs.inorgchem.6c01721

**Published:** 2026-06-26

**Authors:** Simon Offenthaler, Sankitkumar Vala, Wolfgang Schöfberger

**Affiliations:** Institute of Organic Chemistry, Laboratory for Sustainable Chemistry and Catalysis (LSusCat), Johannes Kepler University (JKU), Altenberger Straße 69, 4040 Linz, Austria

## Abstract

Power-to-X strategies are a key approach for coupling renewable energy generation with storage and utilization pathways. Because renewable sources such as solar and wind are intermittent, surplus electricity must be converted into chemical energy carriers, including hydrogen, fuels, and chemical feedstocks. In this context, the capture and electrochemical conversion of CO_2_ into valuable products is particularly attractive, as it supports a circular carbon economy and mitigates greenhouse gas emissions. Herein, we report the solvochemical and mechanochemical synthesis and implementation of a triazine-based ligand system, 2,4,6-tri-(1*H*-pyrazol-1-yl)-1,3,5-triazine (**TPT-1**), and its silver­(I) complexes for electrochemical CO_2_ reduction. TPT-1 was synthesized via heteroaryl nucleophilic substitution of chlorine on a 1,3,5-triazine ring by pyrazolate. Subsequent metalation yielded the silver complexes **Ag­(TPT-1)**_**2**_ and polymeric **Ag**_**2**_ **(TPT-1)**_**2**_, which were characterized by nuclear magnetic resonance (NMR), ultraviolet/visible (UV/vis), Fourier-transform infrared (FT-IR), X-ray photoelectron spectroscopy (XPS), and high-resolution mass spectrometry (HRMS). Electrocatalytic activity was first investigated by homogeneous cyclic voltammetry in CH_3_CN and subsequently under heterogeneous conditions in H-type and custom-built zero-gap electrochemical cells. The silver­(I) complexes exhibited stable and selective CO_2_-to-CO conversion, achieving Faradaic efficiencies of ∼80%, energy efficiencies of 24%, and single-pass conversions of 20% at a constant current density of 200 mA cm^–2^.

## Graphical abstract

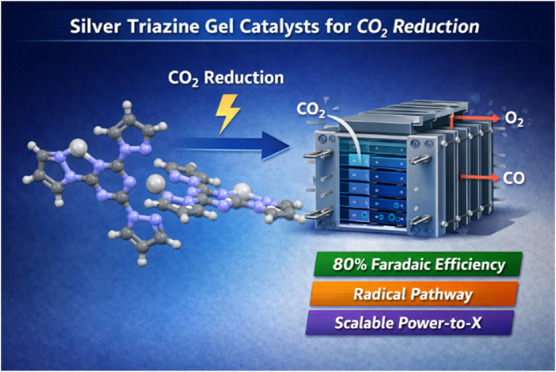

## Introduction

Nonrenewable fossil resources (petroleum, natural gas, and coal) dominate the global energy mix, accounting for roughly 81.5% of the primary energy consumption in 2023.^1^ Within decades, carbon deposited over millions of years has been released by human activity as CO_2_ into the atmosphere, disrupting the Earth’s natural carbon balance.^2^ NOAA’s Global Monitoring Laboratory reported the average atmospheric CO_2_ concentration with 422.8 ppm in 2024, marking another record high and the largest one-year increase on record at 3.75 ppm.^3^ The transformation from traditional, fossil-based energy systems to renewable energy generation is of central significance to facilitate a sustainable energy future and to address climate change, as CO_2_ emissions have been the principal driver of human-induced global warming since the onset of industrialization.^4^ However, renewable energy sources, such as solar and wind, are intermittent, hence, surplus renewable electricity must be converted into chemical energy carriers, for instance, hydrogen, chemical feedstocks, and fuels, to minimize energy losses.^2,5^ In this context, the capture and electrochemical transformation of CO_2_ into valuable products is particularly attractive, as it contributes to a circular carbon economy and reduces greenhouse gas emissions.^2,6–8^ Hence, tremendous research efforts in recent years have focused on the development of effective, stable, and selective molecular catalysts for the electrochemical reduction of CO_2_ to C_1_, C_2_, or even C_3_ products.^9–23^ Although triazine-based ligands and their metal complexes have been known for decades, their implementation as electrocatalysts for CO_2_ has remained scarce.^24,25^

Triazine-based ligands such as 2,4,6-tri-(1*H*-pyrazol-1-yl)-1,3,5-triazine **TPT-1** (Scheme 1) offer a unique platform for the design of molecular electrocatalysts due to their nitrogen-rich and electron-deficient coordination environment. The presence of multiple heteroaromatic donor sites enables strong metal–ligand interactions while maintaining an overall π-accepting framework. Importantly, **TPT-1** exhibits redox noninnocent behavior, allowing the ligand to participate directly in electron transfer processes by acting as an electron reservoir. This ligand-centered redox activity facilitates the accumulation and delivery of electrons required for CO_2_ activation, thereby promoting efficient catalytic turnover. Consequently, the incorporation of **TPT-1** provides a rational strategy to enhance catalytic performance by coupling metal-centered reactivity with ligand-assisted electron transfer.^26^

**Scheme 1:**
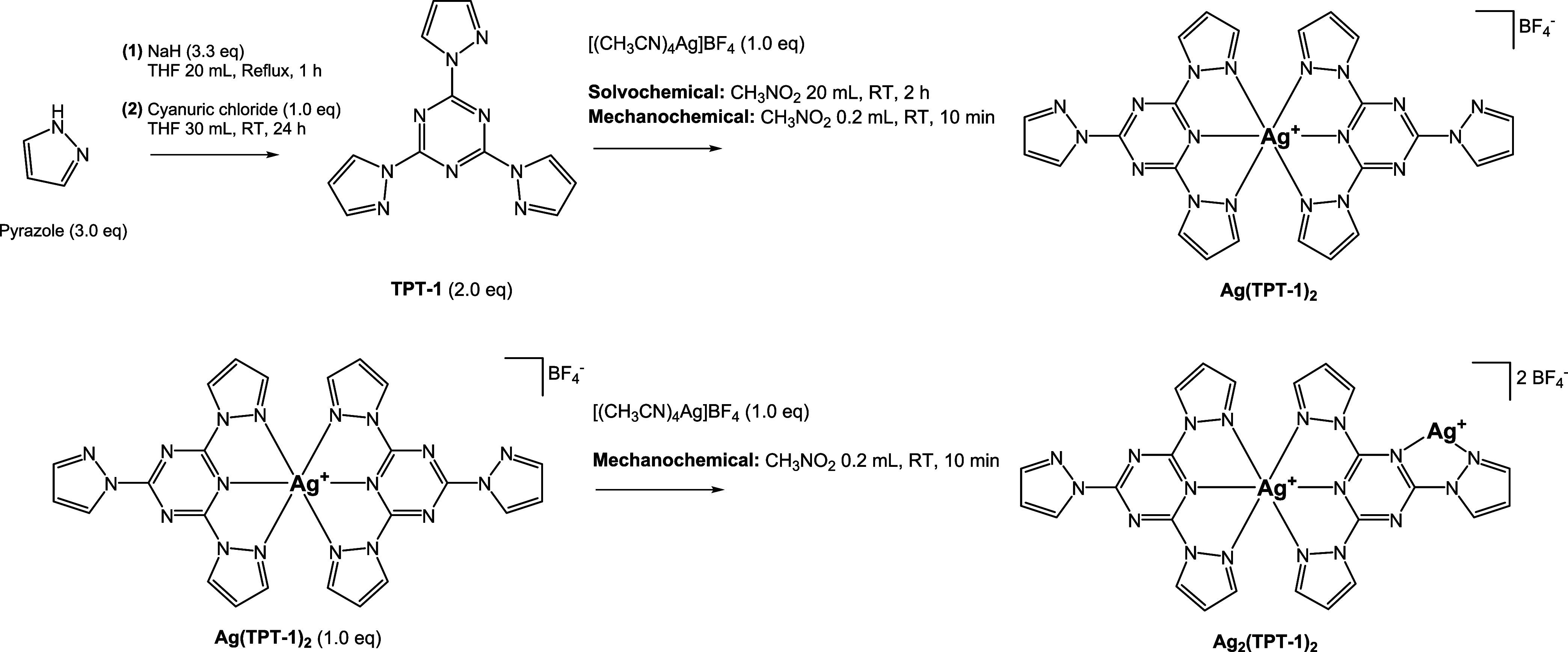
Synthesis of 2,4,6-Tri-Substituted-1,3,5-Triazine Ligands **TPT-1**, **Ag­(TPT-1)**_**2**_ Complex and Homometallic Coordination Polymer Gel Ag_2_(TPT-1)_2_

We report the synthesis and implementation of a triazine-based ligand system (2,4,6-tri-(1*H*-pyrazol-1-yl)-1,3,5-triazine) **TPT-1** (Scheme 1) and its silver­(I) complexes for electrochemical CO_2_ reduction. 2,4,6-tri-(1*H*-pyrazol-1-yl)-1,3,5-triazine (**TPT-1**) belongs to the class of tri­(azolyl)-1,3,5-triazines, for which the rigid 1,3,5-triazine core is substituted with three azole substituents such as pyrazole, imidazole, or triazole. **TPT-1** features a rigid, planar, electron-deficient 1,3,5-triazine core with three weakly electron-donating pyrazolyl substituents at the 2, 4, and 6 positions. The triazine’s nitrogen function as π-accepting centers and the pyrazolyl’s nitrogen atoms as σ-donors. However, the pyrazolyl substituents are less electron-donating than their pyridyl analogues, resulting in an electron-poor, nitrogen-rich coordination environment with unique π-accepting/σ-donating properties able to bind low-valent metals.^27,28^ In addition, the **TPT-1** ligand structure aids the formation of supramolecular or gel-like assemblies. **TPT-1** in the solid state features polymorphism and cocrystal formation, with different polymorphs stabilized by π–π-stacking and anion-π interactions.^28,29^

**TPT-1** represents a tridentate ligand with three pyrazolyl substituents, each of which incorporates two potential donor sites, more precisely, the N1 and N2 of pyrazole. However, the coordination mainly takes place through the N-bound position of pyrazole. In contrast, the triazine nitrogen stays noncoordinating or only weakly engaged. This supports both mono- and polynuclear coordination architectures. For instance, [M­(TPT-1)_2_]­X_2_, where M = Fe, Co, or Ni, was prepared by Berdiell et al. in 2018, and transformed into coordination polymer gels through the addition of silver­(I) by the interaction of the pyrazolyl arms with silver­(I).^28^ Their work emphasizes how the tridentate ligand framework of **TPT-1** can possibly be extended into supramolecular or gel-like assemblies. However, the steric congestion around the triazine-pyrazole junction due to the hydrogen atoms adjacent to the coordinating nitrogens constitutes a barrier for secondary metal coordination. This limits the formation of dinuclear and trinuclear complexes. Trinuclear assemblies of **TPT-1** are therefore exceedingly rare, in particular, when compared to their pyridyl analogues.^28,30,31^

## Experimental Section

### Materials and Reagents

Chemicals and solvents were purchased from Alfa Aesar, BLDpharm, the Fuel Cell Store, Merck, Sigma-Aldrich, Strem Chemicals, Thermo Fisher Scientific, and VWR International, and were used without further purification unless otherwise specified. Anhydrous solvents (THF and CH_3_CN) were obtained from a molecular sieve under N_2_ atmosphere (MB-SPS-7, M. Braun Inertgas-Systeme). High-purity water (18 MΩ·cm) was received from a Milli-Q Reference A+ purification system with MillipakQ-40 filter unit (0.22 μm pore size, Merck). High-purity gases (argon 5.0, carbon dioxide 4.5, and helium 5.0) were sourced from Linde Gas.

### Characterization Methods

^1^H NMR and ^13^C NMR spectra were recorded on a Bruker AVIII 300 or a Bruker AVIII 500 MHz spectrometer in deuterated solvents: CDCl_3_, DMSO-*d*_6_ (both Sigma-Aldrich), and D_2_O (Eurisotop). Chemical shifts (δ) are reported in parts per million (ppm) referenced to the residual solvent signals for ^1^H and ^13^C unless otherwise noted. Ultraviolet/visible (UV/vis) absorption spectra were acquired on a Varian Cary 300 Bio UV/vis spectrophotometer. The measurements were performed in CH_2_Cl_2_ and CH_3_CN using standard 10 mm quartz cuvettes. Fourier-transform infrared (FT-IR) absorption spectra were obtained on a Bruker α II Compact FT-IR spectrometer. X-ray photoelectron spectroscopy (XPS) spectra were measured on a Thermo Fisher Scientific Nexsa G2 Surface Analysis System, equipped with a monochromatic Al Kα source (*h*ν = 1486.6 eV). The pressure within the analysis chamber was maintained at ultrahigh vacuum (∼10^–9^ mbar). Powder samples were tightly pressed onto indium foil to ensure good electrical contact with the sample holder. XPS survey spectra were recorded over the binding energy range 0–1400 eV with 200 eV pass energy and 30 s dwell time. High-resolution XPS spectra of C 1s, N 1s, F 1s, Cl 2p, Ag 3d, and AgMNN for chemical state analysis were recorded with 20 eV pass energy and 30 s dwell time. The binding energies were referenced to the C 1s signal at 285 eV to correct for possible charging effects. Quantitative elemental compositions (atom %) were determined from the recorded survey spectra by peak area integration using instrument-specific atomic sensitivity factors. Data analysis, including peak fitting and background subtraction, was performed in Thermo Scientific Avantage Software. HRMS-ESI spectra were recorded on an Agilent 6520 Q-TOF mass spectrometer with ESI source, Agilent G1607A coaxial sprayer, and Thermo Fisher Scientific LTQ Orbitrap XL with Ion Max API source in positive mode. Samples were prepared as particle-free solutions in CH_2_Cl_2_ or CH_3_CN.

### Synthesis of Ag­(TPT-1)_2_ and Coordination Polymer Ag_2_(TPT-1)_2_

The reaction protocol involved the solvochemical and mechanochemical synthesis of the mononuclear metal complexes **Ag­(TPT-1)**_**2**_ and the corresponding polymeric complex **Ag**_**2**_ **(TPT-1)**_**2**_ (Scheme 1). All analytical data of the ligand **TPT-1**, complex **Ag­(TPT-1)**_**2**_, and the coordination polymer **Ag**_**2**_ **(TPT-1)**_**2**_ are depicted in the Supporting Information (Figures S1–S38).

#### 2,4,6-Tri-(1*H*-pyrazol-1-yl)-1,3,5-triazine (TPT-1)

NaH (284 mg, 11.8 mmol, 3.3 equiv) was dispersed in dry THF (20 mL) under a N_2_ atmosphere, and pyrazole (731 mg, 10.7 mmol, 3.0 equiv) was added portionwise. The mixture was stirred at room temperature until hydrogen evolution ceased, then heated at reflux for 1 h. Upon cooling to room temperature, a solution of cyanuric chloride (660 mg, 3.58 mmol, 1.0 equiv) in dry THF (10 mL) was added dropwise. The mixture was stirred at room temperature for 24 h, then THF was removed by rotary evaporation. The crude product was washed with distilled water (3 × 10 mL), collected by suction filtration, and dried under vacuum for 24 h to obtain ligand **TPT-1** as a light beige powder (720 mg, 2.58 mmol, 72%). ^1^H NMR (500 MHz, CDCl_3_, 298 K) δ/ppm: 6.60 (dd, *J* ^1^ = 2.8 Hz, *J* ^2^ = 1.5 Hz, 3 H, ArH), 7.97 (d, *J* ^1^ = 0.9 Hz, 3 H, ArH), 8.81 (d, *J* ^1^ = 2.8 Hz, 3 H, ArH), ^1^H NMR (300 MHz, (CD_3_)_2_SO, 298 K) δ/ppm: 6.76 (dd, *J* ^1^ = 2.8 Hz, *J* ^2^ = 1.5 Hz, 3 H, ArH), 8.06 (d, *J* ^1^ = 0.9 Hz, 3 H, ArH), 8.99 (d, *J* ^1^ = 2.7 Hz, 3 H, ArH), ^13^C NMR (126 MHz, CDCl_3_, 298 K) δ/ppm: 110.6, 130.9, 146.3, 163.9, UV/vis (CH_2_Cl_2_ and CH_3_CN, 298 K) λ_max_/nm: CH_2_Cl_2_: 268 and CH_3_CN: 265, FT-IR (ATR, 298 K) ν/cm^–1^: 3147, 3108, 1571, 1527, 1511, 1437, 1385, 1309, 1297, 1270, 1254, 1223, 1202, 1184, 1147, 1087, 1077, 1050, 1033, 941, 908, 867, 801, 772, 753, 644, 599, XPS (Al Kα, 1486.6 eV, calibrated to C 1s = 285 eV): C 1s (285 eV, 62.3%), N 1s (400 eV, 37.7%), HRMS-ESI (positive mode, *t*_R_ = 0.263 min): *m*/*z* calcd for ligand C_12_H_10_N_9_^+^ = 280.1054 [*M*+H]^+^, *m*/*z* measured = 280.1089 [*M*+H]^+^.

#### Di-2,4,6-tri-(1*H*-pyrazol-1-yl)-1,3,5-triazine-silver­(I)-tetrafluoroborate (Ag­(TPT-1)_2_)

**TPT-1** (148 mg, 0.53 mmol, 2.0 equiv), of [(CH_3_CN)_4_Ag]­BF_4_ (95 mg, 0.27 mmol, 1.0 equiv) and CH_3_NO_2_ (0.2 mL) were placed in a 4 mL glass milling vessel together with one 5 mm stainless-steel ball. The mixture was milled at room temperature for 10 min at 3000 rpm. The solvent was removed, the crude product washed with Et_2_O (3 × 10 mL), collected by suction filtration, and dried under vacuum for 24 h to afford complex **Ag­(TPT-1)**_**2**_ as a light beige powder (186 mg, 0.25 mmol, 93%). ^1^H NMR (500 MHz, CDCl_3_, 298 K) δ/ppm: 6.63 (dd, *J* ^1^ = 1.5 Hz, 1 H, ArH), 7.94 (s, 1 H, ArH), 8.82 (d, *J* ^1^ = 2.8 Hz, 1 H, ArH), ^1^H NMR (300 MHz, (CD_3_)_2_SO, 298 K) δ/ppm: 6.81 (s, 1 H, ArH), 8.10 (s, 1 H, ArH), 9.06 (d, *J* ^1^ = 2.3 Hz, 1 H, ArH), UV/vis (CH_2_Cl_2_ and CH_3_CN, 298 K) λ_max_/nm: CH_2_Cl_2_: 269 and CH_3_CN: 265, FT-IR (ATR, 298 K) ν/cm^–1^: 3565, 3481, 3143, 2832, 1741, 1696, 1675, 1643, 1575, 1529, 1447, 1389, 1270, 1249, 1229, 1212, 1192, 1147, 1062, 1029, 951, 939, 908, 805, 770, 700, 655, 636, 595, 554, 519, 498, 482, 463, 443, 430, 422, 406, XPS (Al Kα, 1486.6 eV, calibrated to C 1s = 285 eV): C 1s (285 eV, 51.0%), N 1s (400 eV, 36.5%), F 1s (685 eV, 9.8%), Ag 3d (368 eV, 2.8%), HRMS-ESI (positive mode, *t*_R_ = 0.253 min): *m*/*z* calcd for complex C_24_H_18_AgN_18_^+^ = 665.1008 [*M*]^+^, *m*/*z* measured = 665.1025 [*M*]^+^.

#### Di-2,4,6-tri-(1*H*-pyrazol-1-yl)-1,3,5-triazine-silver­(I)­silver­(I)-tetrafluoroborate (Ag_2_(TPT-1)_2_)

**Ag­(TPT-1)**_**2**_ (159 mg, 0.21 mmol, 1.0 equiv), AgBF_4_ (41 mg, 0.21 mmol, 1.0 equiv), and CH_3_NO_2_ (0.2 mL) were placed in a 4 mL glass milling vessel together with one 5 mm stainless-steel ball. The mixture was milled at room temperature for 10 min at 3000 rpm. The solvent was removed, the crude product washed with Et_2_O (3 × 10 mL), collected by suction filtration, and dried under vacuum for 24 h to afford complex **Ag**_**2**_ **(TPT-1)**_**2**_ as a gray powder (187 mg, 0.20 mmol, 94%). ^1^H NMR (300 MHz, (CD_3_)_2_SO, 298 K) δ/ppm: 6.86 (s, 1 H, ArH), 8.15 (s, 1 H, ArH), 9.11 (s, 1 H, ArH), UV/vis (CH_2_Cl_2_ and CH_3_CN, 298 K) λ_max_/nm: CH_2_Cl_2_: 272 and CH_3_CN: 265, FT-IR (ATR, 298 K) ν/cm^–1^: 3197, 3157, 3137, 3499, 2357, 2260, 1651, 1638, 1575, 1525, 1515, 1449, 1396, 1365, 1291, 1247, 1196, 1153, 1087, 1044, 965, 935, 914, 883, 807, 797, 780, 766, 727, 661, 636, 628, 595, 545, 523, 494, 476, 451, 422, 414, XPS (Al Kα, 1486.6 eV, calibrated to C 1s = 285 eV): C 1s (285 eV, 37.6%), N 1s (400 eV, 31.1%), F 1s (686 eV, 22.4%), Ag 3d (368 eV, 8.9%), HRMS-ESI (positive mode, *t*_R_ = 0.361 min): *m*/*z* calcd for complex C_24_H_18_Ag_2_N_18_^+^ = 386.0027 [*M*]^2+^, *m*/*z* measured = 386.0023 [*M*]^2+^.

### Catalyst Ink and Electrode Preparation

For the electrode preparation, carbon cloth W1S1011 (Fuel Cell Store) was spray-coated with catalyst ink containing 2 mg mL^–1^ catalyst, 1 mg mL^–1^ ENSACO 250G carbon black, and 10 μL mL^–1^ Piperion dispersion (5 wt %, Fuel Cell Store) in a 5:1 2-propanol/18 MΩ water mixture. High-porosity titanium fiber felt (Fuel Cell Store) was spray-coated using catalyst inks with 2 mg mL^–1^ iridium black and 10 μL mL^–1^ Piperion dispersion (5 wt %, Fuel Cell Store).

The catalyst inks were sprayed with a Worx MakerX Akku-Airbrush WX742.9 (pressure = 0.10–0.12 MPa) on an 80 °C hot plate, and ultrasonicated continuously to maintain a stable dispersion. Weighing of the electrodes before and after spray-coating was used to confirm the intended catalyst loading of 0.5 mg cm^–2^ and iridium black loading of 1.0 mg cm^–2^.

## Result and Discussion

### Cyclic Voltammetry and CO_2_ Electrocatalysis

Cyclic voltammetry experiments were performed under homogeneous conditions in CH_3_CN with 0.1 M TBAPF_6_ as supporting electrolyte to investigate the intrinsic redox behavior of the **TPT-1** ligand and its complexes. Accordingly, they are not intended to directly reproduce the heterogeneous electrolysis in the H-type and zero-gap electrochemical cells.

The cyclic voltammogram of **TPT-1** in dry CH_3_CN under an argon atmosphere demonstrates two distinct reductive features (Figure 1a), a relatively small but clearly visible peak at −0.6 V vs NHE and a large peak at −1.6 V vs NHE. While the first cathodic peak at −0.6 V vs NHE is accompanied by an anodic return peak, the second cathodic peak at −1.6 V vs NHE exhibits only a weak anodic response on the reverse scan and must be considered as electrochemically irreversible. Both cathodic peaks can be attributed to one-electron reductions on the ligand scaffold, indicating radical anion and diradical dianion formation.^24–26^

**Figure 1:**
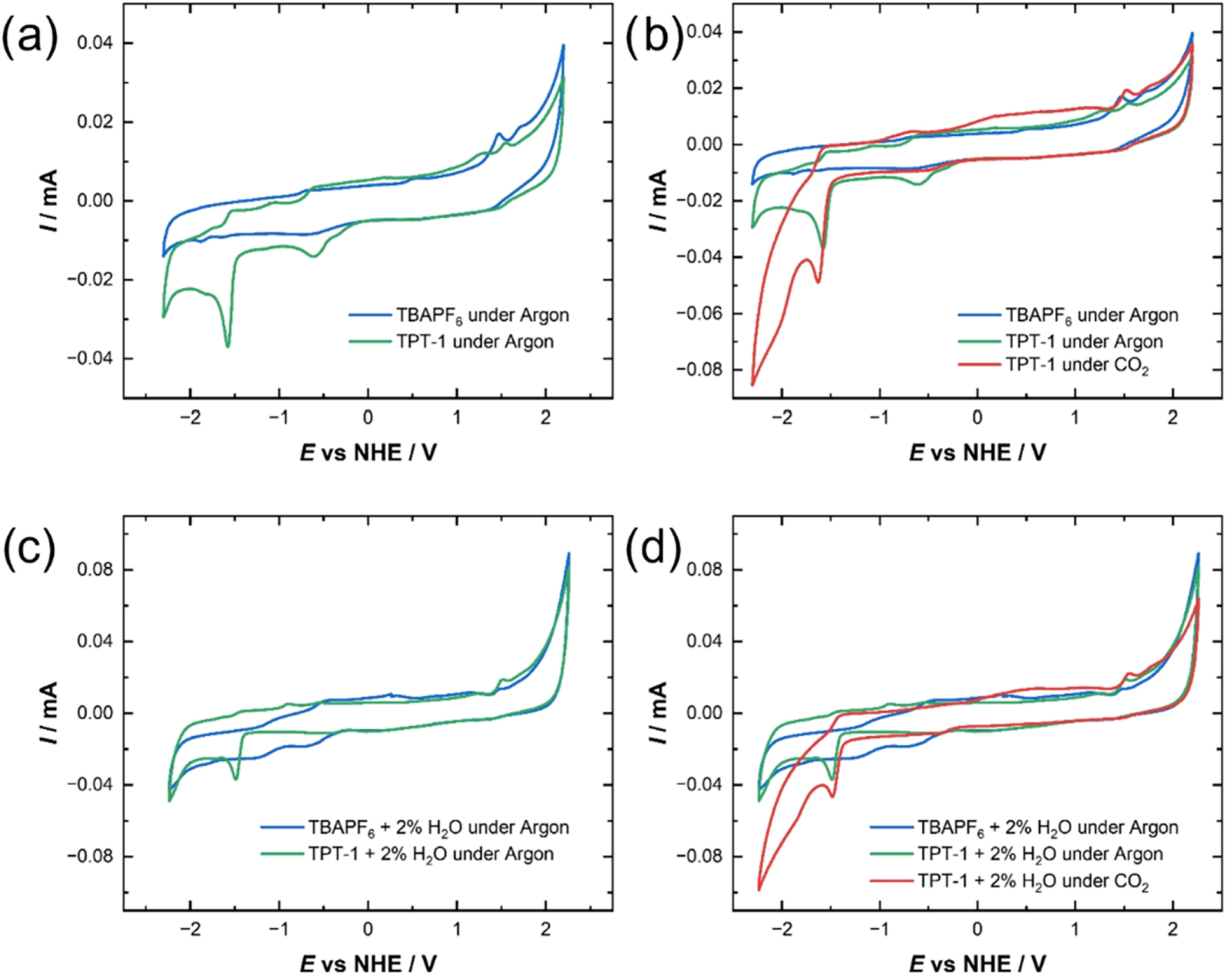
Cyclic voltammograms of ligand **TPT-1** in CH_3_CN containing 0.1 M TBAPF_6_ as supporting electrolyte under (a) argon atmosphere, (b) CO_2_ atmosphere, (c) argon atmosphere with 2% water as proton source, and (d) CO_2_ atmosphere with 2% water as proton source.

Upon saturation with CO_2_, the cathodic peak at −0.6 V vs NHE is no longer visible, while the cathodic peak at −1.6 V maintains its form and position (Figure 1b). The addition of 2% 18 MΩ water as proton source results in no significant change of the voltammograms under both argon and CO_2_ atmospheres. In the recorded potential window, the maximum negative current was achieved under the CO_2_ atmosphere with 2% water (−0.09 mA) (Figure 1c,d). In summary, ligand **TPT-1** exhibits a small reversible first and a large irreversible second reduction. None of the two reductions demonstrates a sustained catalytic response in the presence of CO_2_, as a consequence, it does not efficiently catalyze CO_2_ reduction on its own.

The cyclic voltammograms of the silver­(I) complex **Ag­(TPT-1)**_**2**_ differ substantially from those of the free ligand **TPT-1** with respect to their electrochemical behavior toward CO_2_ reduction. Under an argon atmosphere, only a weak second cathodic feature is observed at approximately −1.4 V vs NHE (Figure 2a). In addition, the small reversible redox signal at approximately −0.6 V vs NHE cannot be assigned unambiguously and may originate from ligand-centered or metal-influenced redox processes.

**Figure 2:**
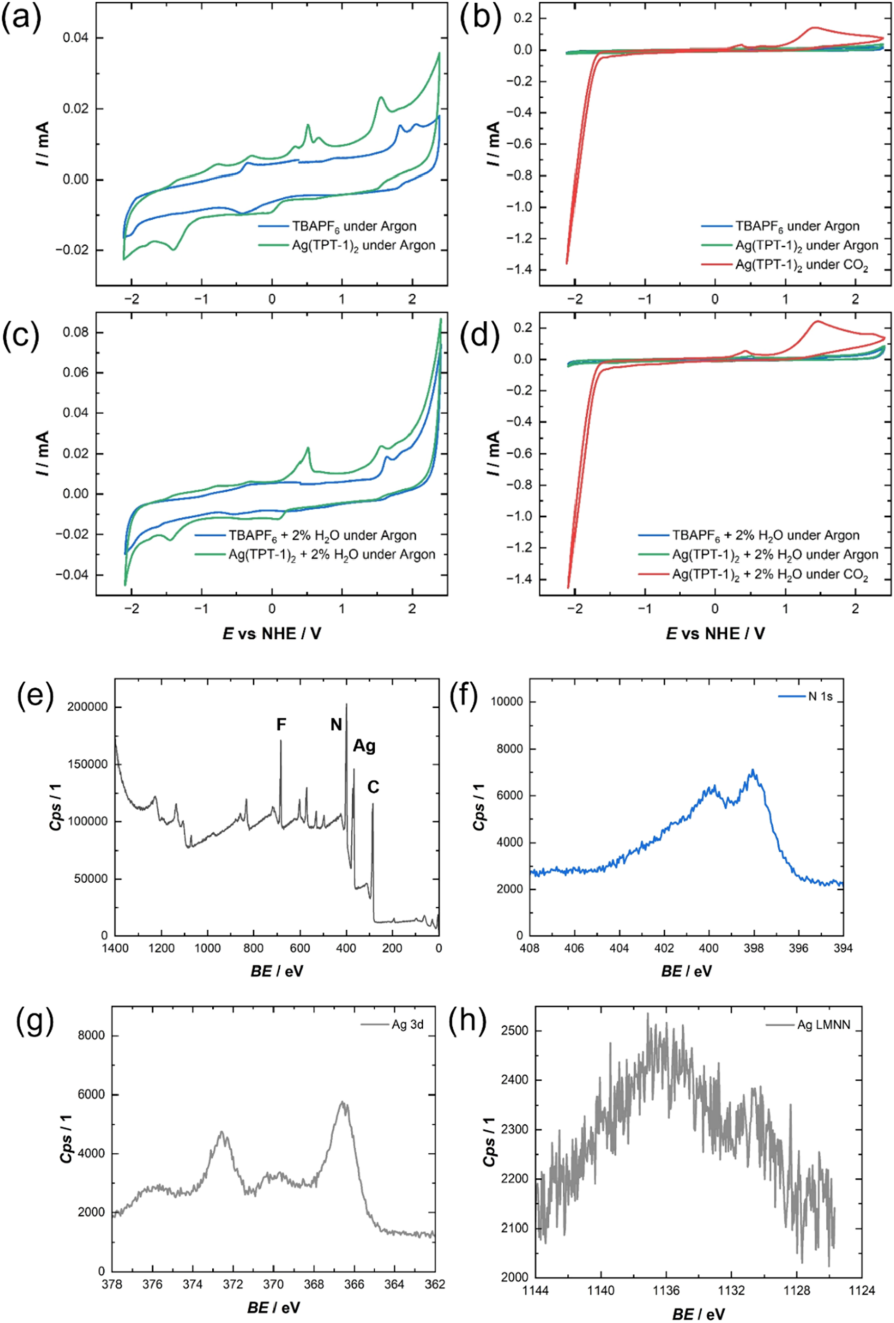
Cyclic voltammograms of complex **Ag­(TPT-1)**_**2**_ in CH_3_CN containing 0.1 M TBAPF_6_ as supporting electrolyte under (a) argon atmosphere, (b) CO_2_ atmosphere, (c) argon atmosphere with 2% water as proton source, and (d) CO_2_ atmosphere with 2% water as proton source. XPS of the as-prepared catalyst material (e) XPS survey spectrum of complex **Ag­(TPT-1)**_**2**_ recorded over the range 0–1400 eV. (f) High-resolution XPS spectrum of complex **Ag­(TPT-1)**_**2**_ for N 1s recorded in the range 394–408 eV. (g) High-resolution XPS spectrum of complex **Ag­(TPT-1)**_**2**_ for Ag 3d recorded in the range 362–378 eV. (h) High-resolution XPS spectrum of complex **Ag­(TPT-1)**_**2**_ for Ag LMNN recorded in the range 1124–1144 eV. The carbon C 1s peak at 285 eV was used as the reference for binding energy calibration.

Upon saturation with CO_2_, the first cathodic peak disappears, suggesting that the initially generated reduced ligand species undergoes rapid subsequent chemical reactions in the presence of dissolved CO_2_. As a consequence, the reversibility of this redox process is suppressed, and the corresponding oxidation feature is no longer observed during the reverse scan. Most notably, CO_2_ saturation leads to a pronounced increase in the cathodic current, resulting in a steep catalytic wave with current densities reaching approximately −1.4 mA (Figure 2b).

This catalytic response is substantially larger than that observed for the free ligand TPT-1, which exhibits a maximum cathodic current of only −0.09 mA under comparable conditions. The significantly enhanced current response upon CO_2_ introduction is consistent with catalytic electrochemical CO_2_ reduction mediated by the Ag­(I) complex. Nevertheless, cyclic voltammetry alone does not provide definitive proof of catalytic CO_2_ reduction and must be interpreted together with the product analysis obtained from the H-type cell and zero-gap electrolyzer experiments.

The addition of 2% 18 MΩ water as proton source results in no significant change for the cyclic voltammograms under both argon and CO_2_ atmospheres. In wet electrolyte, the steep cathodic rise is only marginally stronger, indicating that water is not essential for the CO_2_ electrocatalysis with complex **Ag­(TPT-1)**_**2**_ (Figure 2c,d).

The survey XPS spectrum and high-resolution XPS spectra of the **Ag­(TPT-1)**_**2**_ complex were recorded to evaluate its chemical state as-prepared, before and after electrolysis (Figures 2 e–h and S55–56). The high-resolution N 1s spectrum was collected in the binding energy range of 394–408 eV. The Ag 3d spectrum was recorded in the range of 362–378 eV, while the Ag LMNN Auger spectrum was measured between 1124–1144 eV. Comparison of the spectra obtained before and after electrolysis revealed no significant changes in peak position or shape. These observations do not indicate major changes in the chemical environment at the electrode surface during electrolysis.

Complex **Ag­(TPT-1)**_**2**_ was finally tested in the custom-built zero-gap electrochemical cell at 50, 100, and 200 mA cm^–2^ with a relative humidity of 50% and CO_2_ feed of 50 mL min^–1^ (Figures 3b,c, 4a–d, and S50–S52). The chronopotentiometric potential-time curves remained stable over the measurement time of 60 min, confirming the robustness of the catalyst under sustained current load (Figure 3c). Most importantly, however, the Faradaic efficiencies increased from 57.9% at 50 mA cm^–2^ to 70.1% at 100 mA cm^–2^ and finally to 77.1% at 200 mA cm^–2^ (Figure 4a). Faradaic efficiencies for CO featured a continuous increase from low to higher current densities, although for other well-established catalyst materials such as silver nanoparticles, the opposite is observed (Figure S54). The CO selectivity and effective conversion of CO_2_ by complex **Ag­(TPT-1)**_**2**_ were reflected in comparably high energy efficiencies of 23.5–25.0%, molar production rates of 2.9 mmol h^–1^ cm^–2^, and single-pass conversions up to 19.0% for CO at 200 mA cm^–2^ (Figure 4b–d). Accordingly, the outlet gas stream consisted primarily of unreacted CO_2_, together with CO as the major reduction product and minor quantities of H_2_, as reflected by the measured Faradaic efficiencies and single-pass conversions.

**Figure 3:**
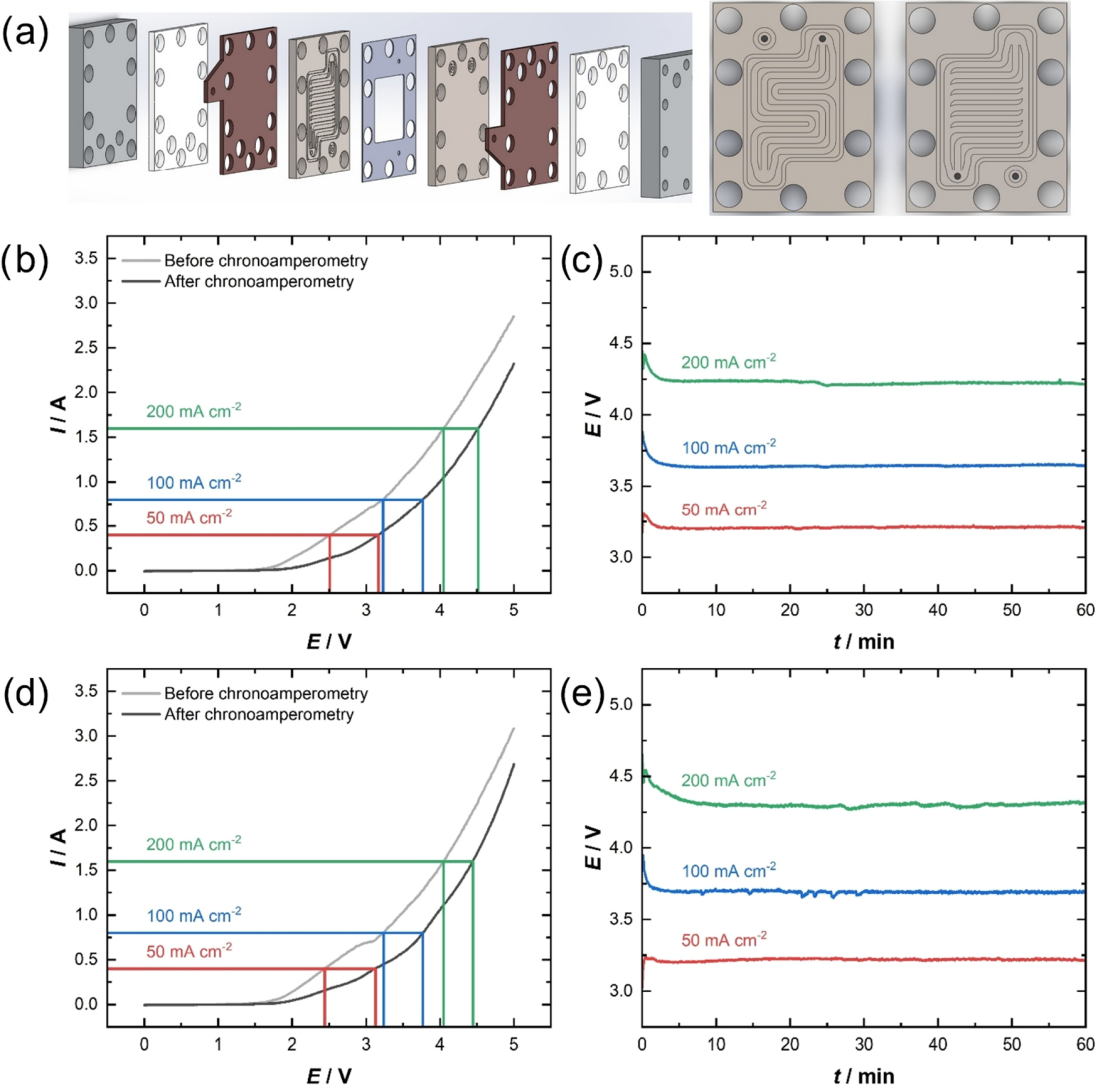
(a) Custom-built CO_2_ electrolyzer cell (JKU ZONA Workshop) (b) and (d) linear sweep voltammogram of complexes **Ag­(TPT-1)**_**2**_ and **Ag**_**2**_ **(TPT-1)**_**2**_ recorded before and after chronopotentiometry, indicating the effect of extended electrolysis on the catalytic response. (c) and (e) Chronopotentiometric potential-time curves (*E-t*) at constant current densities of 50, 100, and 200 mA cm^–2^, showing the required operating potentials under different current loads.

**Figure 4:**
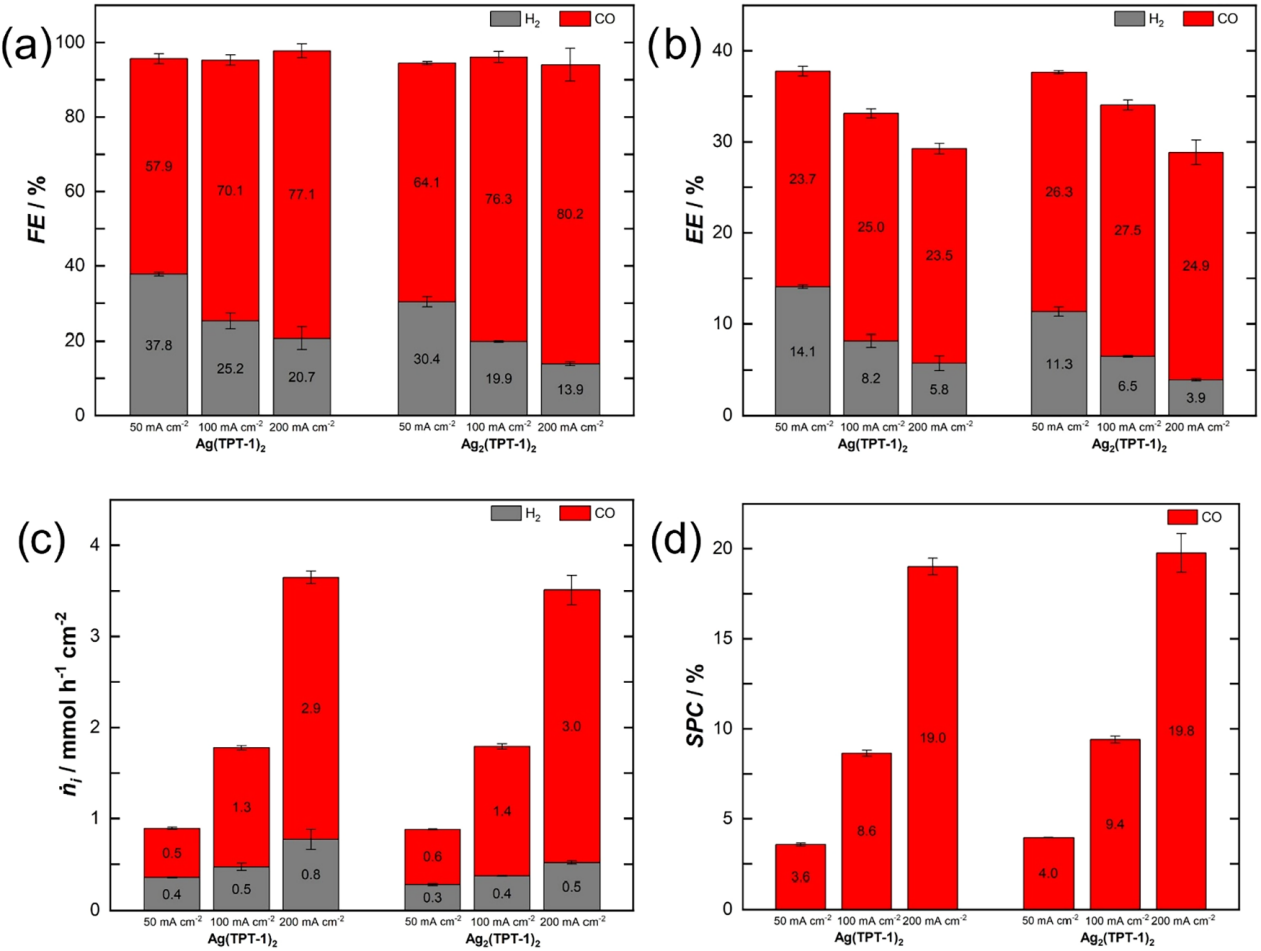
(a) Faradaic efficiencies for H_2_ and CO, demonstrating selective CO formation at higher current densities. (b) Energy efficiencies for H_2_ and CO, revealing the trade-off between product selectivity and energy input. (c) Molar production rates of H_2_ and CO normalized to the geometrical electrode area (8 cm^2^), highlighting markedly increased CO formation at 200 mA cm^–2^. (d) Single-pass conversion for CO, reflecting increased CO_2_ utilization at 200 mA cm^–2^. Measurements were conducted in a custom-built CO_2_ electrolyzer cell (JKU ZONA Workshop) operated at 60 °C, with a CO_2_ inlet stream of 50 mL min^–1^ at 50% *RH* and an anolyte flow rate of 100 mL min^–1^ 0.1 M CsOH. Error bars represent the standard deviation from independent measurements. Taken together, graphs (a–d) demonstrate the CO selectivity and effective CO_2_ conversion of complexes **Ag­(TPT-1)**_**2**_ and **Ag**_**2**_ **(TPT-1)**_**2**_ at elevated current densities, emphasizing their potential as efficient catalysts for electrochemical CO_2_ reduction.

Additional long-term chronopotentiometric measurements were conducted over 30 h, confirming the robustness of the catalyst under sustained current load (Figure S53).

The cyclic voltammograms of the bimetallic silver­(I) complex **Ag**_**2**_ **(TPT-1)**_**2**_ differ also significantly when compared to ligand **TPT-1** or its monometallic silver­(I) analogue **Ag­(TPT-1)**_**2**_ (Figures 1–2). Whereas complex **Ag­(TPT-1)**_**2**_ features only a small peak at −1.4 V vs NHE for the second cathodic reduction, three cathodic peaks at +1.7 V, −0.2 V, and −1.6 V vs NHE and two anodic peaks at +0.5 V and +1.5 V vs NHE can be observed for complex **Ag**_**2**_ **(TPT-1)**_**2**_. The irreversible cathodic reduction peak found at −1.6 V vs NHE can again be assigned to the second cathodic reduction peak of ligand **TPT-1**, the pronounced cathodic reduction peak at −0.2 V and anodic oxidation peak at +0.5 V to the reduction from Ag^+^ to Ag^0^ and the reoxidation from Ag^0^ to Ag^+^, respectively. These peaks as well as the cathodic and anodic features at even more positive potentials can be attributed to the plating and stripping of silver. Consequently, it is possible that complex **Ag**_**2**_ **(TPT-1)**_**2**_ disassembles during homogeneous catalysis, releasing Ag^+^ from the minor coordination site. However, under CO_2_ atmosphere, a steep increase in cathodic current down to approximately −0.8 mA and −1.0 mA can be observed in dry and wet CH_3_CN (Figure S47).

Measurements in the custom-built zero-gap electrochemical cell for the bimetallic silver­(I) complex **Ag**_**2**_ **(TPT-1)**_**2**_ were again performed at current densities of 50, 100, and 200 mA cm^–2^ with a relative humidity of 50% and CO_2_ feed of 50 mL min^–1^ (Figure 4a–d). Chronopotentiometric potential-time curves showed stable potentials at 3.21 V, 3.65, and 4.24 V, confirming its stability and weakening opposite considerations from the initially conducted cyclic voltammetry in CH_3_CN (Figure 3d). The Faradaic efficiencies increased from 64.1% at 50 mA cm^–2^, to 76.3% at 100 mA cm^–2^, and finally to 80.2% at 200 mA cm^–2^ (Figure 4a). In comparison with the monometallic silver­(I) analogue **Ag­(TPT-1)**_**2**_, bimetallic silver­(I) complex **Ag**_**2**_ **(TPT-1)**_**2**_ was not able to yield a significant increase in Faradaic efficiency for CO at 200 mA cm^–2^, despite the incorporation of a second silver­(I) metal center into the minor coordination site of ligand **TPT-1**. Nevertheless, higher Faradaic efficiencies could be obtained for 50 mA cm^–2^ and 100 mA cm^–2^ with differences of 6% on average. Similar to complex **Ag­(TPT-1)**_**2**_, the CO selectivity and effective conversion of CO_2_ by complex **Ag**_**2**_ **(TPT-1)**_**2**_ are reflected in energy efficiencies of 24.9–27.5%, molar production rates of 3.0 mmol h^–1^ cm^–2^, and single-pass conversions for CO_2_ of up to 19.8% at 200 mA cm^–2^ (Figure 4b–d). Under the investigated zero-gap electrolysis conditions, **Ag­(TPT-1)**_**2**_ molecular complex and the **Ag**_**2**_ **(TPT-1)**_**2**_ polymeric gel achieved higher CO Faradaic efficiencies at lower Ag loadings than Ag nanoparticles, which are commonly employed as a benchmark electrocatalyst for CO_2_-to-CO conversion (Figures S50, S51, and S54). The observed increase in CO Faradaic efficiency with increasing current density represents an unusual trend compared to conventional silver nanoparticle catalysts, where higher current densities typically favor the competing hydrogen evolution reaction (HER). In the present system, this behavior can be rationalized by a combination of local reaction environment effects and intrinsic catalyst properties. At elevated current densities, the rapid consumption of protons at the cathode leads to a significant increase in the local pH within the catalyst layer. This alkalization suppresses proton availability at the electrode interface, thereby inhibiting HER and shifting the selectivity toward CO_2_ reduction. In addition, the molecular coordination environment provided by the **TPT-1** ligand framework likely plays a decisive role. The ligand’s redox noninnocent character enables efficient electron storage and transfer, facilitating the stabilization of key CO_2_ reduction intermediates while disfavoring proton reduction pathways. Furthermore, the polymeric gel structure may impose mass transport constraints that limit proton diffusion relative to CO_2_, reinforcing the suppression of HER under high current operation. The combination of these effects—local pH modulation, restricted proton transport, and ligand-mediated intermediate stabilization—provides a plausible explanation for the enhanced CO selectivity observed at higher current densities in this system.^16,17^

### Proposed Mechanism of CO_2_ Reduction

The observed current at *E*_pc_ = −1.60 V vs NHE is related to the electrocatalytic CO_2_ reduction according to the following reactions (only one ligand considered for electroreduction and subsequent CO_2_ activation and CO formation)


I $${\left[\mathrm{AgI}{\left(\mathrm{TPT}\right)}_{2}\right]}^{+}+e^{-}\to \left[\mathrm{AgI}\left(\mathrm{TPT}\right)\left({\mathrm{TPT}}^{-}\right)\right]$$

II $$\left[{\mathrm{Ag}}^{I}\left(\mathrm{TPT}\right)\left({\mathrm{TPT}}^{•-}\right)\right]+e^{-}\to \left[{\mathrm{Ag}}^{I}\left(\mathrm{TPT}\right)\left({\mathrm{TPT}}^{-}\right)\right]$$

III $$\left[{\mathrm{Ag}}^{I}\left(\mathrm{TPT}\right)\left({\mathrm{TPT}}^{-}\right)\right]+{\mathrm{CO}}_{2}\to \left[{\mathrm{Ag}}^{I}\left(\mathrm{TPT}\right)\left(\mathrm{TPT}\right)\left({\mathrm{CO}}_{2}^{-}\right)\right]$$

IV $$\left[{\mathrm{Ag}}^{I}\left(\mathrm{TPT}\right)\left(\mathrm{TPT}\right)\left({\mathrm{CO}}_{2}^{-}\right)\right]+e^{-}⇌\left[\mathrm{AgI}\left(\mathrm{TPT}\right)\left({\mathrm{TPT}}^{•-}\right)\left({\mathrm{CO}}_{2}^{-}\right)\right]$$

V $$\left[{\mathrm{Ag}}^{I}\left(\mathrm{TPT}\right)\left({\mathrm{TPT}}^{•-}\right)\left({\mathrm{CO}}_{2}^{-}\right)\right]+e^{-}\to \left[{\mathrm{Ag}}^{I}\left(\mathrm{TPT}\right)\left({\mathrm{TPT}}^{-}\right)\left({\mathrm{CO}}_{2}^{-}\right)\right]$$

VI $$\left[{\mathrm{Ag}}^{I}\left(\mathrm{TPT}\right)\left({\mathrm{TPT}}^{-}\right)\left({\mathrm{CO}}_{2}^{-}\right)\right]+{\mathrm{CO}}_{2}\to \left[{\mathrm{Ag}}^{I}\left(\mathrm{TPT}\right)\left(\mathrm{TPT}\right)\left(\mathrm{CO}\right)\right]+{\mathrm{CO}}_{3}^{2-}$$

VII $$\left[{\mathrm{Ag}}^{I}\left(\mathrm{TPT}\right)\left(\mathrm{TPT}\right)\left(\mathrm{CO}\right)\right]\to {\left[{\mathrm{Ag}}^{I}{\left(\mathrm{TPT}\right)}_{2}\right]}^{+}+\mathrm{CO}$$

The hypothetical and literature-supported mechanistic proposal for the electrochemical reduction of CO_2_ by **Ag­(TPT-1)**_**2**_ and **Ag**_**2**_ **(TPT-1)**_**2**_ proceeds through ligand-centered reductions followed by metal-assisted substrate activation.^25,32–34^ A key mechanistic feature is the redox noninnocence of the TPT-based ligands, which function as electron reservoirs that enable subsequent CO_2_ reduction at the silver center. Upon application of a cathodic potential, the complexes first undergo two sequential one-electron reductions. Importantly, these reductions are localized on the coordinated TPT ligands rather than at the Ag­(I) center. In the first reduction step, one ligand accepts an electron to form a radical anion, generating a species best described as Ag­(I) coordinated to one neutral TPT ligand and one TPT^•–^ ligand.^26^ EPR spectroscopy provides strong evidence for the formation of this anion radical species, characterized by a distinctive dispersive signal with a *g*-value of 2.004, consistent with a free electron (Figure S48). This signal confirms extensive delocalization of the unpaired electron across the triazine π-system, a feature integral to its function as a redox-active center in CO_2_ reduction. A second one-electron reduction may then occur at the remaining ligand, potentially affording a diradical dianionic species in which both ligands are present as radical anions. Direct experimental evidence for this intermediate is currently unavailable. Throughout these initial electrochemical steps, the silver center remains formally in the + I oxidation state. The doubly reduced complex thus formed contains two electrons stored on the ligand framework. This reduced state is crucial for catalytic activity, as it provides the electron density necessary for substrate activation. Once generated, the electron-rich species is capable of binding CO_2_ at the Ag­(I) center. Coordination of CO_2_ occurs through the electrophilic carbon atom, with silver acting as a Lewis acidic site that facilitates substrate association and activation. Following CO_2_ coordination, an intramolecular two-electron transfer is proposed to occur from the reduced ligands to the bound CO_2_ molecule. In this step, the two electrons previously localized on the ligand-based radical anions are transferred to CO_2_, reducing it to a CO_2_^2–^ unit while the ligands are regenerated to their neutral state. This process may lead to the formation of a metallocarboxylate intermediate, formulated as [Ag­(I)­(TPT)_2_(CO_2_^2–^)]^−^. This intermediate represents the key activated form of CO_2_ within the catalytic cycle. The metallocarboxylate intermediate is proposed to subsequently undergo C–O bond cleavage, ultimately leading to CO formation. The reduction and activation of CO_2_ within the coordination sphere lowers the barrier for this bond-breaking step. After release of CO, the original Ag­(I) complex is regenerated, allowing the catalyst to re-enter the cycle. In the presence of trace proton sources, the remaining oxygen-containing fragment is protonated to give water or hydroxide, depending on the reaction conditions. Overall, the catalytic cycle involves (i) two sequential ligand-centered reductions, (ii) CO_2_ coordination at Ag­(I), (iii) intramolecular two-electron transfer from the ligand framework to the coordinated CO_2_, (iv) formation of a metallocarboxylate intermediate, and (v) C–O bond cleavage to yield CO with regeneration of the Ag­(I) catalyst. The efficiency of both **Ag­(TPT-1)**_**2**_ and **Ag**_**2**_ **(TPT-1)**_**2**_ arises from the ability of the TPT ligands to act as redox-active electron reservoirs, enabling controlled delivery of two electrons to CO_2_ while maintaining the silver center in its Ag­(I) oxidation state.

## Conclusion

The underlying manuscript reports the synthesis and implementation of a triazine-based ligand system (2,4,6-tri-(1*H*-pyrazol-1-yl)-1,3,5-triazine) **TPT-1** and silver­(I) complexes for electrochemical CO_2_ reduction. Ligand **TPT-1** was synthesized by standard literature procedures,^27,35^ following the heteroaryl nucleophilic substitution of Cl on cyanuric chloride by pyrazolate and the NaH-catalyzed cyclotrimerization of 2-cyanopyridine, respectively. Silver­(I) complexes were obtained by solvo- and mechanochemical pathways, with ball-milling achieving yields of 89–95%, reducing reaction times and solvent consumption simultaneously. The combined characterization by spectroscopic methods (NMR, UV/vis, FT-IR, XPS, and HRMS-ESI) confirmed the identity and purity of all investigated ligands and complexes. Cyclic voltammograms of ligand **TPT-1** in dry and wet CH_3_CN under both argon and CO_2_ atmosphere demonstrated a first reversible one-electron reduction and a second irreversible one-electron reduction, consistent with the computational DFT studies for these types of complexes reported in literature.^24,25^ Under practically relevant current densities of 50, 100, and 200 mA cm^–2^ in the custom-built zero-gap electrochemical cell, similar results were obtained for the monometallic complex **Ag­(TPT-1)**_**2**_ and bimetallic complex **Ag**_**2**_ **(TPT-1)**_**2**_ with Faradaic efficiencies of 77.1% and 80.2% at 200 mA cm^–2^. Comparing the electrochemical data for monometallic complex **Ag­(TPT-1)**_**2**_ and bimetallic complex **Ag**_**2**_ **(TPT-1)**_**2**_ indicates no significant benefit by introducing a second silver­(I) metal center to the minor coordination site of ligand **TPT-1**, as relevant key values including Faradaic and energy efficiencies, molar production rates and single-pass-conversions experienced no significant change, despite increasing the silver­(I) content from 14.3% to 22.8%. Considering synthesis, characterization, and electrochemical data, complex **Ag­(TPT-1)**_**2**_ proved to be the most stable and selective catalyst for the CO_2_-to-CO conversion, achieving Faradaic efficiencies of around 80%, energy efficiencies of 24%, and single-pass conversions of 19% at a practically relevant current density of 200 mA cm^–2^ in our custom-built CO_2_ electrolyzer cell.

The **Ag­(TPT-1)**_**2**_ molecular complex and **Ag**_**2**_ **(TPT-1)**_**2**_ polymeric gel achieve higher Faradaic efficiencies than Ag nanoparticles in CO_2_-to-CO electroreduction, delivering higher activity at lower loadings. Their performance is proposed to originate from the noninnocent **TPT-1** ligand, which can facilitate ligand-centered reduction processes and can contribute to CO_2_ activation, demonstrating the advantage of ligand-engineered silver catalysts in gas diffusion systems.

## Supplementary Material



## Notes

Additional experimental data (synthesis, electrochemical results) on ligand and complexes (PDF)
