# Supplementary material for: Molecularly Engineered Silver(I) Gel Catalysts for Industrial-Level CO2 Electroreduction
Source: Inorg Chem. 2026 Jun 26;65(27):15675–84. doi: 10.1021/acs.inorgchem.6c01721 (PMC13370877; doi:10.1021/acs.inorgchem.6c01721)
Supplement: Supplementary file 1 [file ic6c01721_si_001.pdf]

Molecularly Engineered Silver(I) Gel Catalysts for Industrial-Level CO_2 Electroreduction

Simon Offenthaler[a], Sankitkumar Vala[a], and Wolfgang Schöfberger*[a]

[a] Institute of Organic Chemistry, Laboratory for Sustainable Chemistry and Catalysis (LSusCat), Johannes Kepler University (JKU), Altenberger Straße 69, 4040 Linz, Austria.

Corresponding Authors: wolfgang.schoefberger@jku.at

Keywords: Triazines • 2,4,6-tri-(1H-pyrazol-1-yl)-1,3,5-triazine • 2,4,6-tri-(2-pyridyl)-1,3,5-triazine • coordination polymer • electrochemical CO_2 reduction • H-type electrochemical cell • zero-gap electrochemical cell.

Table of Contents

1. Experimental

1.1 2,4,6-Tri-(1H-Pyrazol-1-yl)-1,3,5-Triazine 3

1.2 Di-2,4,6-Tri-(1H-Pyrazol-1-yl)-1,3,5-Triazine-Silver(I)-Tetrafluoroborate 10

1.3 Di-2,4,6-Tri-(1H-Pyrazol-1-yl)-1,3,5-Triazine-Silver(I)Silver(I)-Tetrafluoroborate 18

2. Experimental

2.1 Homo- and Heterogeneous Electrochemical Setups 26

2.2 Homo- and Heterogeneous Electrochemical Results 29

2.3 2,4,6-Tri-(1H-Pyrazol-1-yl)-1,3,5-Triazine 30

2.4 Di-2,4,6-Tri-(1H-Pyrazol-1-yl)-1,3,5-Triazine-Silver(I)-Tetrafluoroborate and Di-2,4,6-Tri-(1H-Pyrazol-1-yl)-1,3,5-Triazine-Silver(I)Silver(I)-Tetrafluoroborate 31

1. Experimental

Chemicals and solvents were purchased from Alfa Aesar, BLDpharm, The Fuel Cell Store, Merck, Sigma-Aldrich, Strem Chemicals, Thermo Fisher Scientific and VWR International, and were used without further purification unless otherwise specified. Anhydrous solvents (THF and CH_3CN) were obtained from a molecular sieve under N_2 atmosphere (MB-SPS-7, M. Braun Inertgas-Systeme). High purity water (18 MΩ cm) was received from a Milli-Q® Reference A+ purification system with MillipakQ®-40 filter unit (0.22 µm pore size, Merck). High-purity gases (argon 5.0, carbon dioxide 4.5 and helium 5.0) were sourced from Linde Gas.

^{1}H-NMR and ^{13}C-NMR spectra were recorded on a Bruker AVIII 300 or a Bruker AVIII 500 MHz spectrometer in deuterated solvents: $CDCl_3$, DMSO-d_6 (both Sigma-Aldrich) and D_2O (Eurisotop). Chemical shifts (δ) are reported in parts per million (ppm) referenced to the residual solvent signals for ^{1}H and ^{13}C unless otherwise noted.

UV/Vis absorption spectra were acquired on a Varian Cary 300 Bio UV/Vis spectrophotometer. The measurements were performed in CH_2Cl_2 and CH_3CN using standard 10 mm quartz cuvettes. FT-IR absorption spectra were obtained on a Bruker ALPHA II Compact FT-IR spectrometer.

XPS spectra were measured on a Thermo Fisher Scientific Nexsa G2 Surface Analysis System, equipped with a monochromatic Al Kα source ($h\nu$ = 1486.6 eV). The pressure within the analysis chamber was maintained at ultrahigh vacuum (~10^{-9} mbar). Powder samples were tightly pressed onto indium foil to ensure good electrical contact with the sample holder. XPS survey spectra were recorded over the binding energy range 0–1400 eV with 200 eV pass energy and 30 s dwell time. High-resolution XPS spectra of C 1s, N 1s, F 1s, Ag 3d and AgMNN for chemical state analysis were recorded with 20 eV pass energy and 30 s dwell time. The binding energies were referenced to the C 1s signal at 285 eV to correct for possible charging effects. Quantitative elemental compositions (at.%) were determined from the recorded survey spectra by peak area integration using instrument-specific atomic sensitivity factors. Data analysis, including peak fitting and background subtraction, was performed in Thermo Scientific Avantage Software.

HRMS-ESI spectra were recorded on an Agilent 6520 Q-TOF mass spectrometer with ESI source, Agilent G1607A coaxial sprayer and Thermo Fisher Scientific LTQ Orbitrap XL with Ion Max API source in positive mode. Samples were prepared as particle-free solutions in CH_2Cl_2 or CH_3CN.

1.1 2,4,6-Tri-(1H-Pyrazol-1-yl)-1,3,5-Triazine

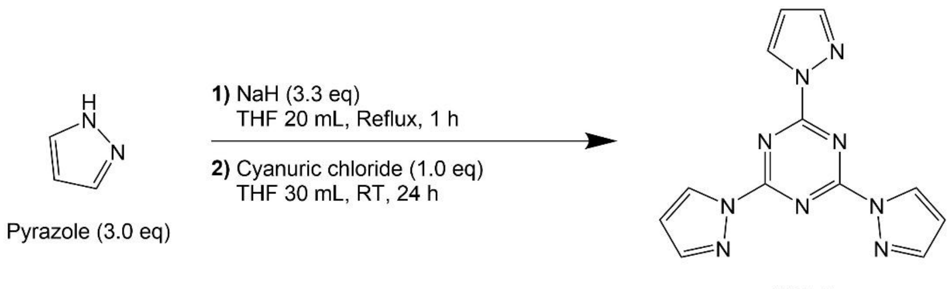

Scheme S1. Heteroaryl nucleophilic substitution of Cl on a 1,3,5-triazine ring by pyrazolate to obtain ligand **TPT-1**.

NaH (284 mg, 11.8 mmol, 3.3 eq) was dispersed in dry THF (20 mL) under a N_2 atmosphere, and pyrazole (731 mg, 10.7 mmol, 3.0 eq) was added portionwise. The mixture was stirred at room temperature until hydrogen evolution ceased, then heated at reflux for 1 h. Upon cooling to room temperature, a solution of cyanuric chloride (660 mg, 3.58 mmol, 1.0 eq) in dry THF (10 mL) was added dropwise. The mixture was stirred at room temperature for 24 h, then THF was removed by rotary evaporation. The crude product was washed with distilled water (3 × 10 mL), collected by suction filtration, and dried under vacuum for 24 h to obtain ligand **TPT-1** as a light beige powder (720 mg, 2.58 mmol, 72%).

^{1}H-NMR (500 MHz, $CDCl_3$, 298 K) δ/ppm: 6.60 (dd, J^1 = 2.8 Hz, J^2 = 1.5 Hz, 3 H, ArH), 7.97 (d, J^1 = 0.9 Hz, 3 H, ArH), 8.81 (d, J^1 = 2.8 Hz, 3 H, ArH). ^{1}H-NMR (300 MHz, $(CD_3)_2SO$, 298 K) δ/ppm: 6.76 (dd, J^1 = 2.8 Hz, J^2 = 1.5 Hz, 3 H, ArH), 8.06 (d, J^1 = 0.9 Hz, 3 H, ArH), 8.99 (d, J^1 = 2.7 Hz, 3 H, ArH). ^{13}C-NMR (126 MHz, $CDCl_3$, 298 K) δ/ppm: 110.6, 130.9, 146.3, 163.9. UV/Vis (CH_2Cl_2 and CH_3CN, 298 K) λ_{max}/nm: CH_2Cl_2: 268 and CH_3CN: 265. FT-IR (ATR, 298 K) ν/cm^{-1}: 3147, 3108, 1571, 1527, 1511, 1437, 1385, 1309, 1297, 1270, 1254, 1223, 1202, 1184, 1147, 1087, 1077, 1050, 1033, 941, 908, 867, 801, 772, 753, 644, 599. XPS (Al Kα, 1486.6 eV, calibrated to C 1s = 285 eV): C 1s (285 eV, 62.3%), N 1s (400 eV, 37.7%). HRMS-ESI (positive mode, t_R = 0.263 min): m/z calculated for ligand $C_{12}H_{10}N_9^+$ = 280.1054 [M+H]$^+$, m/z measured = 280.1089 [M+H]$^+$.

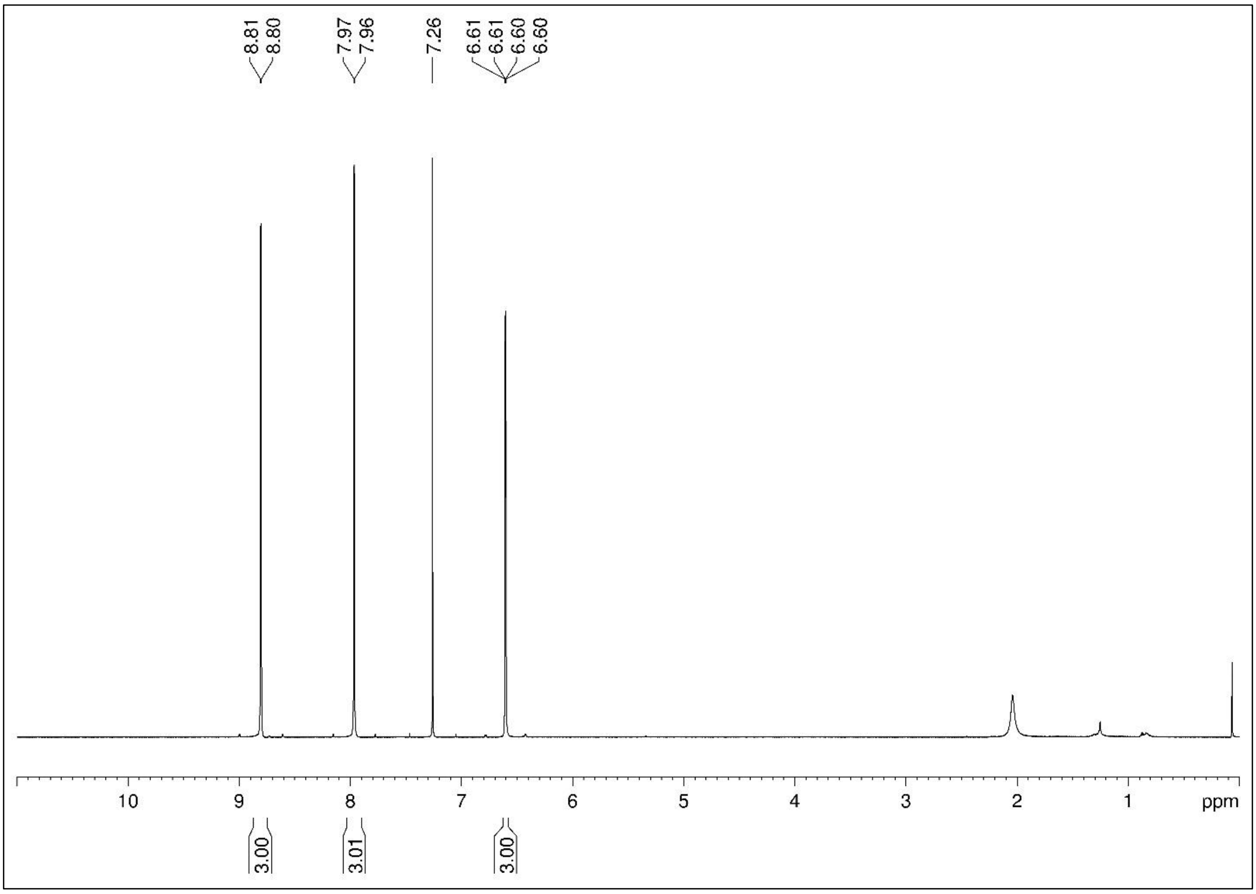

Figure S1. ^{1}H-NMR spectrum (500 MHz, $CDCl_3$, 298 K) of ligand **TPT-1**.

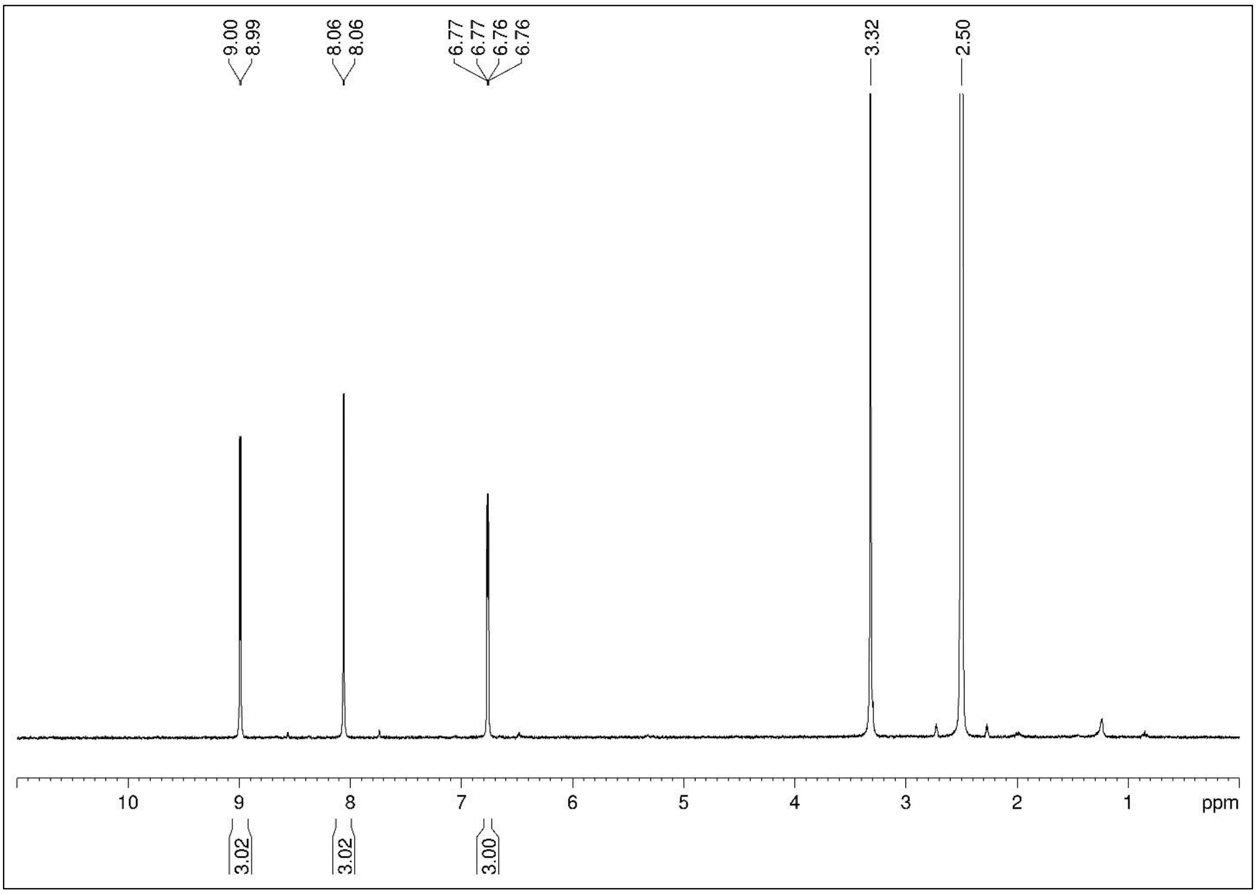

Figure S2. ^{1}H-NMR spectrum (300 MHz, $(CD_3)_2SO$, 298 K) of ligand **TPT-1**.

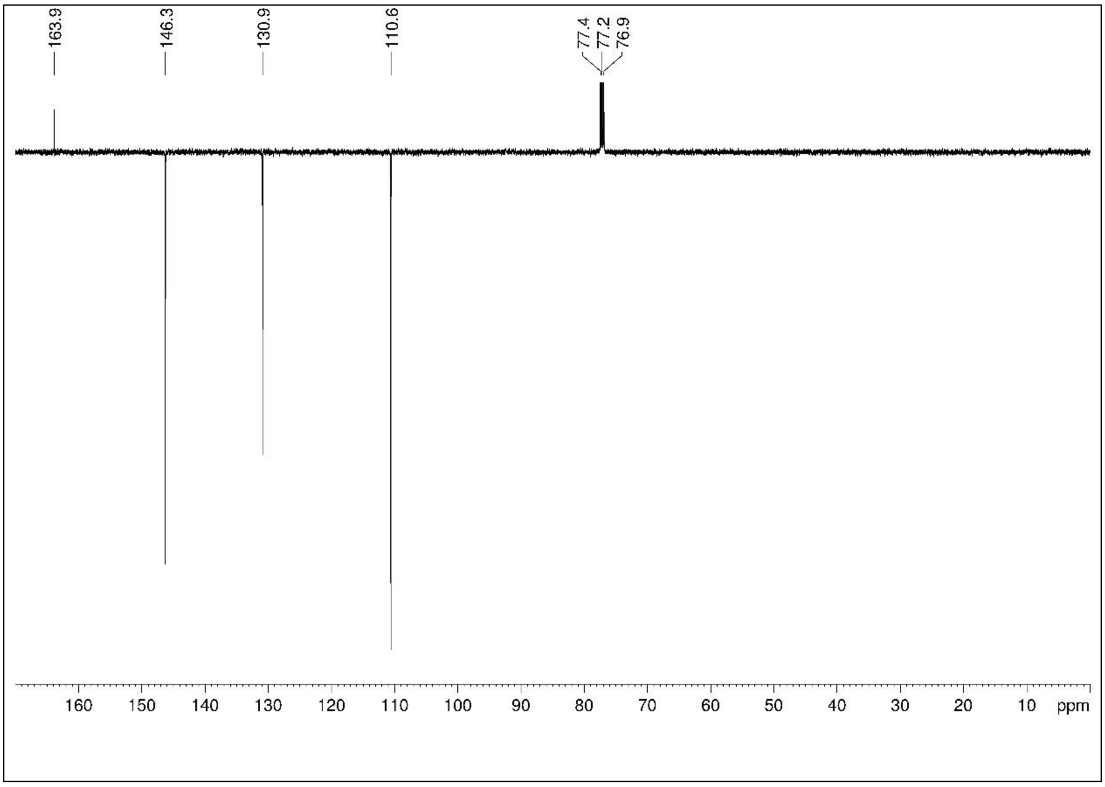

Figure S3. ^{13}C-NMR spectrum (126 MHz, $CDCl_3$, 298 K) of ligand **TPT-1**.

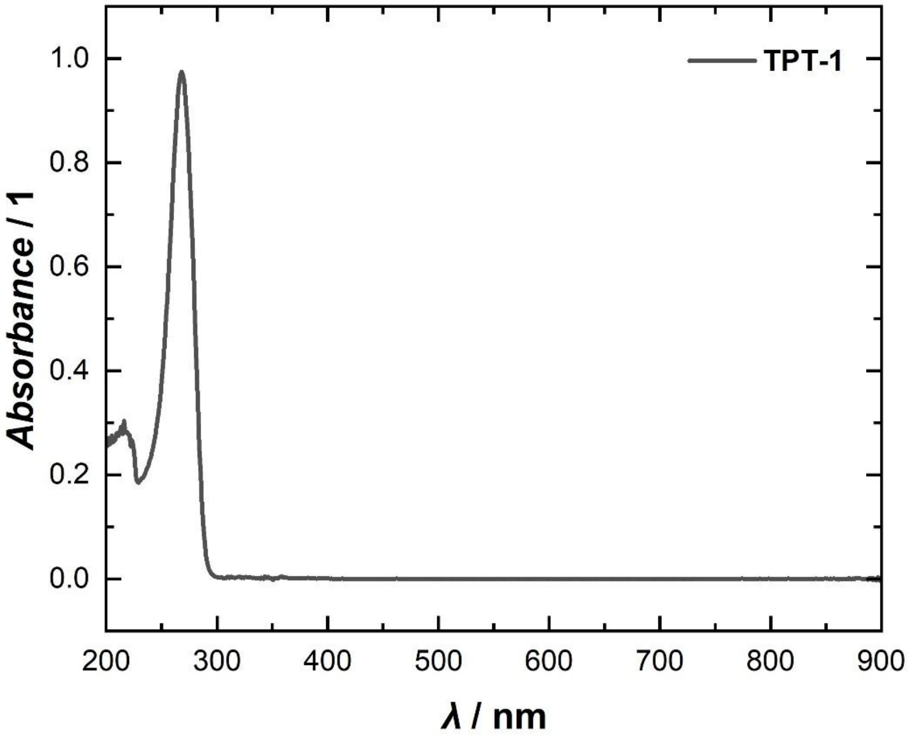

Figure S4. UV/Vis absorption spectrum of ligand **TPT-1** in CH_2Cl_2 with a maximum at 268 nm.

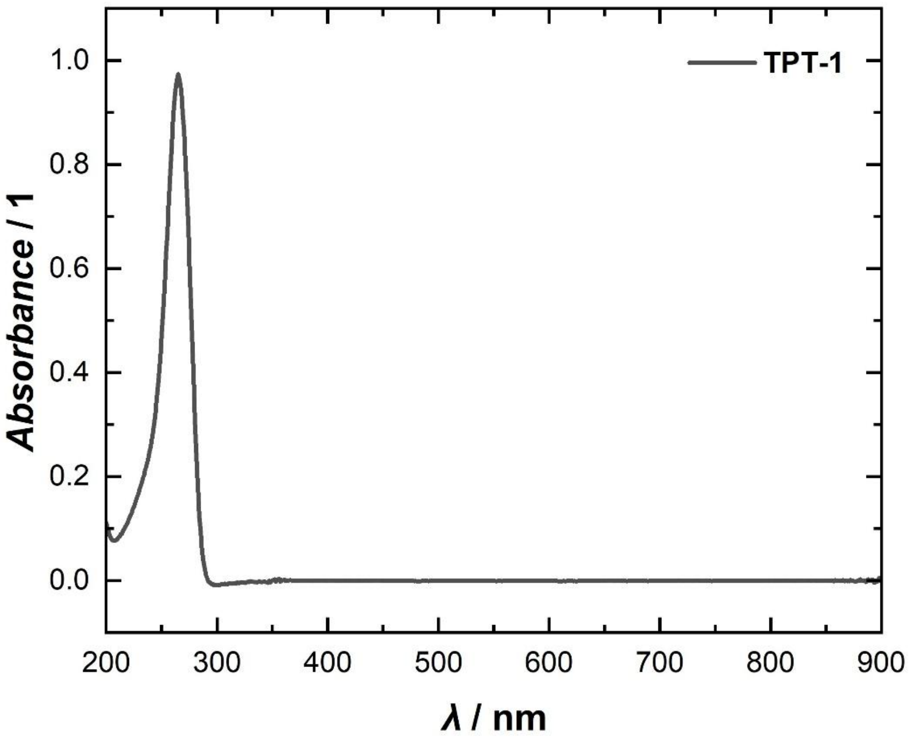

Figure S5. UV/Vis absorption spectrum of ligand **TPT-1** in CH_3CN with a maximum at 265 nm.

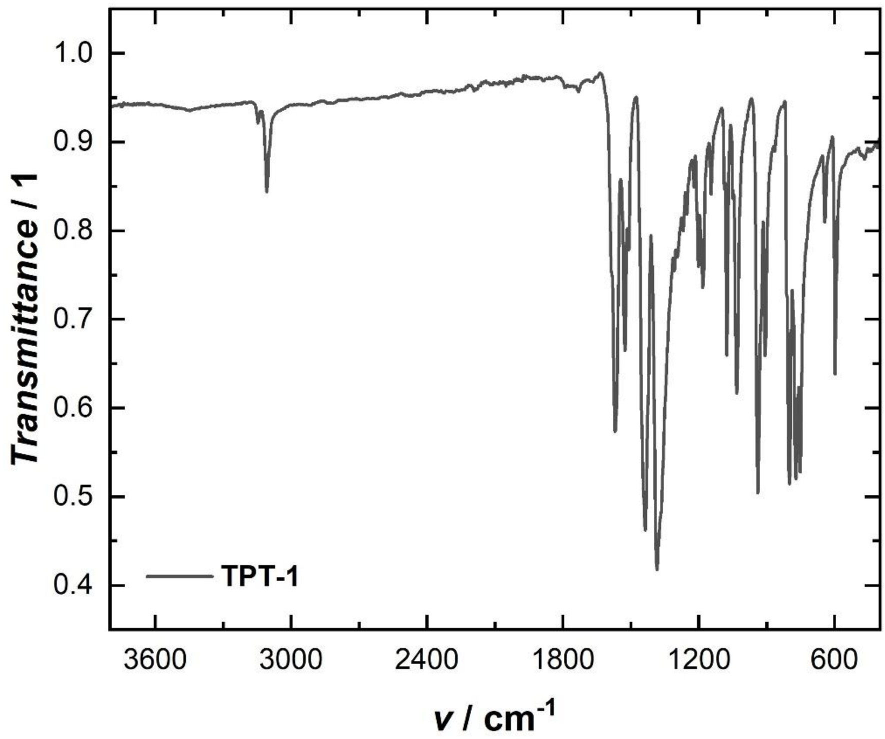

Figure S6. FT-IR absorption spectrum of ligand **TPT-1** recorded by attenuated total reflection (ATR).

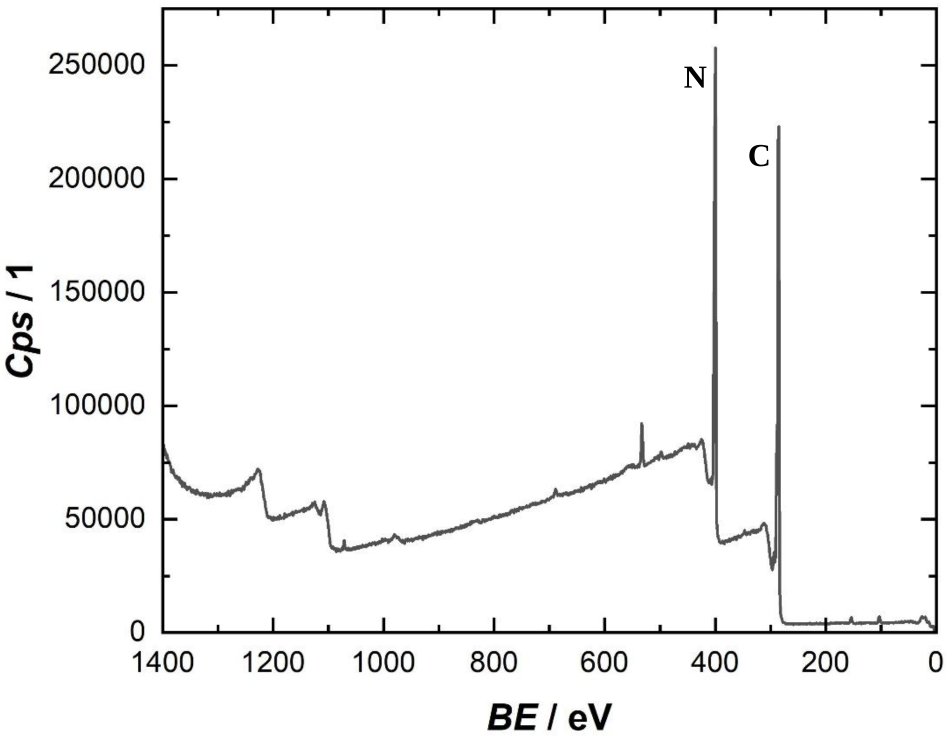

Figure S7. XPS survey spectrum of ligand **TPT-1** recorded over the range 0–1400 eV. The carbon C 1s peak at 285 eV was used as the reference for binding energy calibration.

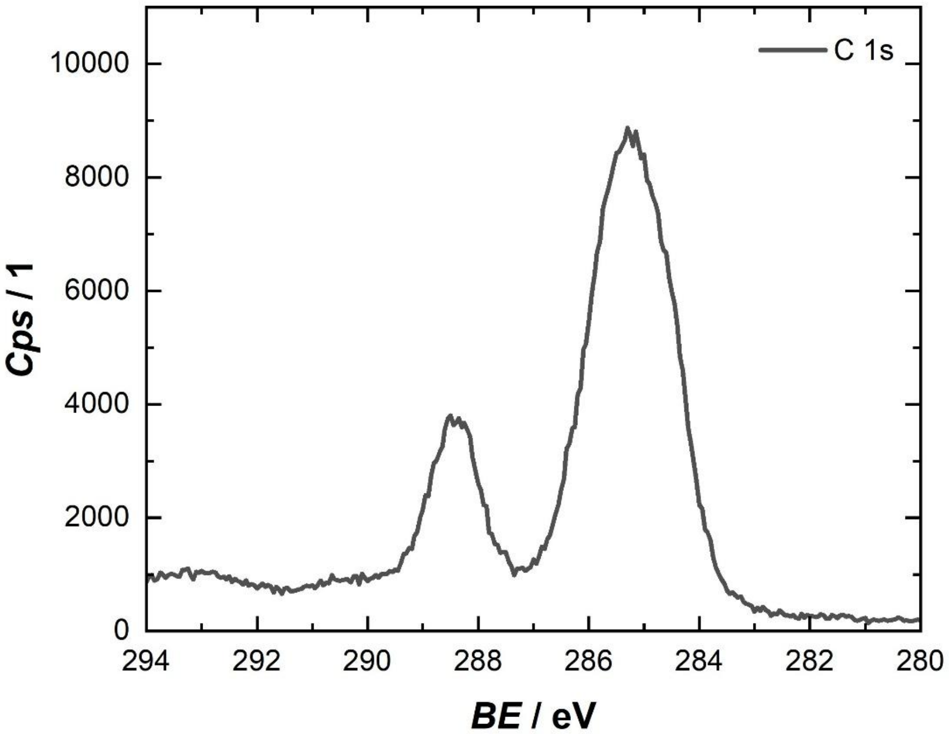

Figure S8. High resolution XPS spectrum of ligand **TPT-1** for C 1s recorded in the range 280–294 eV.

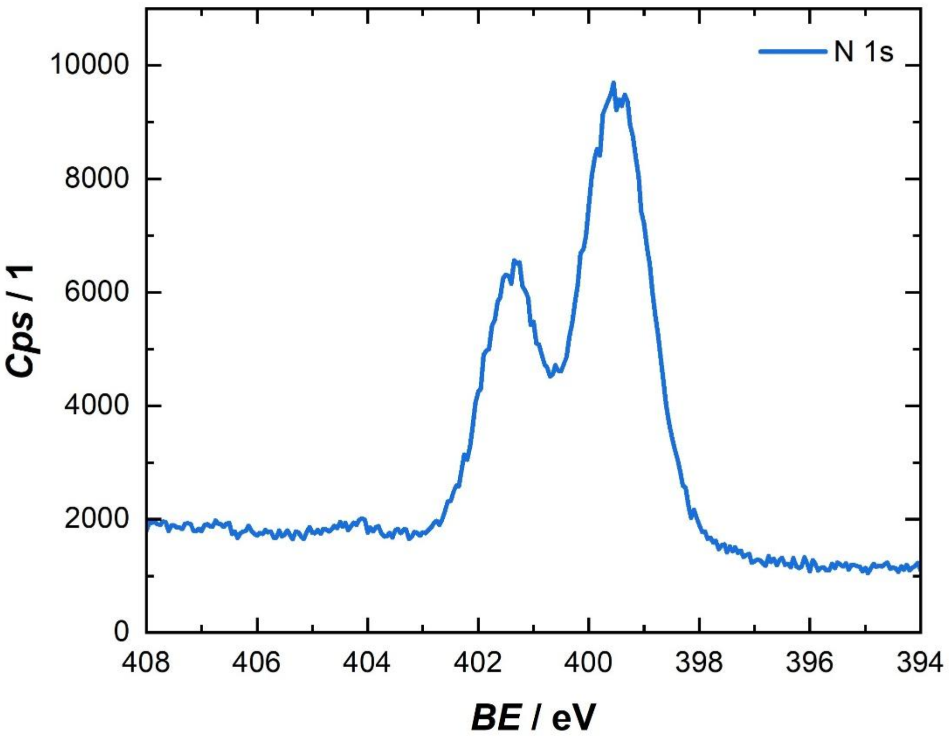

Figure S9. High resolution XPS spectrum of ligand **TPT-1** for N 1s recorded in the range 394–408 eV.

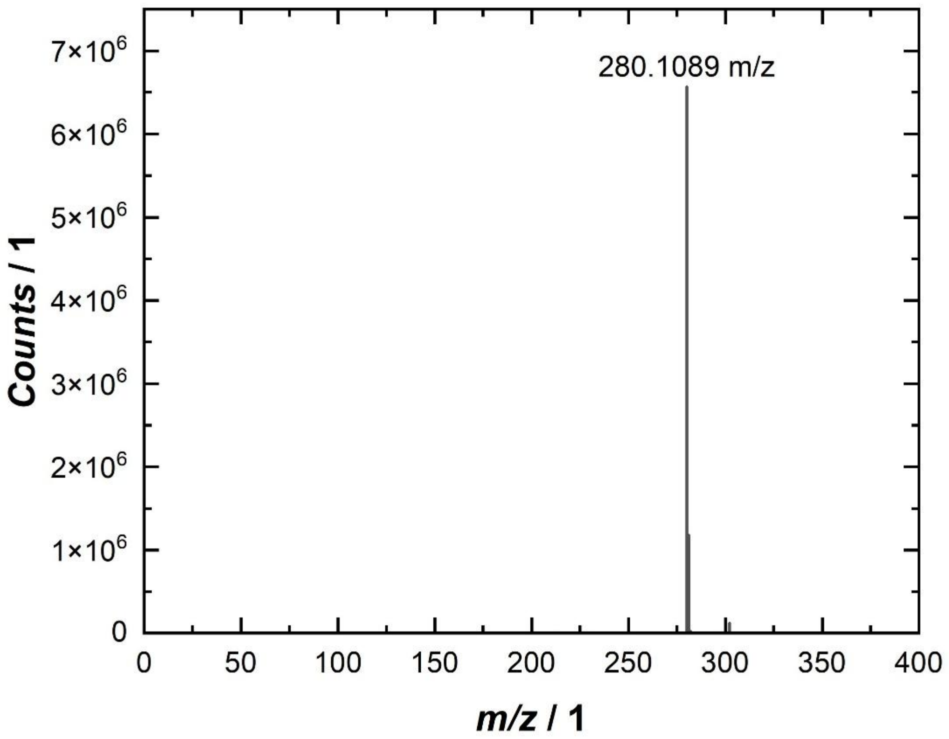

Figure S10. HRMS-ESI spectrum of ligand **TPT-1** with $[M+H]^+$ at 280.1089 m/z (t_R = 0.263 min).

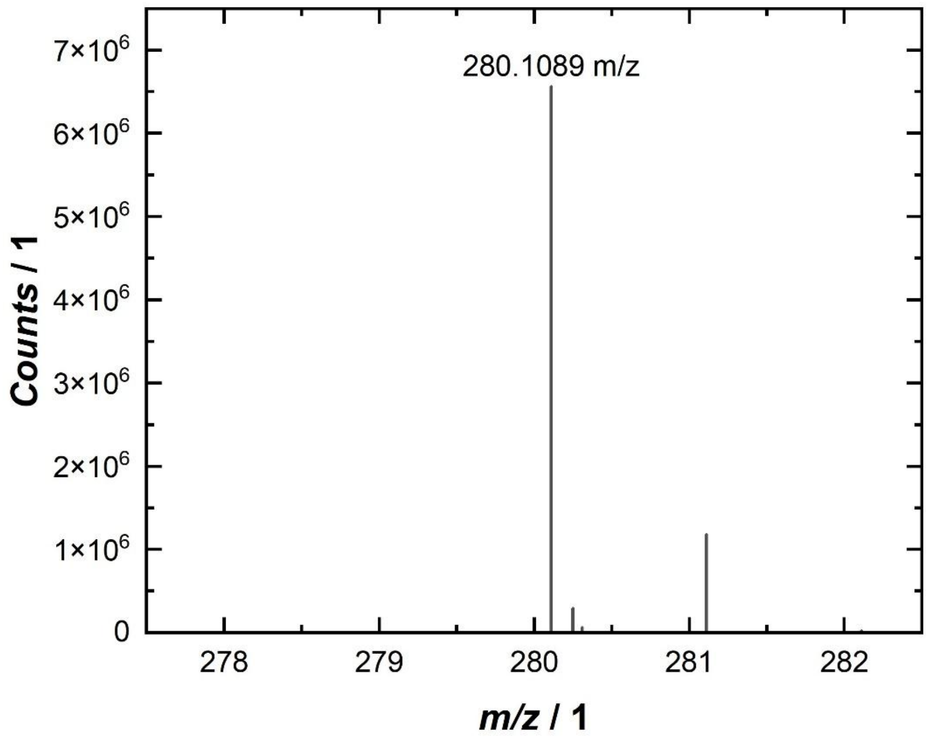

Figure S11. Zoomed-in HRMS-ESI spectrum of ligand **TPT-1** with $[M+H]^+$ at 280.1089 m/z (t_R = 0.263 min).

1.2 Di-2,4,6-Tri-(1H-Pyrazol-1-yl)-1,3,5-Triazine-Silver(I)-Tetrafluoroborate

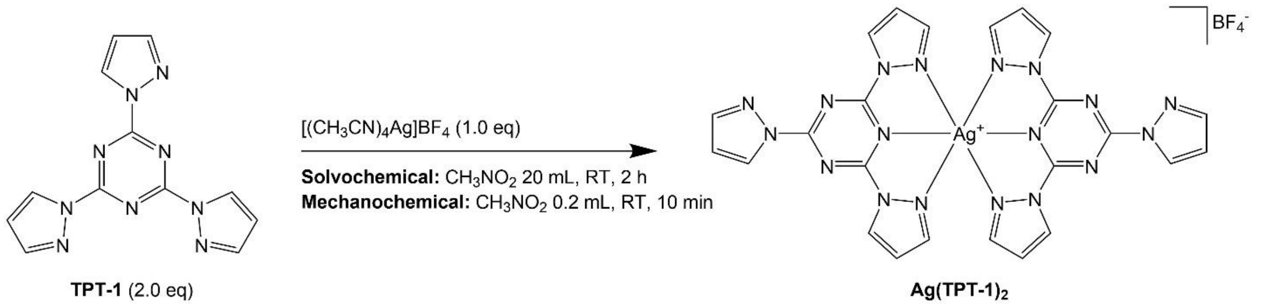

Scheme S2. Solvo- and mechanochemical synthesis to obtain complex **Ag(TPT-1)2**.

<u>Solvochemical synthesis:</u>

A solution of $[(CH_3CN)_4Ag]BF_4$ (95 mg, 0.27 mmol, 1.0 eq) in CH_3NO_2 (10 mL) was added slowly to a solution of **TPT-1** (148 mg, 0.53 mmol, 2.0 eq) in CH_3NO_2 (10 mL). The mixture was stirred at room temperature for 2 h under a N_2 atmosphere. The solvent was removed by rotary evaporation, and the crude product was redissolved in a minimum volume of CH_3NO_2 before being precipitated into Et_2O (50 mL). The product was collected by suction filtration, washed again with Et_2O (3 × 10 mL), and dried under vacuum for 24 h to afford complex **Ag(TPT-1)2** as a light beige powder (150 mg, 0.20 mmol, 75%).

<u>Mechanochemical synthesis:</u>

TPT-1 (148 mg, 0.53 mmol, 2.0 eq), $[(CH_3CN)_4Ag]BF_4$ (95 mg, 0.27 mmol, 1.0 eq) and CH_3NO_2 (0.2 mL) were placed in a 4 mL glass milling vessel together with one 5 mm stainless-steel ball. The mixture was milled at room temperature for 10 min at 3000 rpm. The solvent was removed, the crude product washed with Et_2O (3 × 10 mL), collected by suction filtration, and dried under vacuum for 24 h to afford complex **Ag(TPT-1)2** as a light beige powder (186 mg, 0.25 mmol, 93%).

^{1}H-NMR (500 MHz, $CDCl_3$, 298 K) δ/ppm: 6.63 (dd, J^1 = 1.5 Hz, 1 H, ArH), 7.94 (s, 1 H, ArH), 8.82 (d, J^1 = 2.8 Hz, 1 H, ArH). ^{1}H-NMR (300 MHz, $(CD_3)_2SO$, 298 K) δ/ppm: 6.81 (s, 1 H, ArH), 8.10 (s, 1 H, ArH), 9.06 (d, J^1 = 2.3 Hz, 1 H, ArH). UV/Vis (CH_2Cl_2 and CH_3CN, 298 K) λ_{max}/nm: CH_2Cl_2: 269 and CH_3CN: 265. FT-IR (ATR, 298 K) ν/cm^{-1}: 3565, 3481, 3143, 2832, 1741, 1696, 1675, 1643, 1575, 1529, 1447, 1389, 1270, 1249, 1229, 1212, 1192, 1147, 1062, 1029, 951, 939, 908, 805, 770, 700, 655, 636, 595, 554, 519, 498, 482, 463, 443, 430, 422, 406. XPS (Al Kα, 1486.6 eV, calibrated to C 1s = 285 eV): C 1s (285 eV, 51.0%), N 1s (400 eV, 36.5%), F 1s (685 eV, 9.8%), Ag 3d (368 eV, 2.8%). HRMS-ESI (positive mode, t_R = 0.253 min): m/z calculated for complex $C_{24}H_{18}AgN_{18}^+$ = 665.1008 $[M]^+$, m/z measured = 665.1025 $[M]^+$.

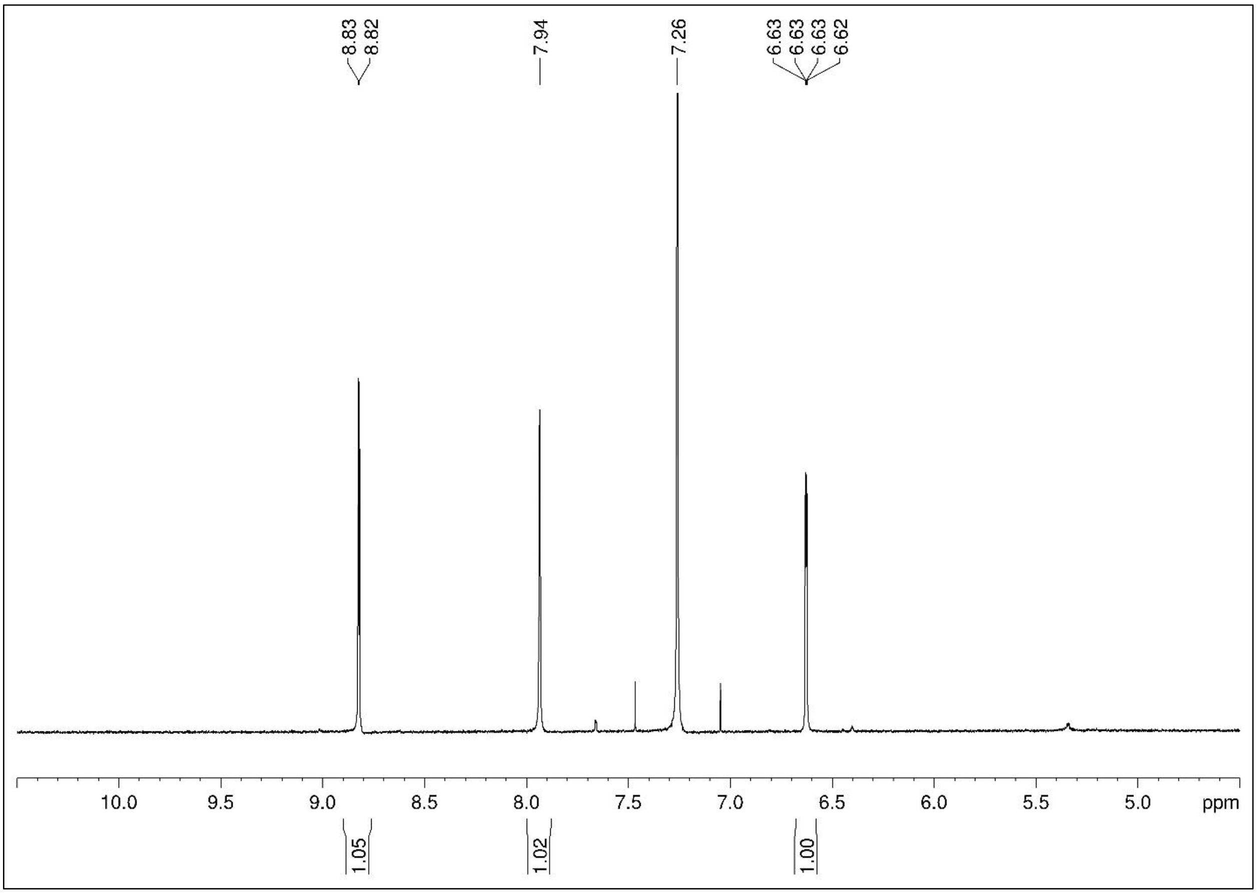

Figure S12. ^{1}H-NMR spectrum (500 MHz, $CDCl_3$, 298 K) of complex **Ag(TPT-1)$_2$**.

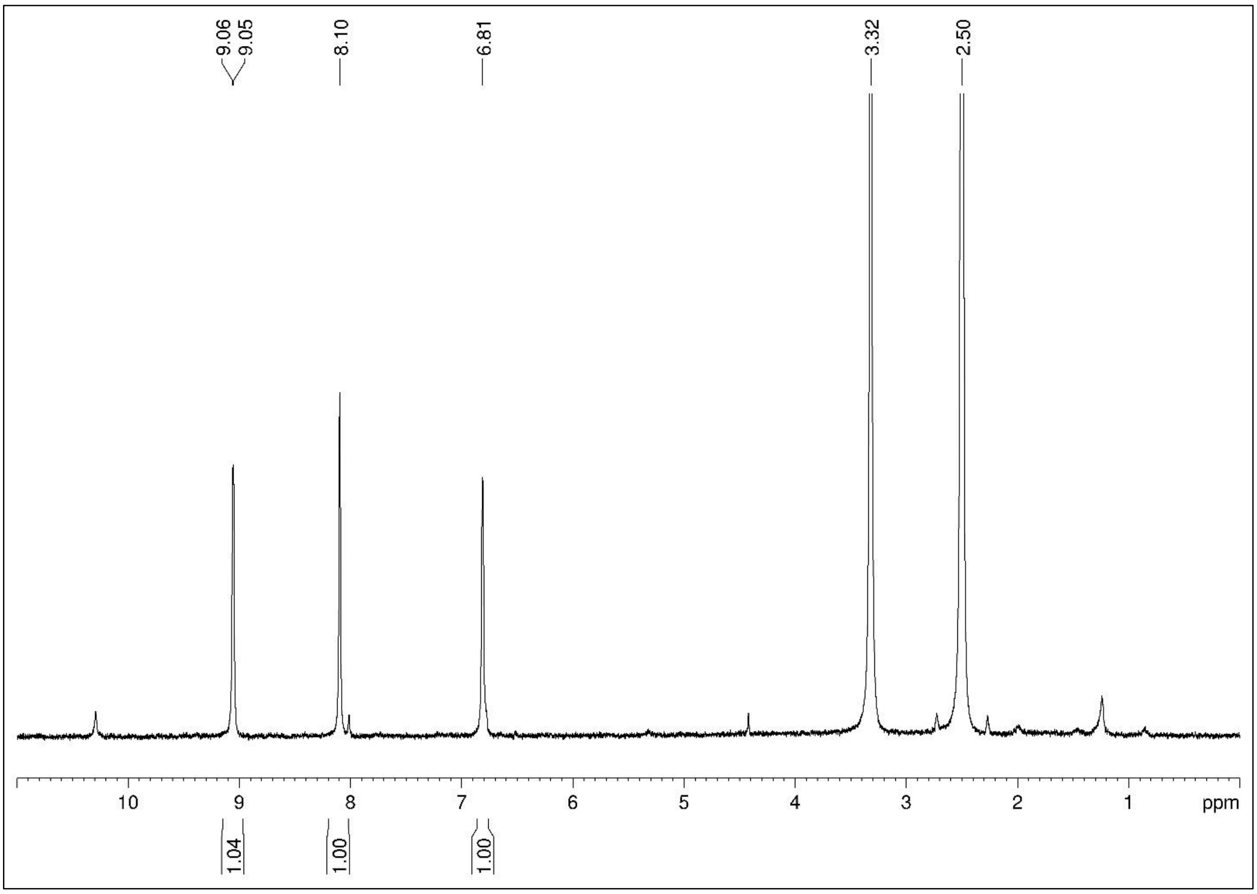

Figure S13. ^{1}H-NMR spectrum (300 MHz, $(CD_3)_2SO$, 298 K) of complex **Ag(TPT-1)$_2$**.

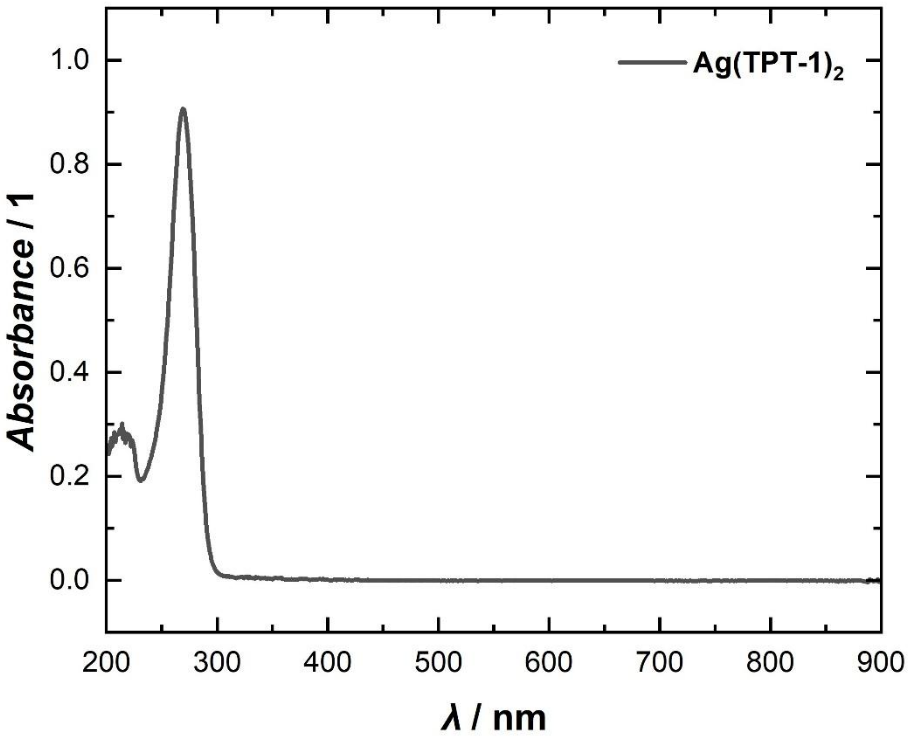

Figure S14. UV/Vis absorbance spectrum of complex **Ag(TPT-1)$_2$** in CH_2Cl_2 with a maximum at 269 nm.

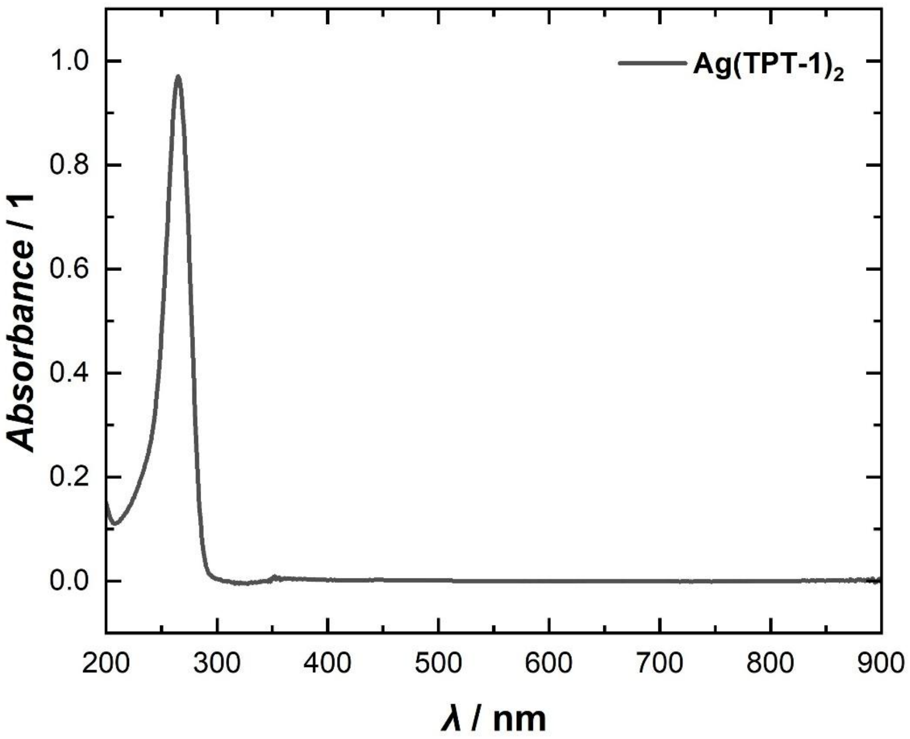

Figure S15. UV/Vis absorbance spectrum of complex **Ag(TPT-1)$_2$** in CH_3CN with a maximum at 265 nm.

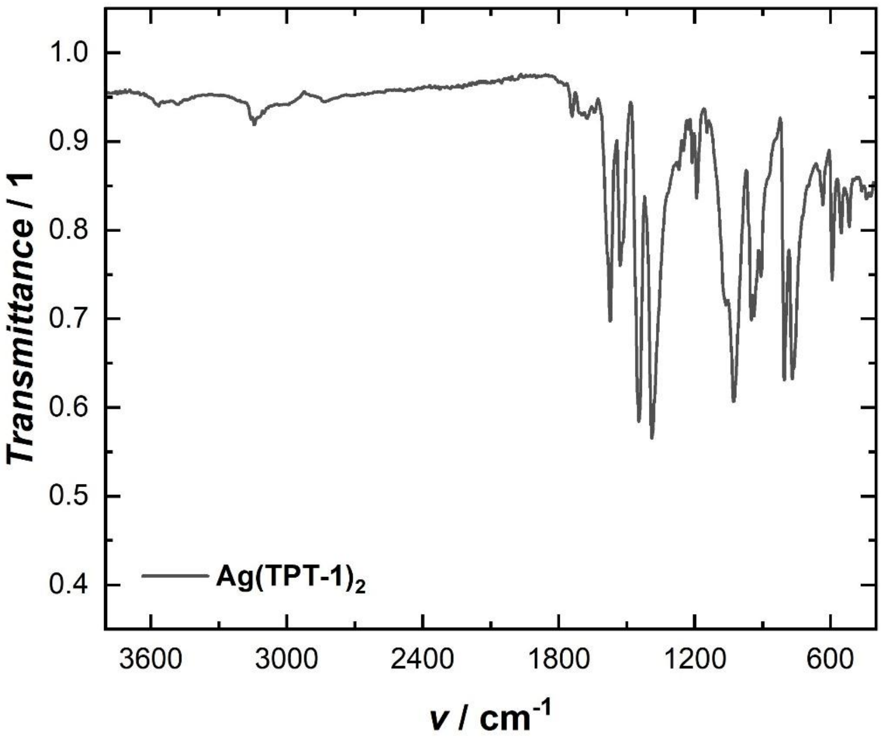

Figure S16. FT-IR absorption spectrum of complex **Ag(TPT-1)$_2$** recorded by attenuated total reflection (ATR).

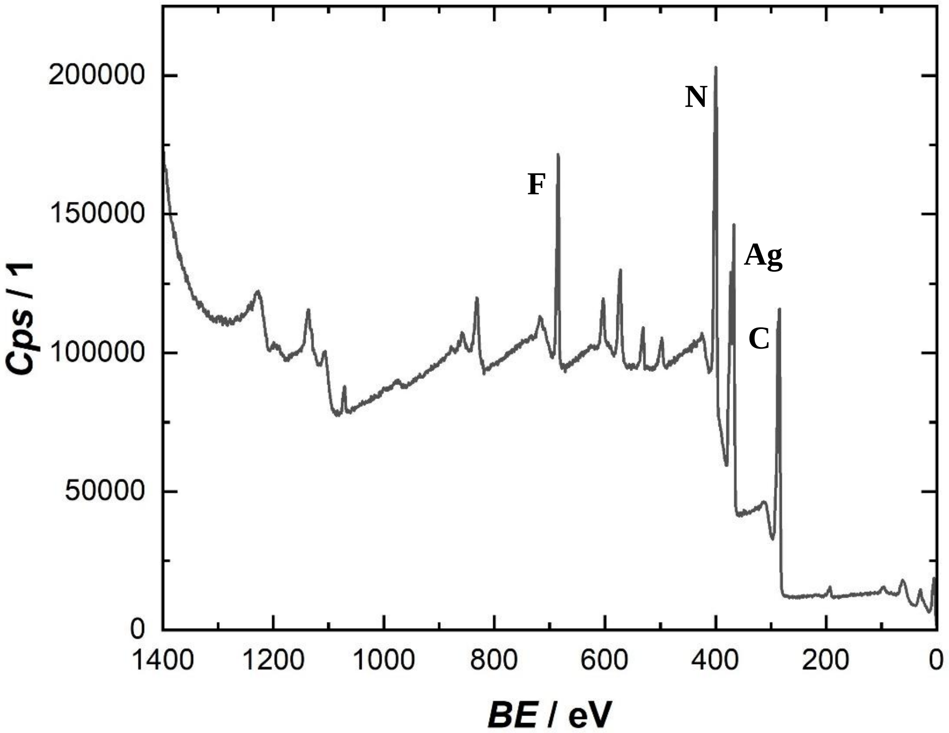

Figure S17. XPS survey spectrum of complex **Ag(TPT-1)$_2$** recorded over the range 0–1400 eV. The carbon C 1s peak at 285 eV was used as the reference for binding energy calibration.

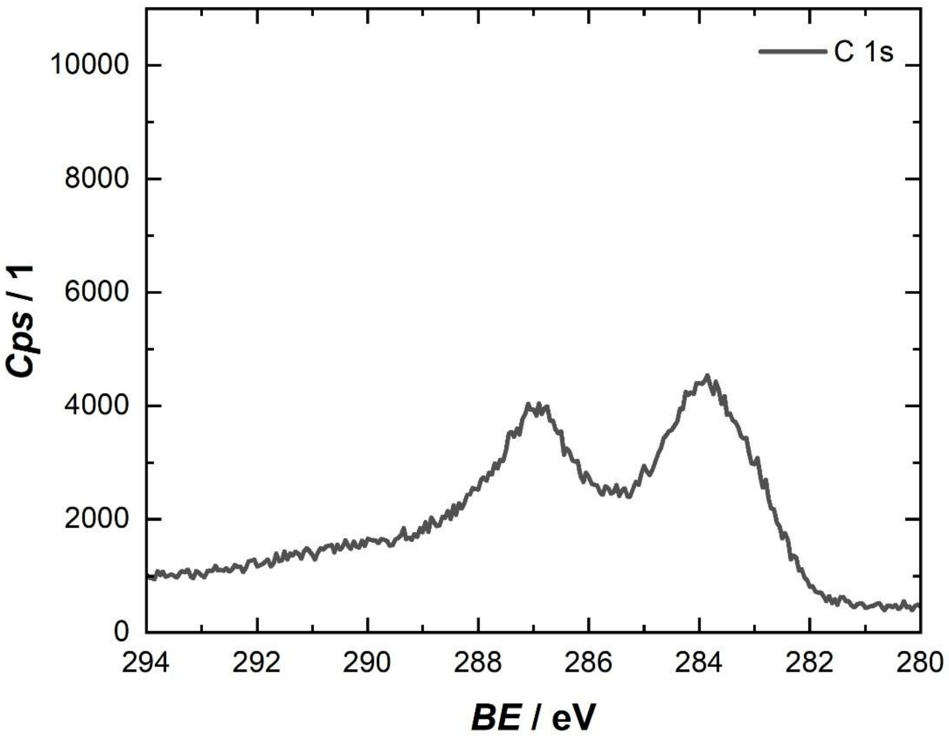

Figure S18. High resolution XPS spectrum of complex **Ag(TPT-1)₂** for C 1s recorded in the range 280–294 eV.

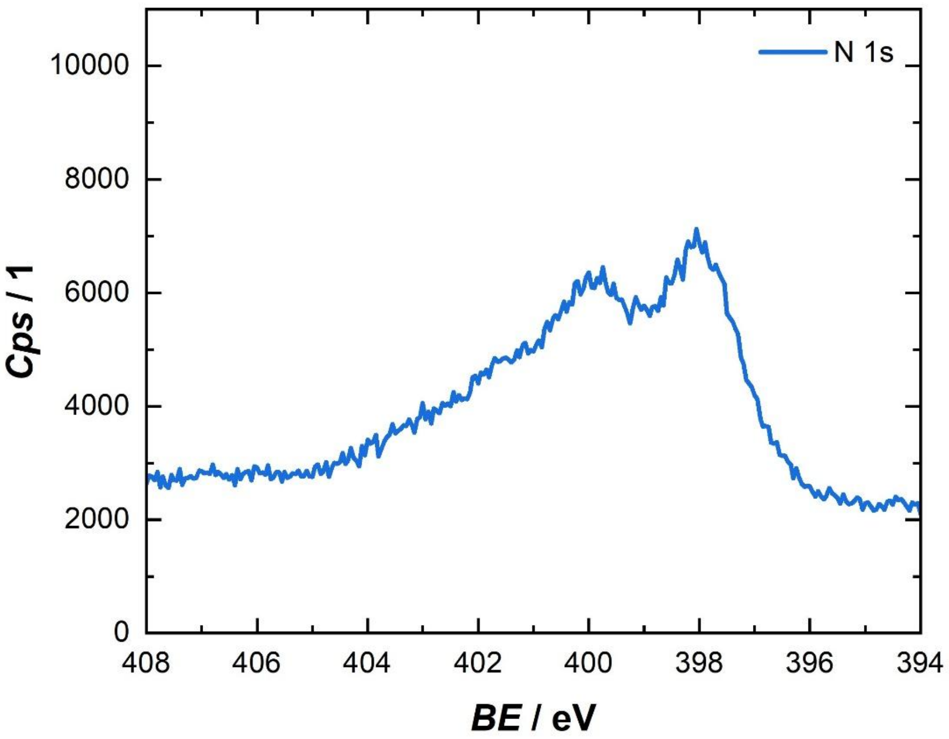

Figure S19. High resolution XPS spectrum of complex **Ag(TPT-1)$_2$** for N 1s recorded in the range 394–408 eV.

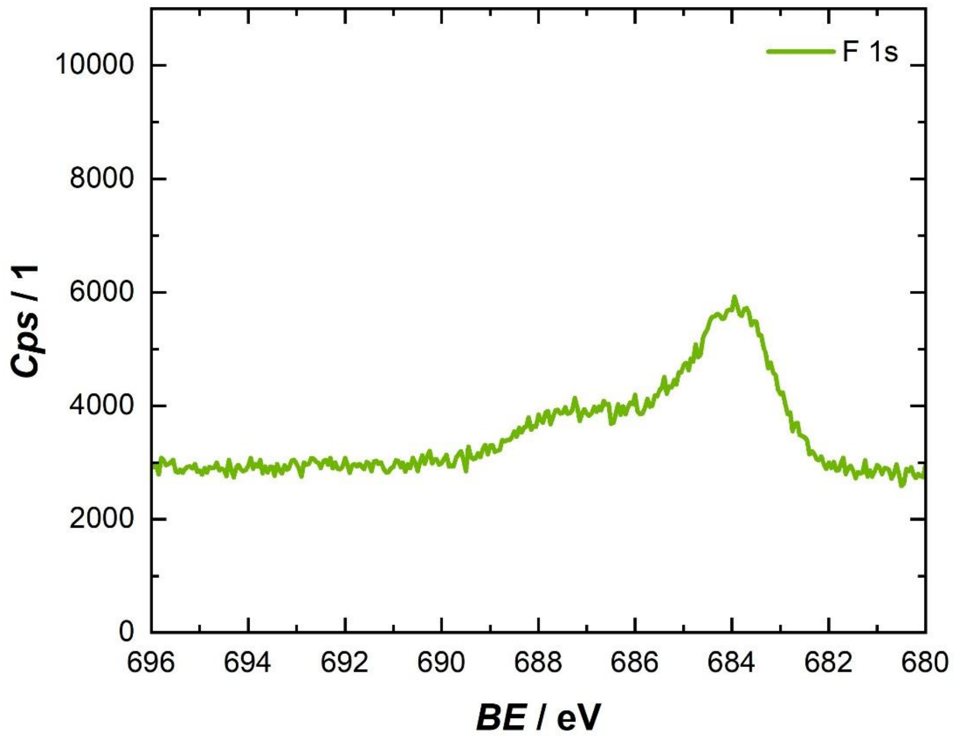

Figure S20. High resolution XPS spectrum of complex **Ag(TPT-1)$_2$** for F 1s recorded in the range 680–696 eV.

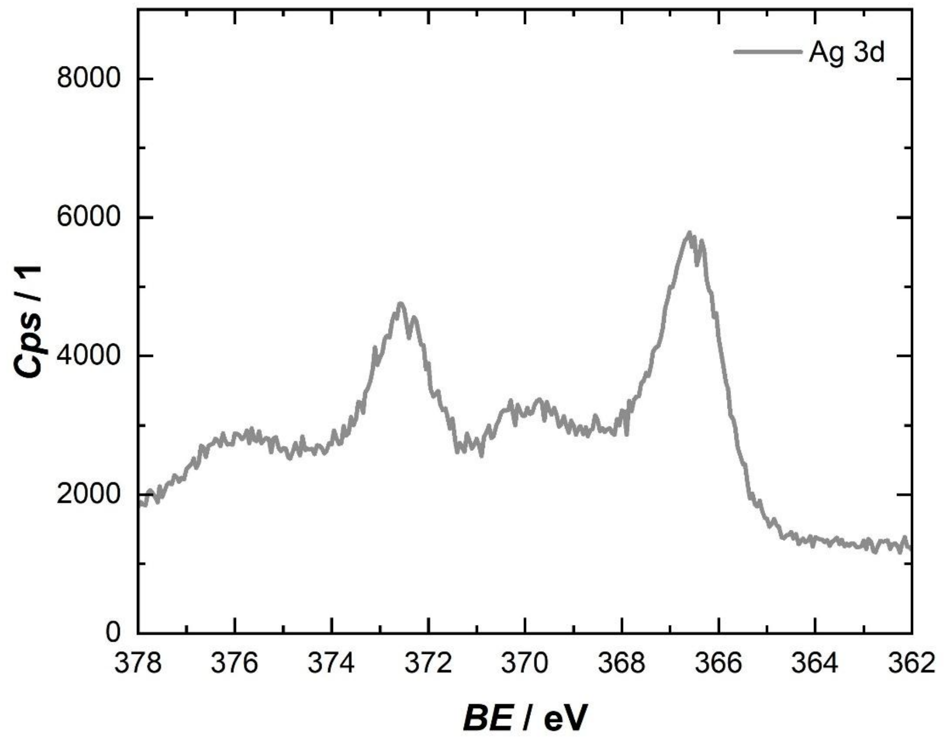

Figure S21. High resolution XPS spectrum of complex **Ag(TPT-1)$_2$** for Ag 3d recorded in the range 362–378 eV.

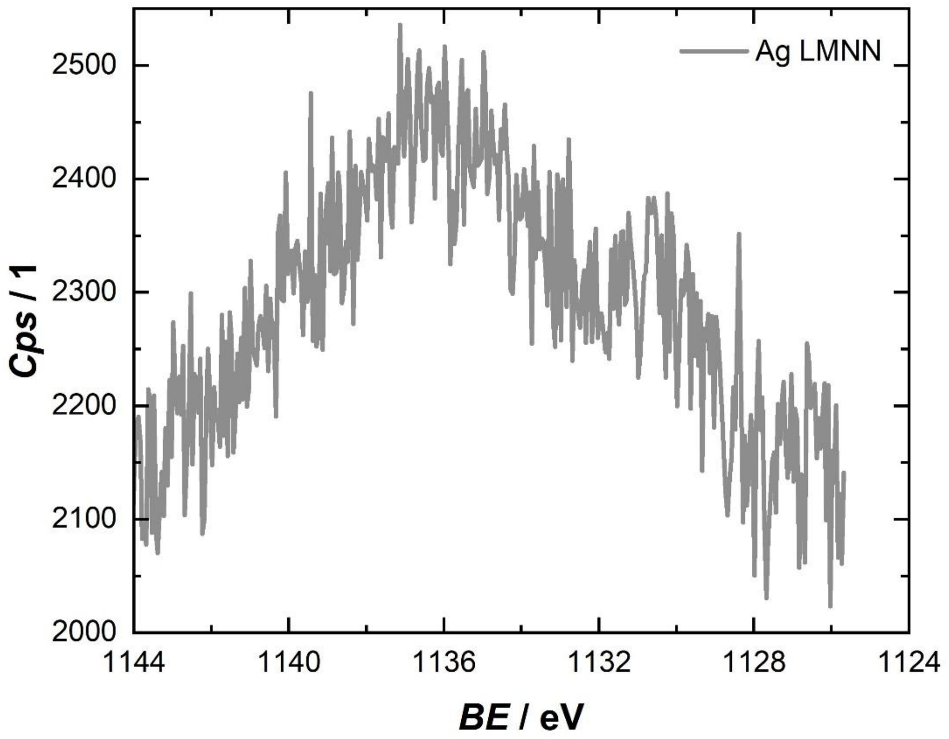

Figure S22. High resolution XPS spectrum of complex **Ag(TPT-1)$_2$** for Ag LMNN recorded in the range 1124–1144 eV.

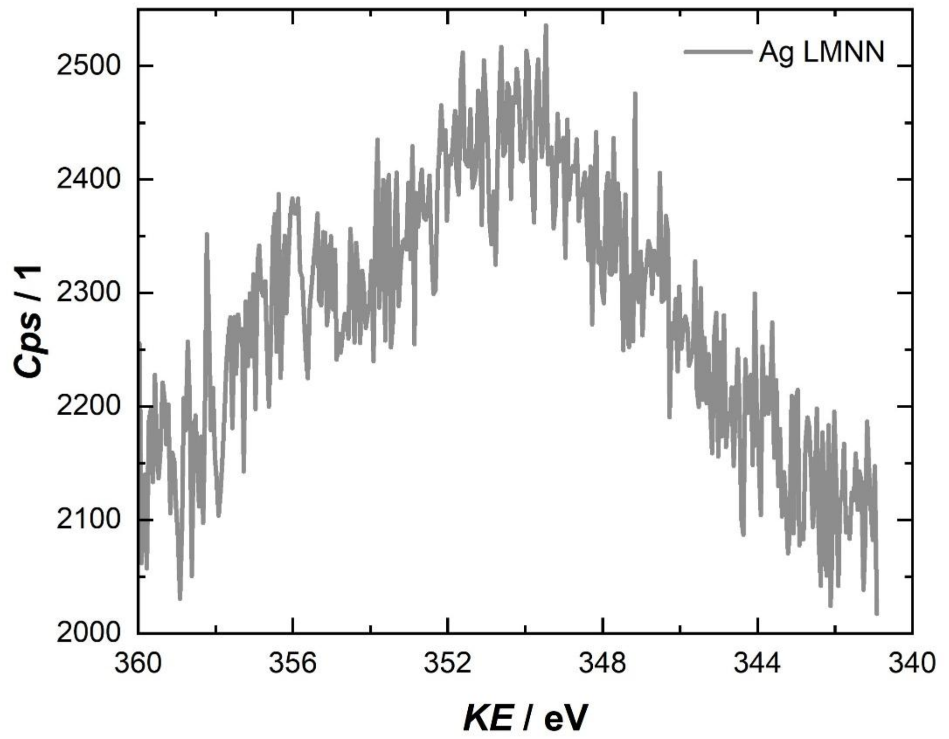

Figure S23. High resolution XPS spectrum of complex **Ag(TPT-1)$_2$** for Ag LMNN recorded in the range 340–360 eV.

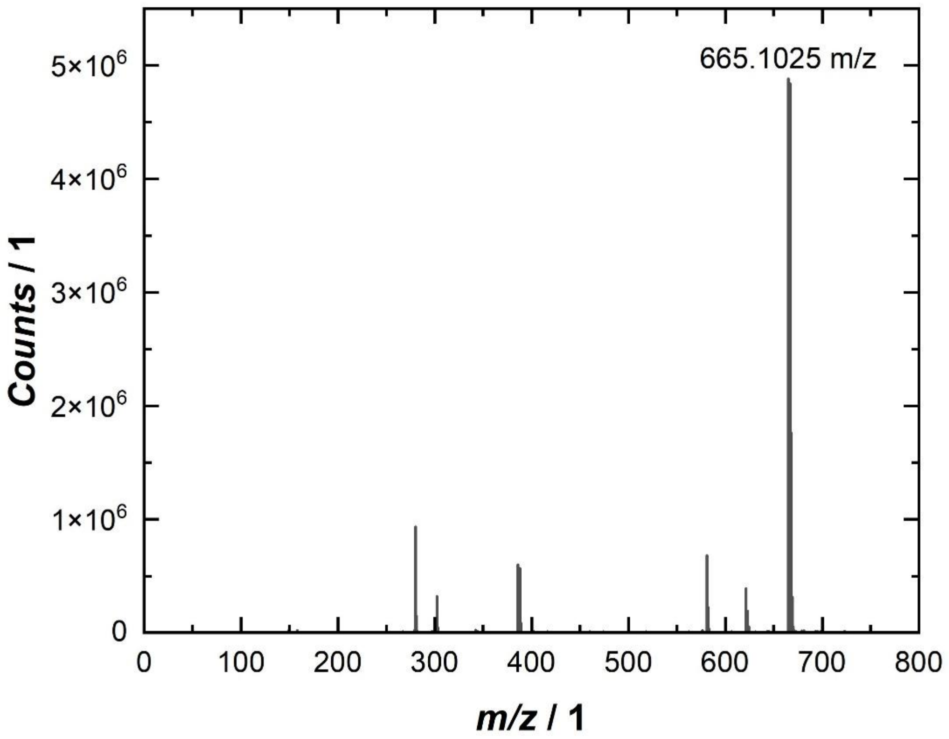

Figure S24. HRMS-ESI spectrum of complex **Ag(TPT-1)$_2$** with $[M]^+$ at 665.1025 m/z (t_R = 0.253 min).

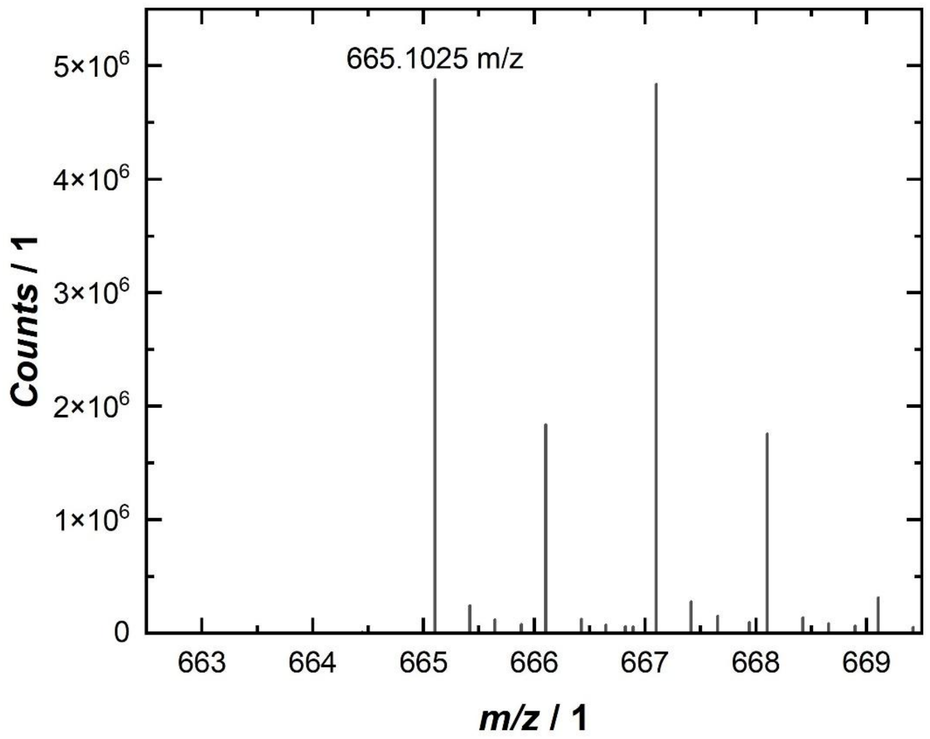

Figure S25. Zoomed-in HRMS-ESI spectrum of complex **Ag(TPT-1)$_2$** with $[M]^+$ at 665.1025 m/z (t_R = 0.253 min).

1.3 Di-2,4,6-Tri-(1H-Pyrazol-1-yl)-1,3,5-Triazine-Silver(I)Silver(I)-Tetrafluoroborate

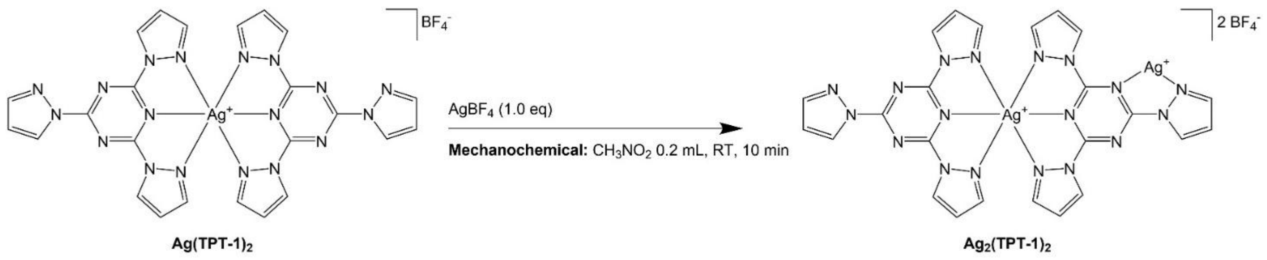

Scheme S3. Mechanochemical synthesis to obtain complex **Ag_2(TPT-1)$_2$**.

<u>Solvochemical synthesis:</u>

No solvochemical synthesis route was used. Complex **Ag_2(TPT-1)$_2$** was exclusively synthesized by mechanochemistry due to the higher yields and reduced solvent consumption, as observed in previous metalations.

<u>Mechanochemical synthesis:</u>

Ag(TPT-1)$_2$ (159 mg, 0.21 mmol, 1.0 eq), $AgBF_4$ (41 mg, 0.21 mmol, 1.0 eq) and CH_3NO_2 (0.2 mL) were placed in a 4 mL glass milling vessel together with one 5 mm stainless-steel ball. The mixture was milled at room temperature for 10 min at 3000 rpm. The solvent was removed, the crude product washed with Et_2O (3 × 10 mL), collected by suction filtration, and dried under vacuum for 24 h to afford complex **Ag_2(TPT-1)$_2$** as a grey powder (187 mg, 0.20 mmol, 94%).

^{1}H-NMR (300 MHz, $(CD_3)_2SO$, 298 K) δ/ppm: 6.86 (s, 1 H, ArH), 8.15 (s, 1 H, ArH), 9.11 (s, 1 H, ArH) UV/Vis (CH_2Cl_2 and CH_3CN, 298 K) λ_{max}/nm: CH_2Cl_2: 272 and CH_3CN: 265. FT-IR (ATR, 298 K) ν/cm^{-1}: 3197, 3157, 3137, 3499, 2357, 2260, 1651, 1638, 1575, 1525, 1515, 1449, 1396, 1365, 1291, 1247, 1196, 1153, 1087, 1044, 965, 935, 914, 883, 807, 797, 780, 766, 727, 661, 636, 628, 595, 545, 523, 494, 476, 451, 422, 414. XPS (Al Kα, 1486.6 eV, calibrated to C 1s = 285 eV): C 1s (285 eV, 37.6%), N 1s (400 eV, 31.1%), F 1s (686 eV, 22.4%), Ag 3d (368 eV, 8.9%). HRMS-ESI (positive mode, t_R = 0.361 min): m/z calculated for complex $C_{24}H_{18}Ag_2N_{18}^+$ = 386.0027 $[M]^{2+}$, m/z measured = 386.0023 $[M]^{2+}$.

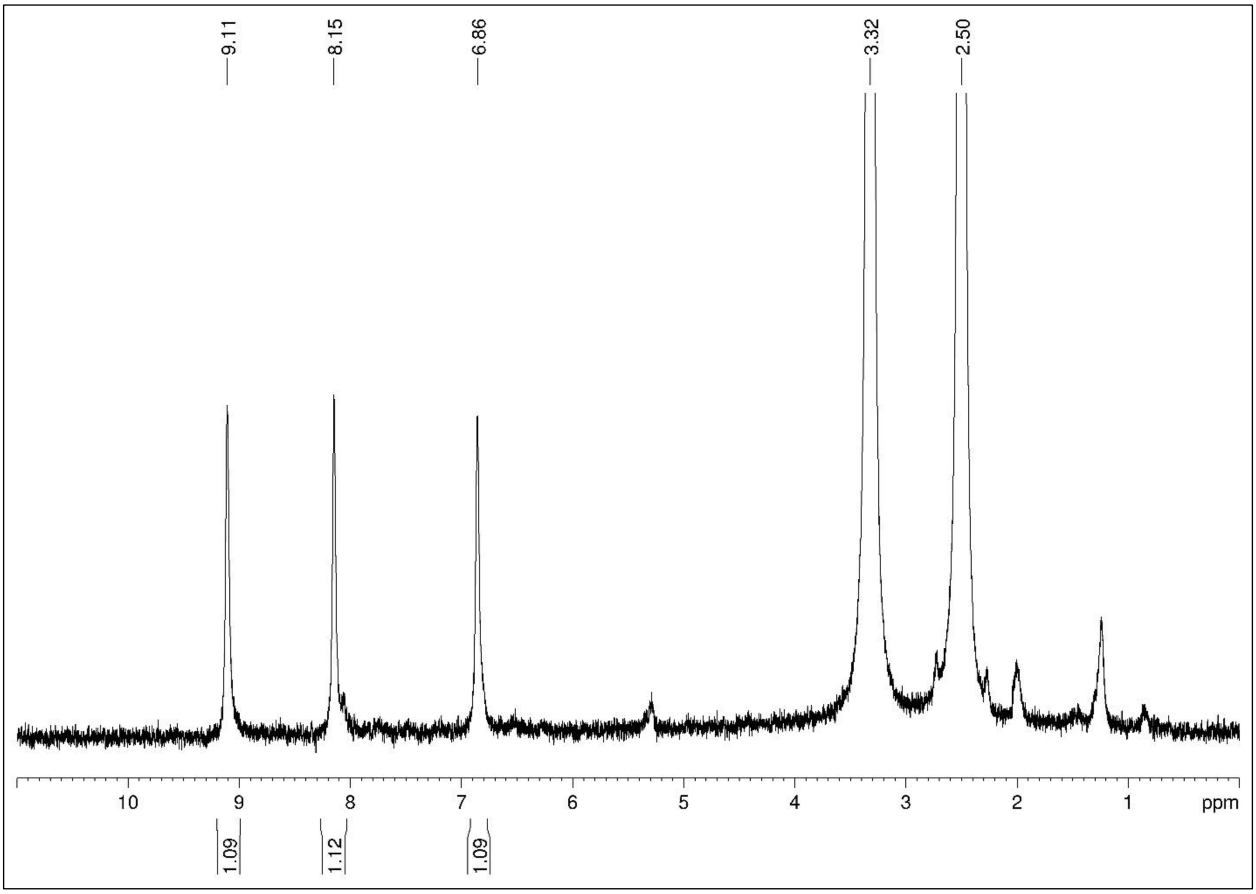

Figure S26. ^{1}H-NMR spectrum (300 MHz, $(CD_3)_2SO$, 298 K) of complex **Ag_2(TPT-1)$_2$**.

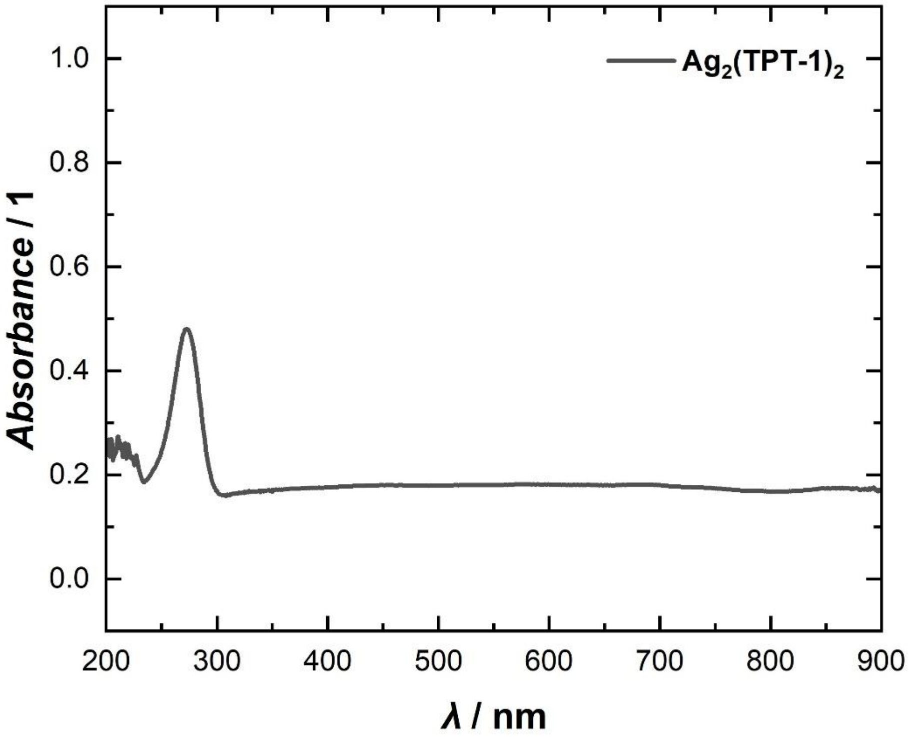

Figure S27. UV/Vis absorption spectrum of complex **Ag_2(TPT-1)$_2$** in CH_2Cl_2 with a maximum at 272 nm.

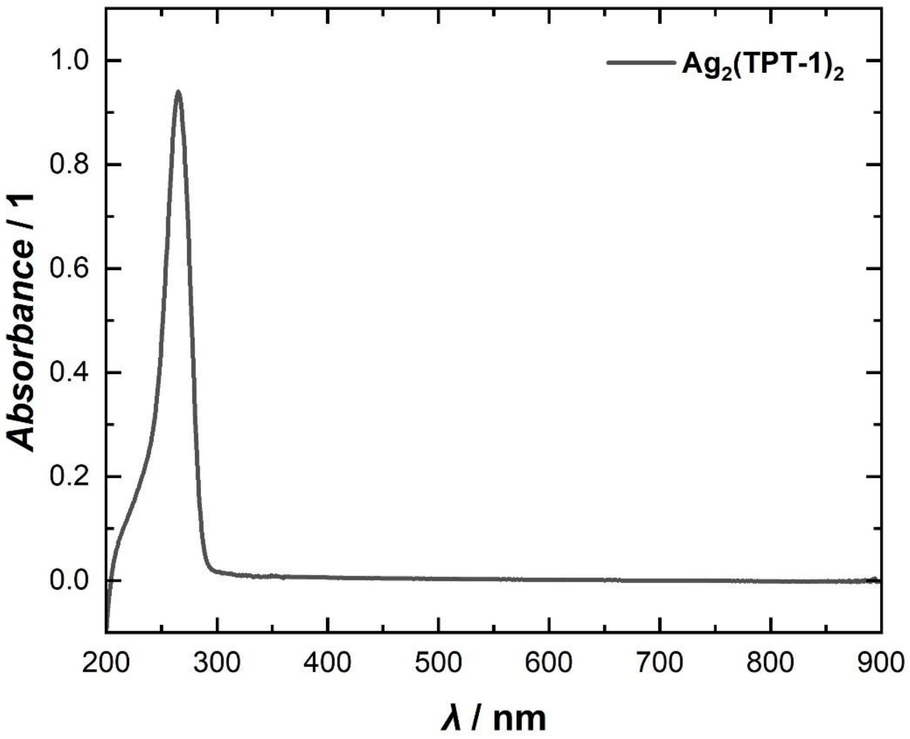

Figure S28. UV/Vis absorption spectrum of complex **Ag_2(TPT-1)$_2$** in CH_3CN with a maximum at 265 nm.

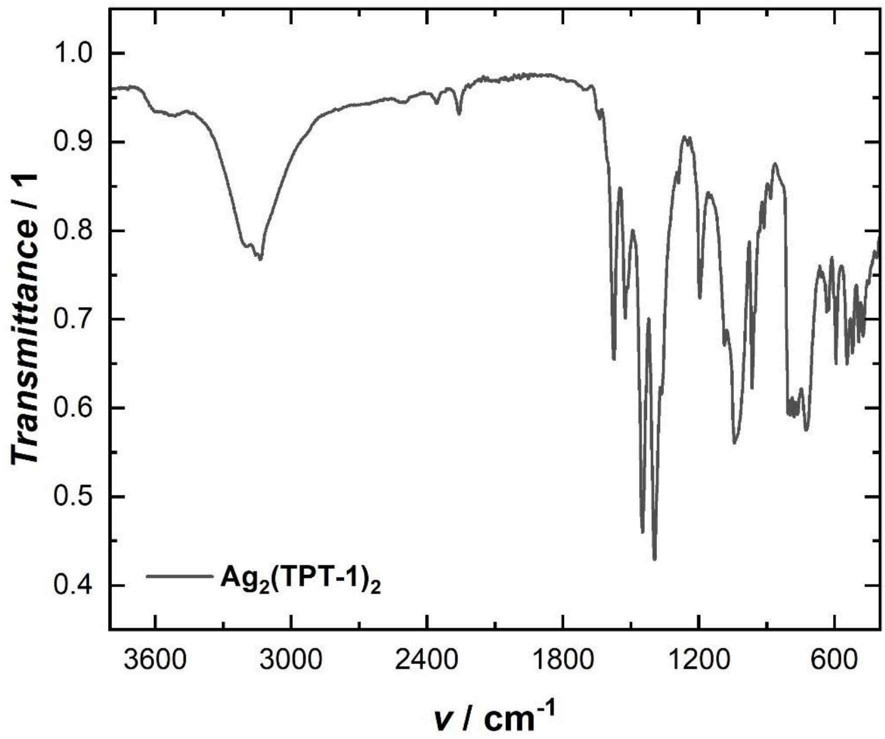

Figure S29. FT-IR absorption spectrum of complex **Ag_2(TPT-1)$_2$** recorded by attenuated total reflection (ATR).

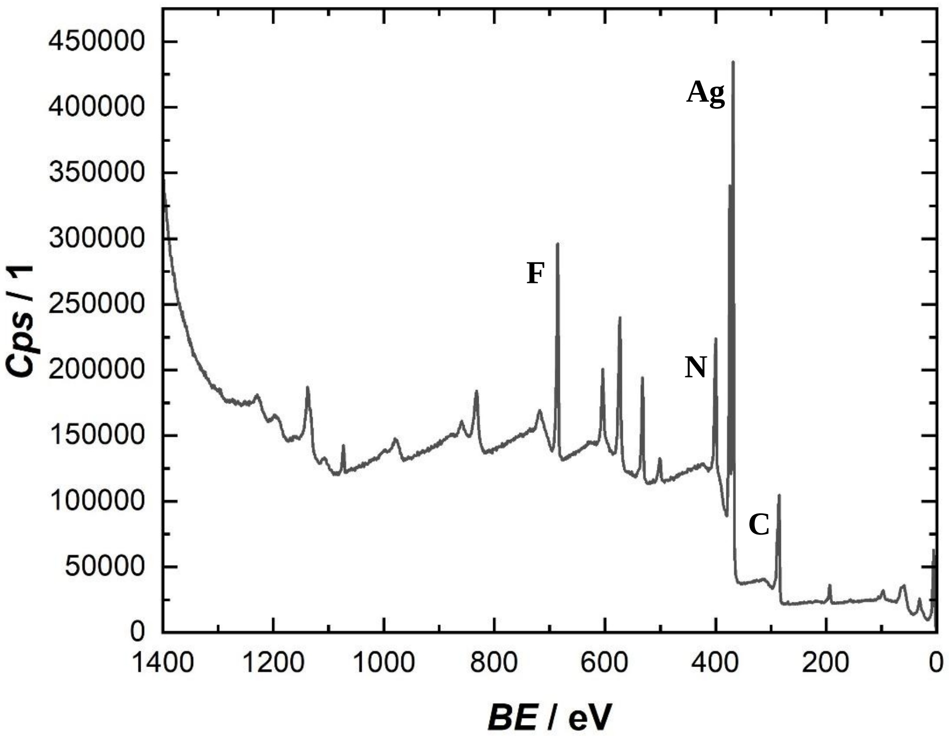

Figure S30. XPS survey spectrum of complex **Ag_2(TPT-1)$_2$** recorded over the range 0–1400 eV. The carbon C 1s peak at 285 eV was used as the reference for binding energy calibration.

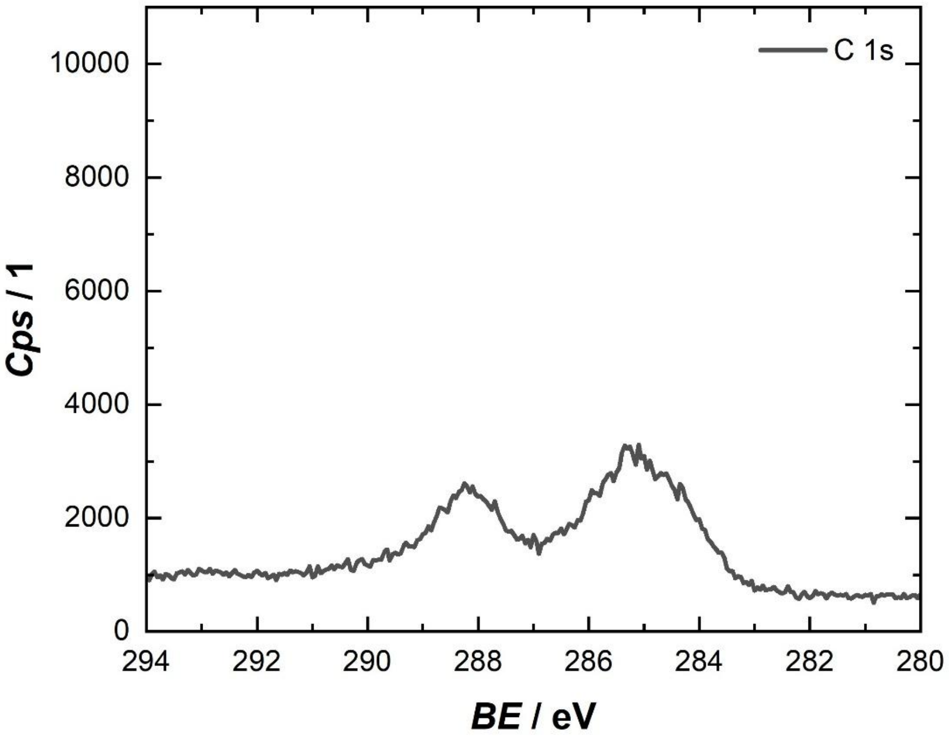

Figure S31. High resolution XPS spectrum of complex **Ag_2(TPT-1)$_2$** for C 1s recorded in the range 280–294 eV.

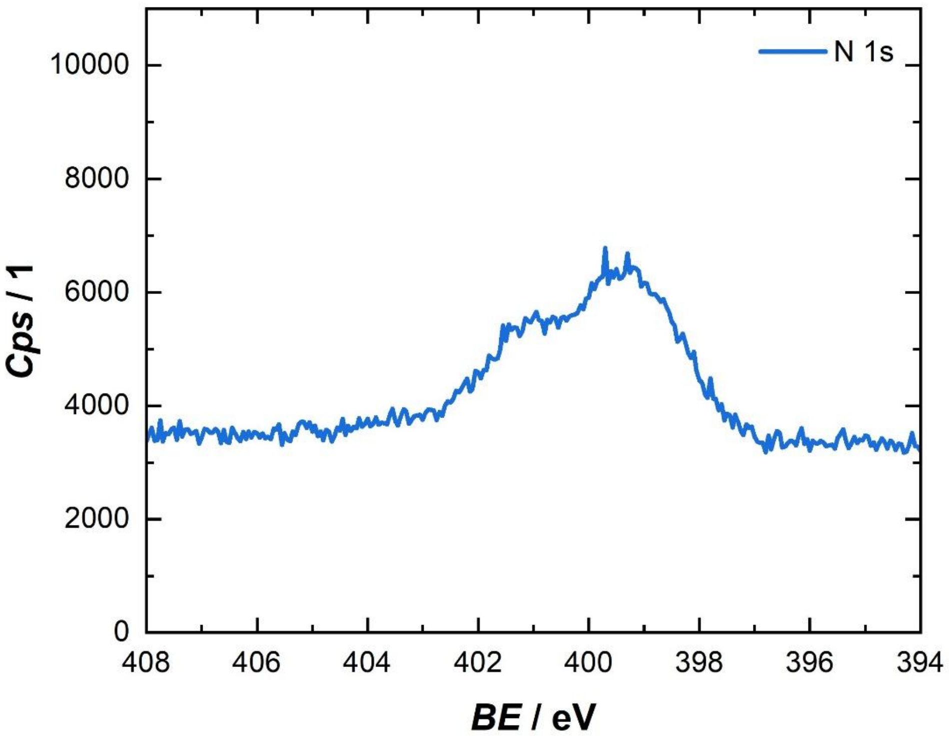

Figure S32. High resolution XPS spectrum of complex **Ag_2(TPT-1)$_2$** for N 1s recorded in the range 394–408 eV.

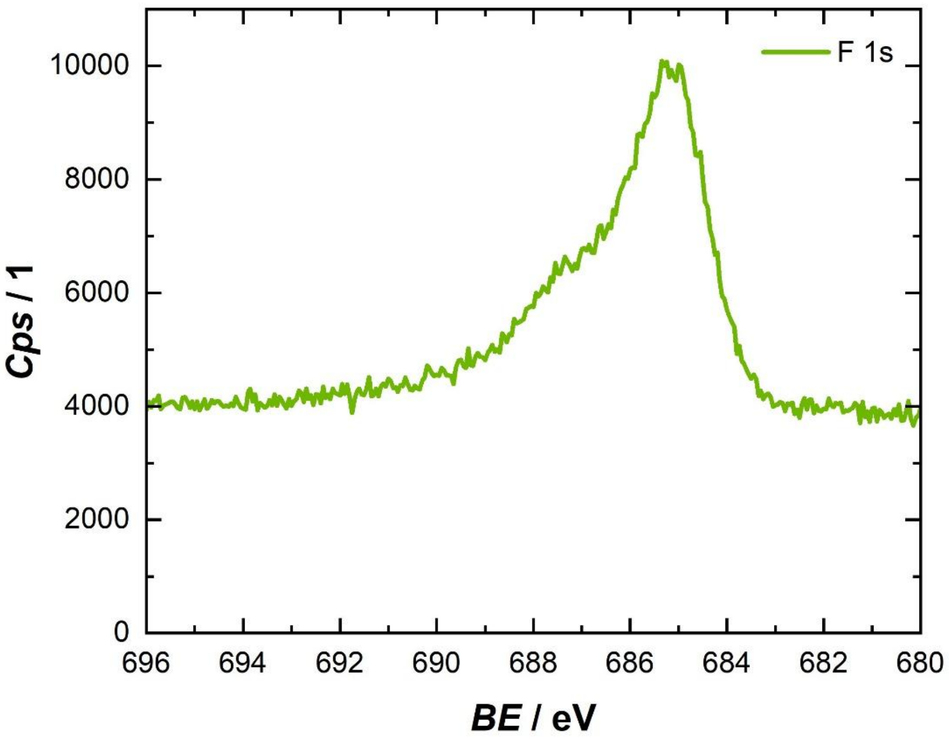

Figure S33. High resolution XPS spectrum of complex **Ag_2(TPT-1)$_2$** for F 1s recorded in the range 680–696 eV.

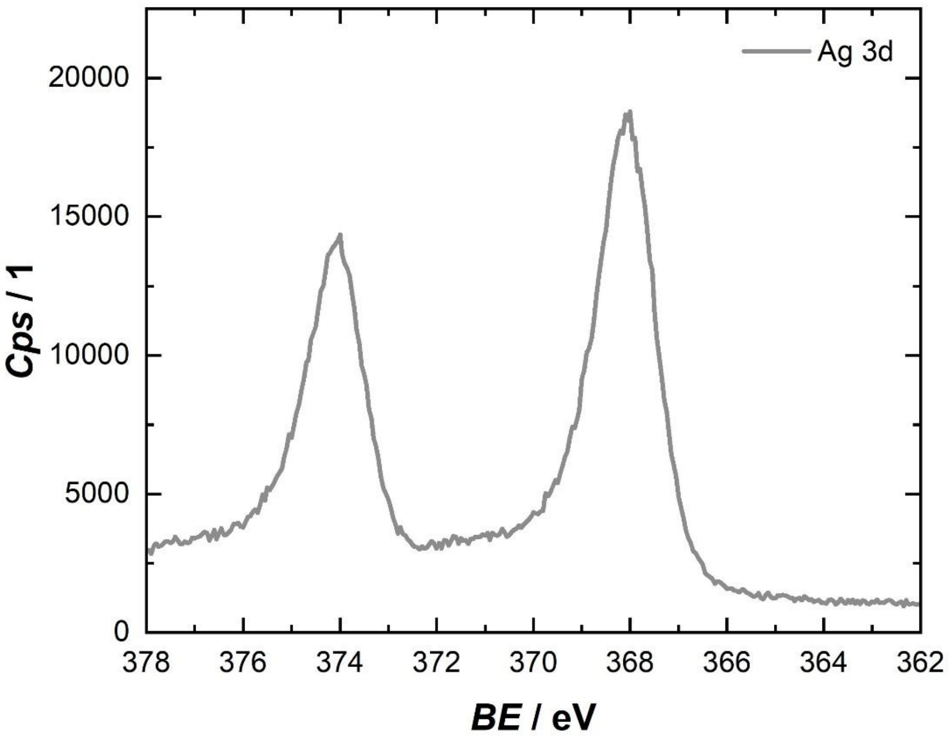

Figure S34. High resolution XPS spectrum of complex **Ag_2(TPT-1)$_2$** for Ag 3d recorded in the range 362–378 eV.

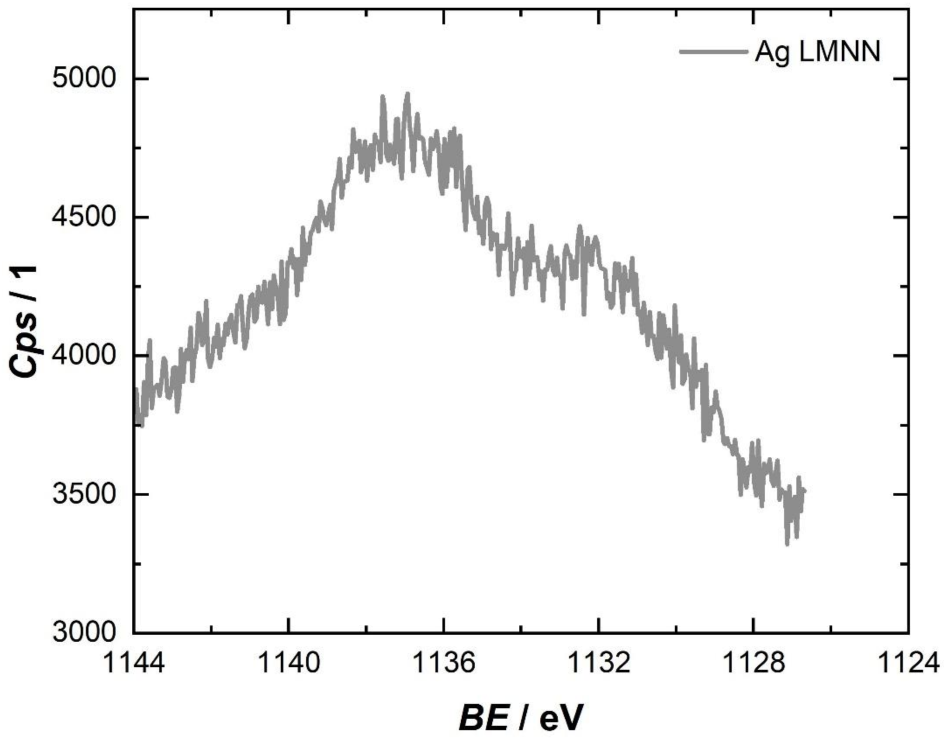

Figure S35. High resolution XPS spectrum of complex **Ag_2(TPT-1)$_2$** for Ag LMNN recorded in the range 1124–1144 eV.

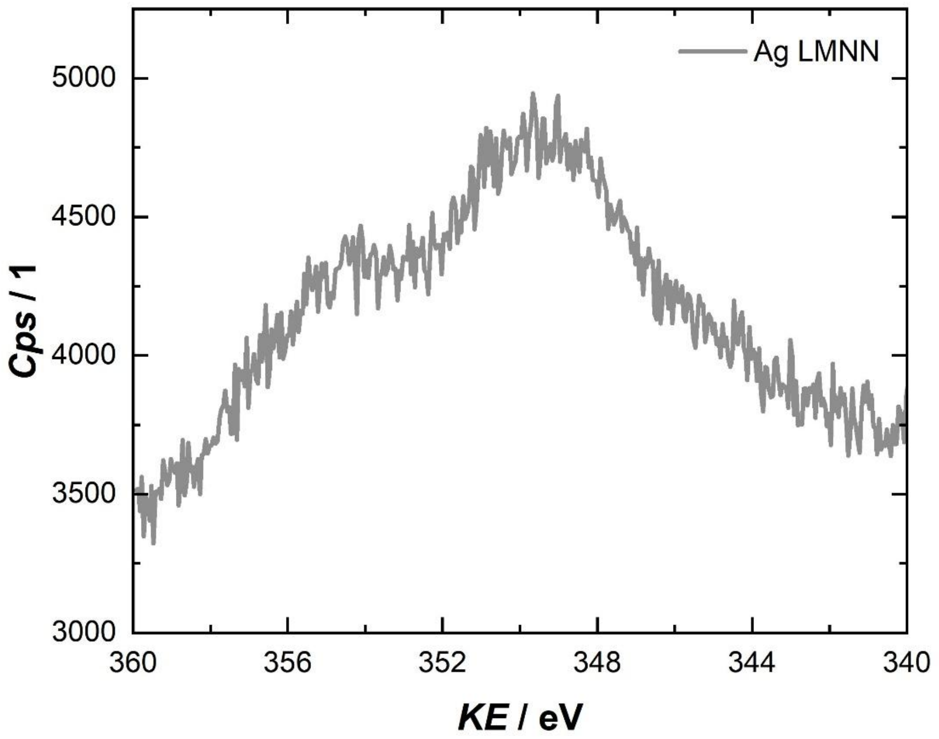

Figure S36. High resolution XPS spectrum of complex **Ag$_2$(TPT-1)$_2$** for Ag LMNN recorded in the range 340–360 eV.

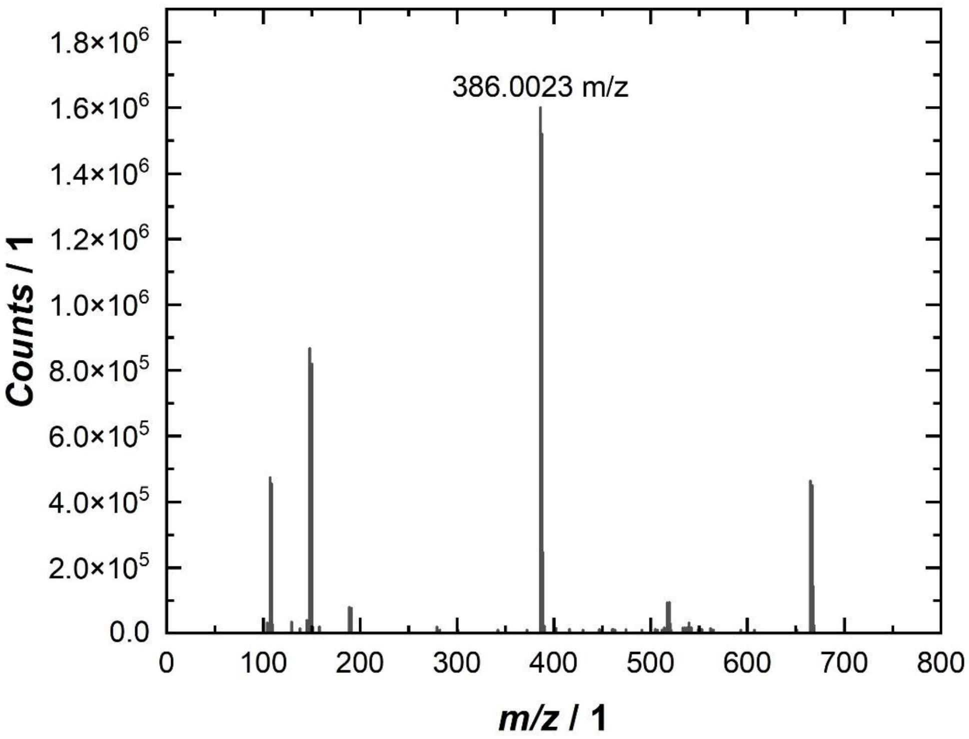

Figure S37. HRMS-ESI spectrum of complex **Ag$_2$(TPT-1)$_2$** with $[M]^{2+}$ at 386.0023 m/z (t_R = 0.361 min).

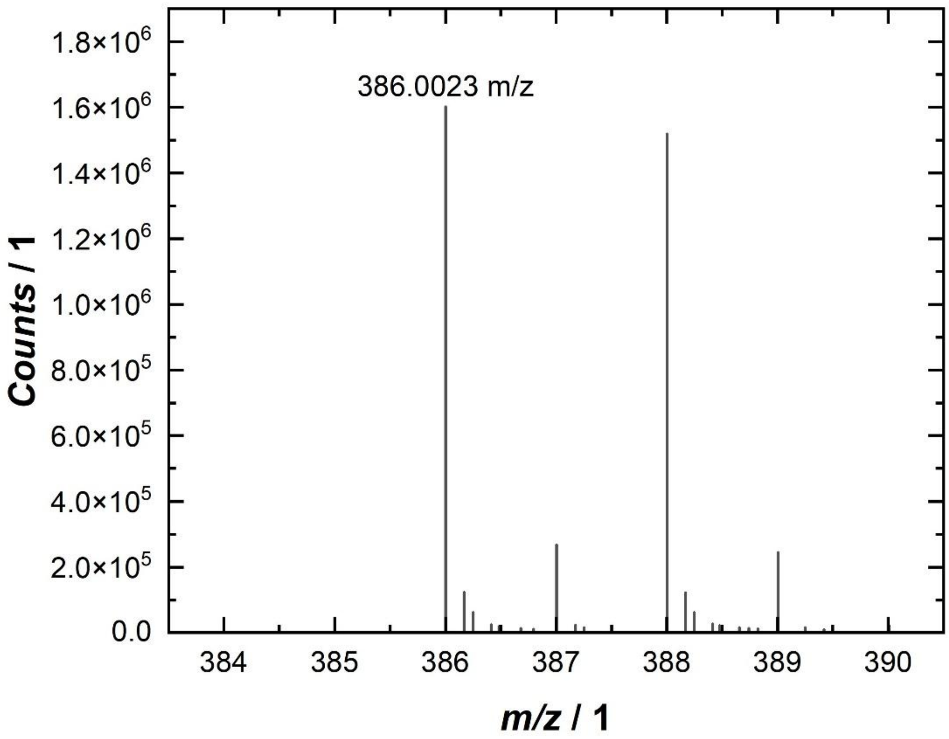

Figure S38. Zoomed-in HRMS-ESI spectrum of complex **Ag_2(TPT-1)$_2$** with $[M]^{2+}$ at 386.0023 m/z (t_R = 0.361 min).

2. Results and Discussion

2.1 Homo- and Heterogeneous Electrochemical Setups

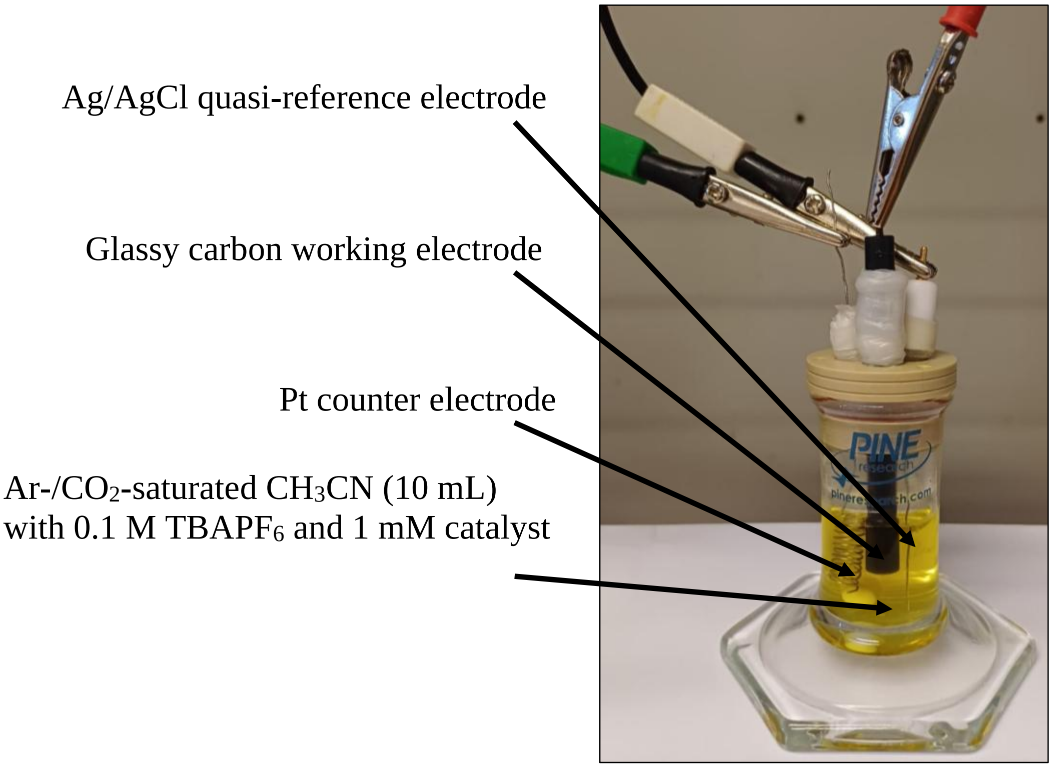

Figure S39. One-compartment cell using a standard three-electrode configuration with glassy carbon working, Pt counter and Ag/AgCl quasi-reference electrode. To minimize the ohmic drop, the three electrodes were placed close together. Cyclic voltammetry was performed in argon- and CO_2-saturated CH_3CN with 0.1 M $TBAPF_6$ as supporting electrolyte and 1 mM catalyst.

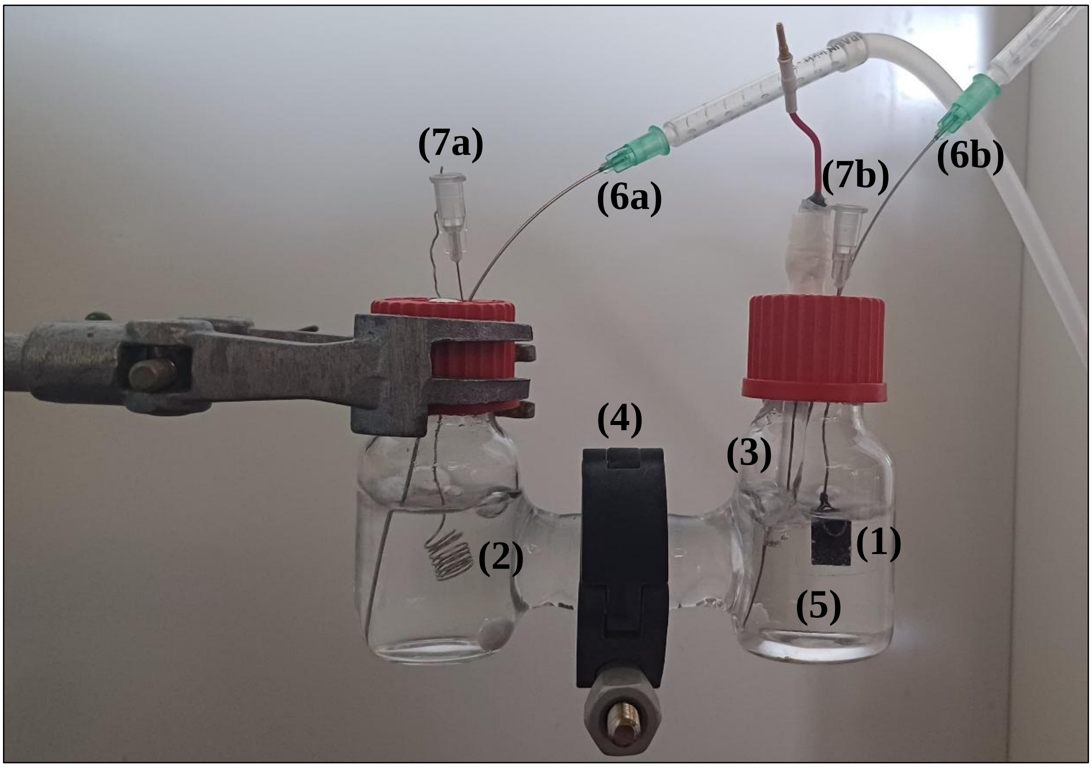

Figure S40. H-type electrochemical cell with **(1)** spray-coated Toray TGP-H-60 carbon paper attached to a Ta-clip (WE), **(2)** Pt wire (CE), **(3)** Ag/AgCl 3 M KCl electrode (RE), **(4)** Nafion-117 perfluorosulfonic acid proton-exchange membrane, **(5)** 0.1 M $CsHCO_3$ electrolyte, **(6a)** and **(6b)** gas inlets to anode (left) and cathode (right) compartments, **(7a)** and **(7b)** gas outlets to anode (left) and cathode (right) compartments.

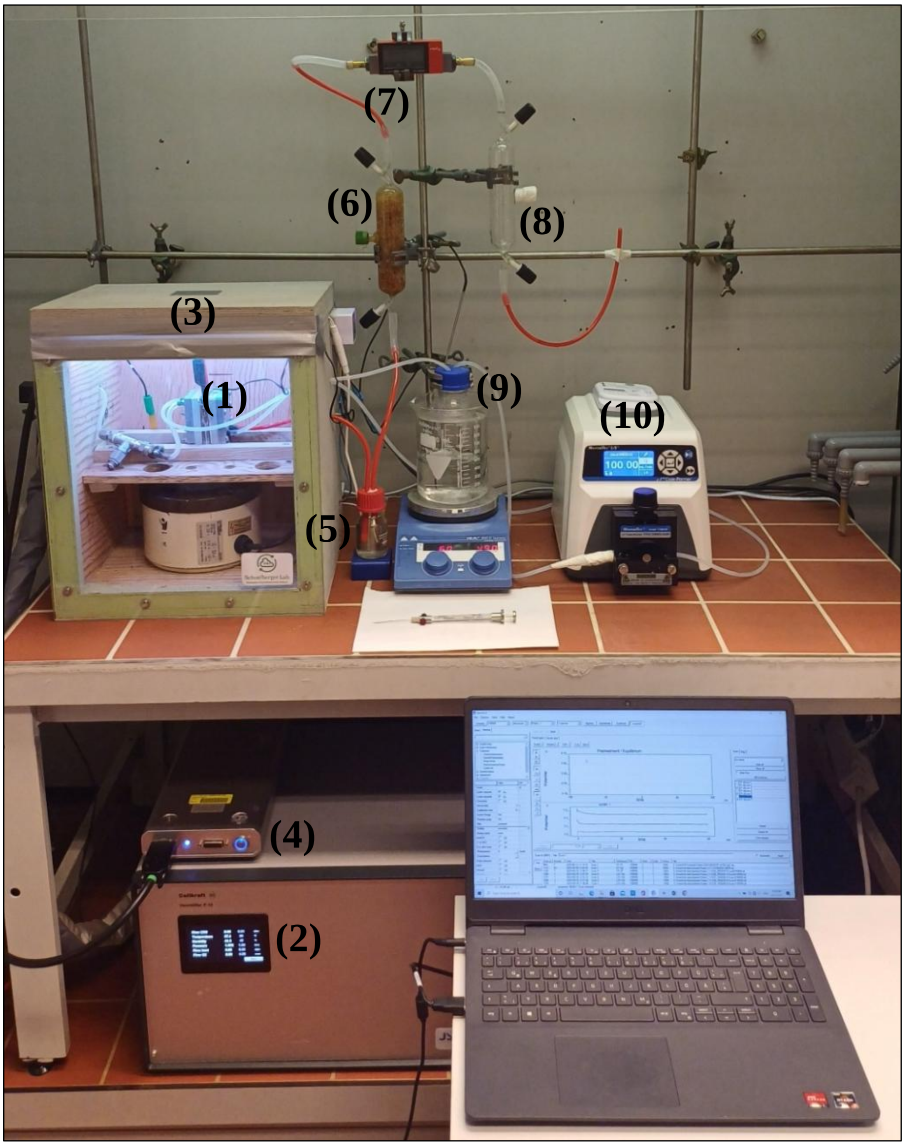

Figure S41. **(1)** Zero-gap electrochemical cell with **(2)** humidifier, **(3)** custom-built oven, **(4)** potentiostat, **(5)** gas bubbler, **(6)** drying tube, **(7)** flow meter, **(8)** gas mouse, **(9)** analyte reservoir, **(10)** analyte pump. The CO_2 gas cylinder connected to the setup is placed outside the image frame.

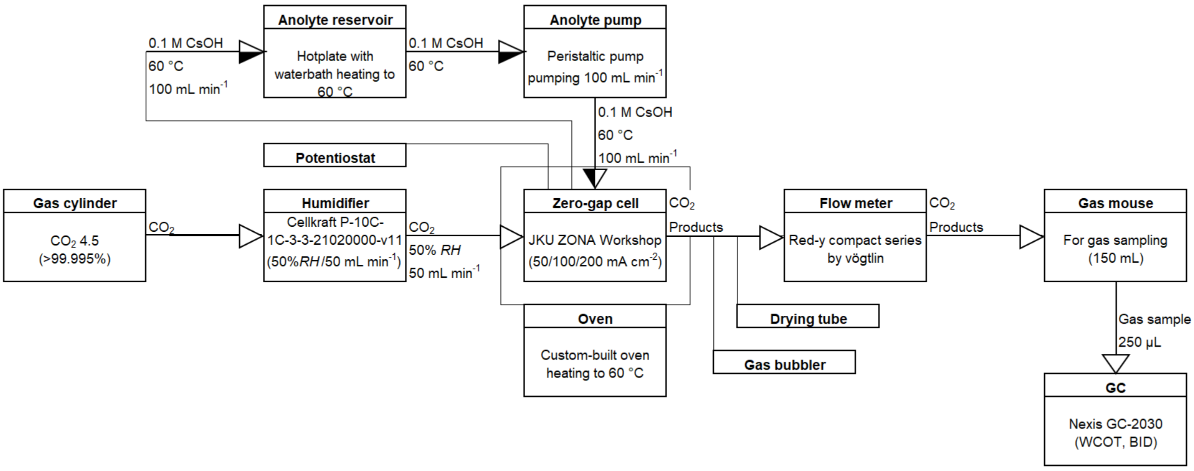

Scheme S4. Flow-scheme of the zero-gap electrochemical cell including gas cylinder, humidifier, oven, potentiostat, gas bubbler, drying tube, flow meter, gas mouse, GC, anolyte reservoir and anolyte pump.

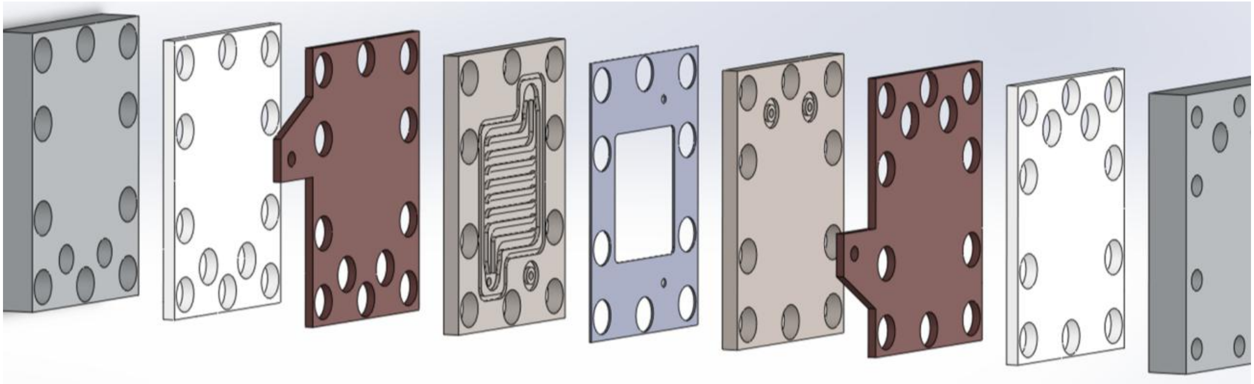

Figure S42. Schematic illustration of the zero-gap electrochemical cell (JKU ZONA Workshop) with aluminum endplate, Teflon endplate, copper plate as current collector, cathodic serpentine flow field for CO_2, Teflon spacer (d = 0.25 mm), anodic parallel flow field for 0.1 M CsOH, copper plate as current collector, Teflon endplate and aluminum endplate (from left to right).

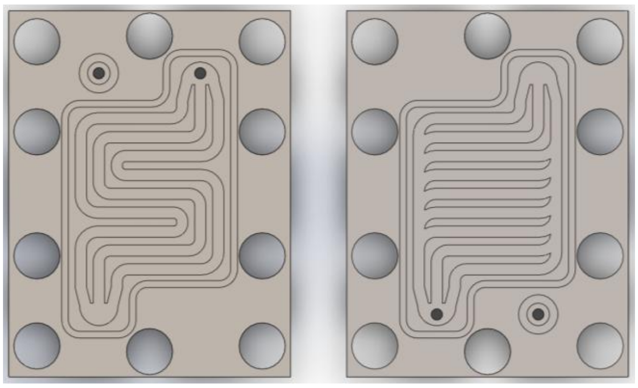

Figure S43. Schematic illustration of the cathodic serpentine flow field for CO_2, featuring a channel width of 2.0 mm and a depth of 0.5 mm (left), and the anodic parallel flow field for 0.1 M CsOH, again featuring a channel width of 2.0 mm but a depth of 1.0 mm (right).

2.2 Homo- and Heterogeneous Electrochemical Results

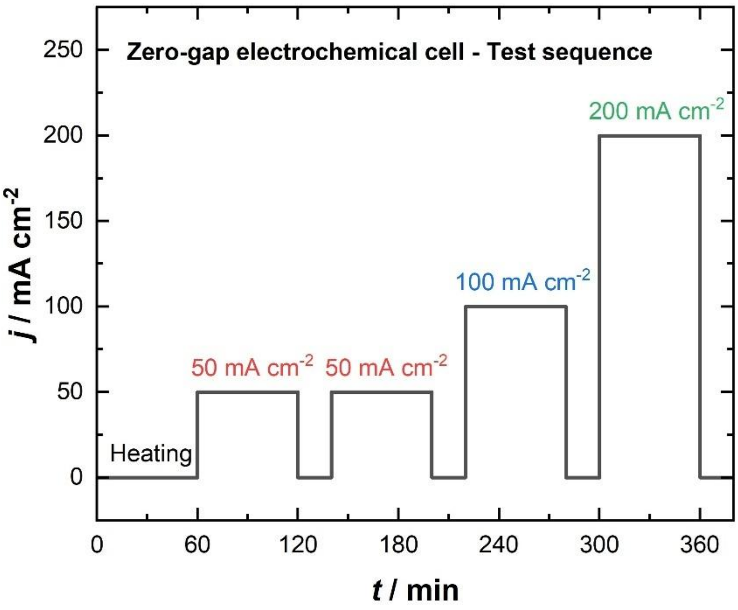

Figure S44. Chronopotentiometric test sequence defined for heterogeneous zero-gap electrochemical CO_2 reduction, consisting of 60 min steps at 50 (induction period), 50, 100 and 200 mA cm^{-2}, separated by 20 min open-circuit intervals. The test sequence was initiated by an initial heating phase for 60 min.

Gaseous products from the headspace of the cathode compartment of the H-type electrochemical cell and from the product gas stream of the zero-gap electrochemical cell (via Gas Mouse) were quantified by gas chromatography. A 250 µL gas sample was withdrawn with a gas-tight syringe (Valco® Precision Sampling Syringe, Series A-2 500 µL, VICI Precision Sampling) and injected in a Shimadzu Nexis GC-2030 system equipped with WCOT columns (Q-BOND, 30 m × 0.53 mm, 10 µm; QS-BOND, 30 m × 0.53 mm, 10 µm) and BID-2030 barrier ionization discharge detectors. The GC was operated with a split ratio of 100:1, a column flow of 3.10 mL min^{-1}, a BID detector temperature of 250 °C and discharge gas flow of 50 mL min^{-1}. Helium 5.0 was used as carrier gas. Hydrogen (t_R = 3.8 min) and carbon monoxide (t_R = 6.5 min) were quantified by external standard calibration of the integrated BID detector peak areas.

Liquid products in the electrolyte of the cathode compartment of the H-type electrochemical cell were quantified by 1H-NMR using a Bruker AVIII 500 MHz spectrometer. A 675 µL aliquot of the catholyte was spiked with 75 µL D2O:DMSO (2000:1), with DMSO serving as the internal standard. Product quantities were calculated from the integrated peak areas, using the known concentration of DMSO corrected by the number of protons contributing to each resonance. For the zero-gap electrochemical cell, no liquid product quantification was performed.

2.3 2,4,6-Tri-(1H-Pyrazol-1-yl)-1,3,5-Triazine

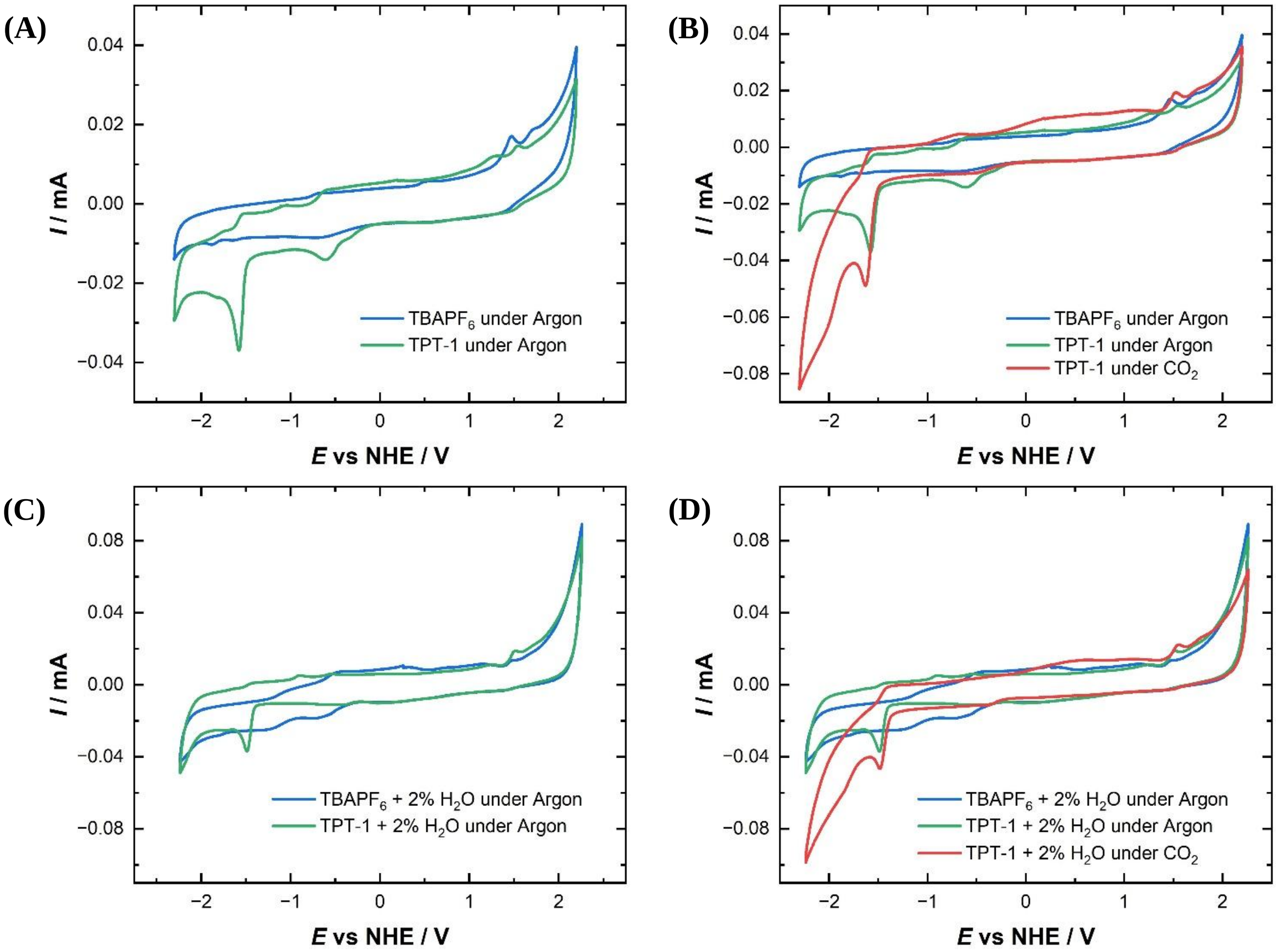

Figure S45. Cyclic voltammograms of ligand **TPT-1** in CH_3CN containing 0.1 M $TBAPF_6$ as supporting electrolyte under **(A)** argon atmosphere, **(B)** CO_2 atmosphere, **(C)** argon atmosphere with 2% water as proton source and **(D)** CO_2 atmosphere with 2% water as proton source.

2.4 Di-2,4,6-Tri-(1H-Pyrazol-1-yl)-1,3,5-Triazine-Silver(I)-Tetrafluoroborate and Di-2,4,6-Tri-(1H-Pyrazol-1-yl)-1,3,5-Triazine-Silver(I)Silver(I)-Tetrafluoroborate

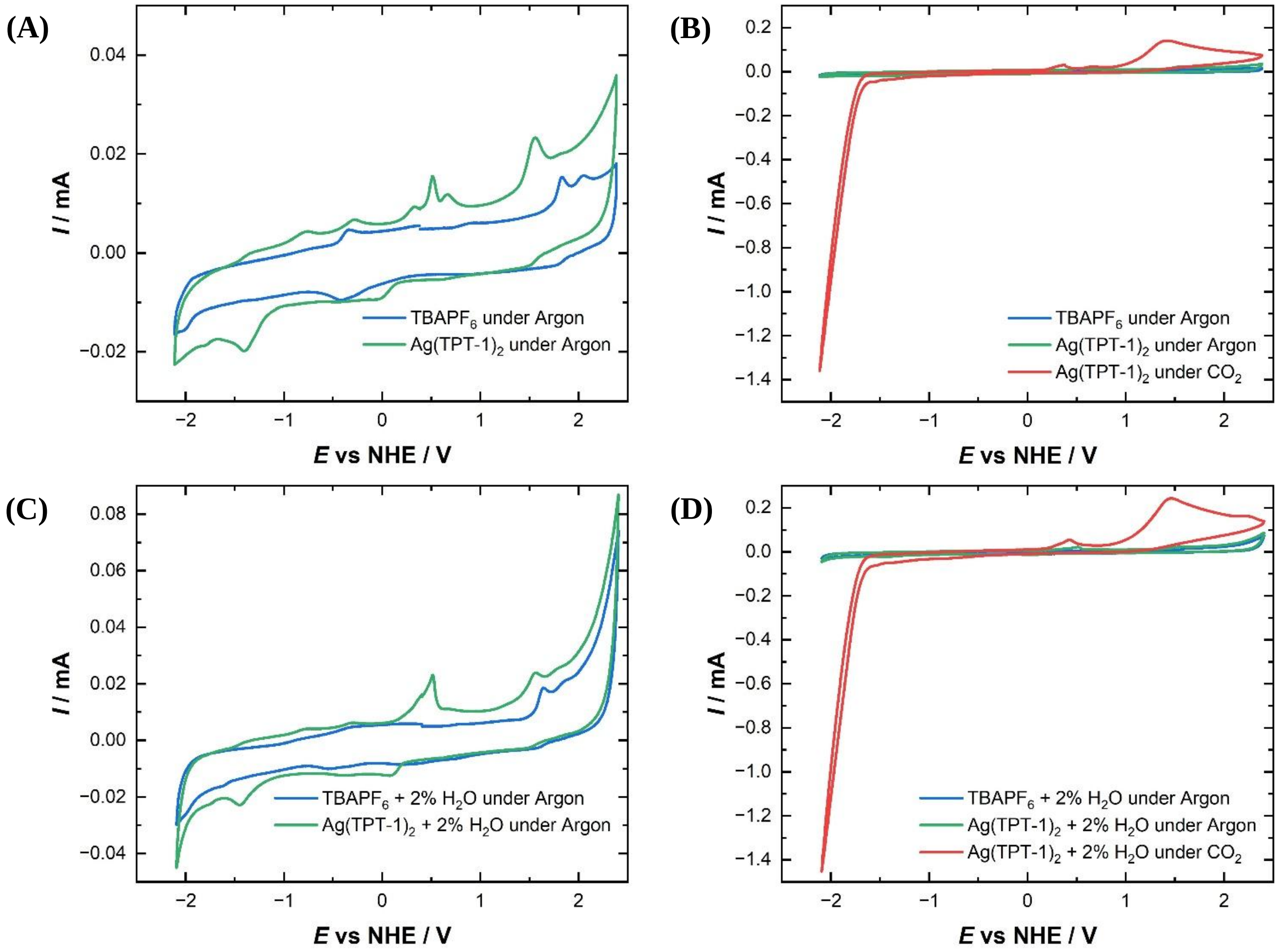

Figure S46. Cyclic voltammograms of complex **Ag(TPT-1)₂** in CH_3CN containing 0.1 M $TBAPF_6$ as supporting electrolyte under **(A)** argon atmosphere, **(B)** CO_2 atmosphere, **(C)** argon atmosphere with 2% water as proton source and **(D)** CO_2 atmosphere with 2% water as proton source.

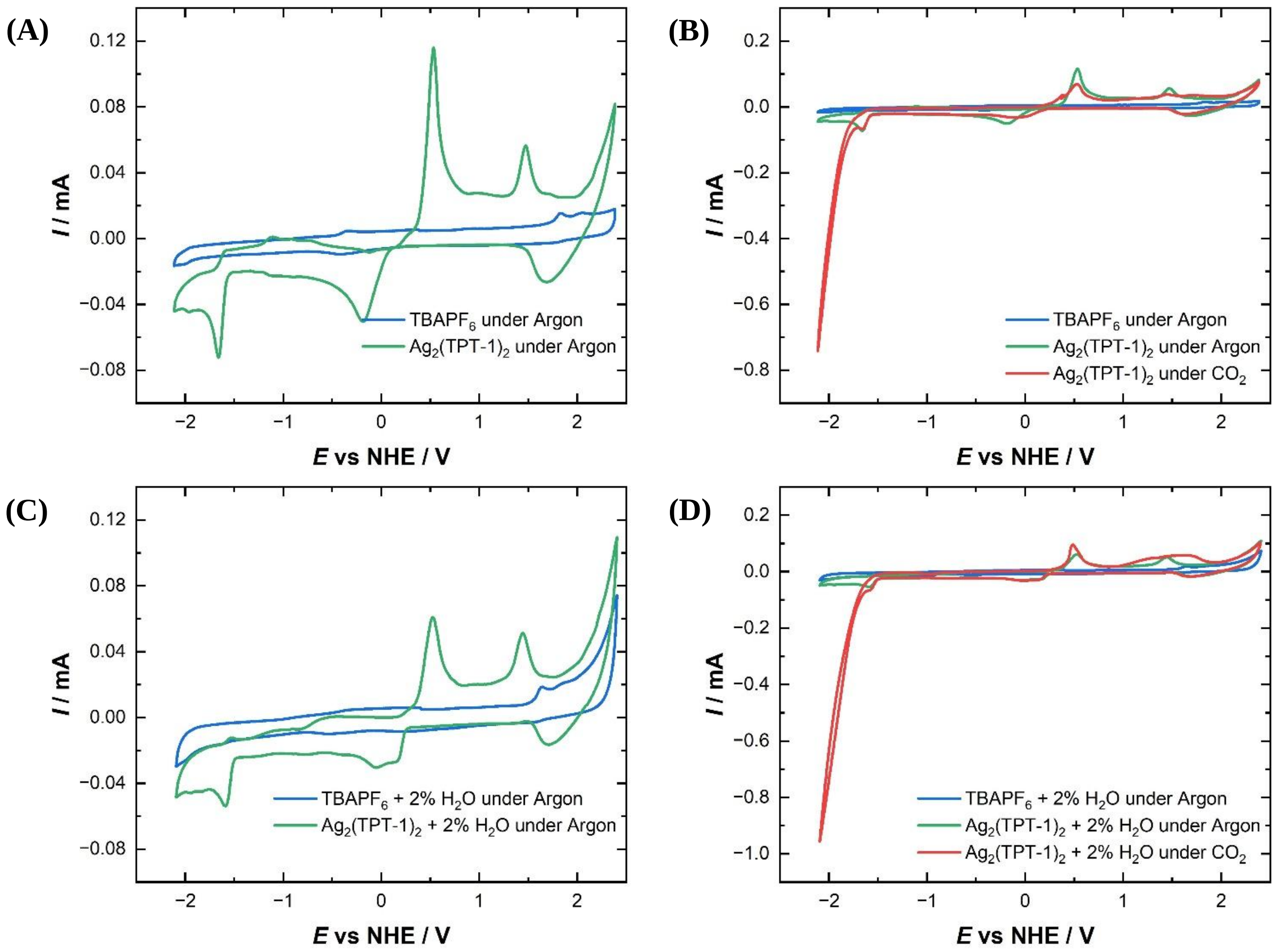

Figure S47. Cyclic voltammograms of complex **Ag$_2$(TPT-1)$_2$** in CH_3CN containing 0.1 M $TBAPF_6$ as supporting electrolyte under **(A)** argon atmosphere, **(B)** CO_2 atmosphere, **(C)** argon atmosphere with 2% water as proton source and **(D)** CO_2 atmosphere with 2% water as proton source.

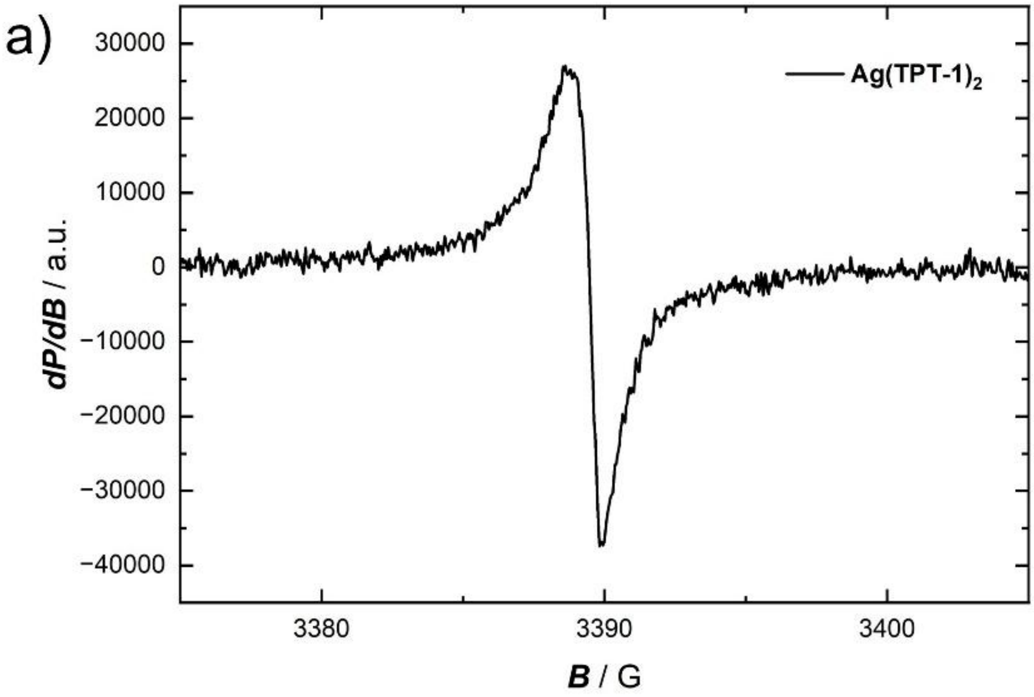

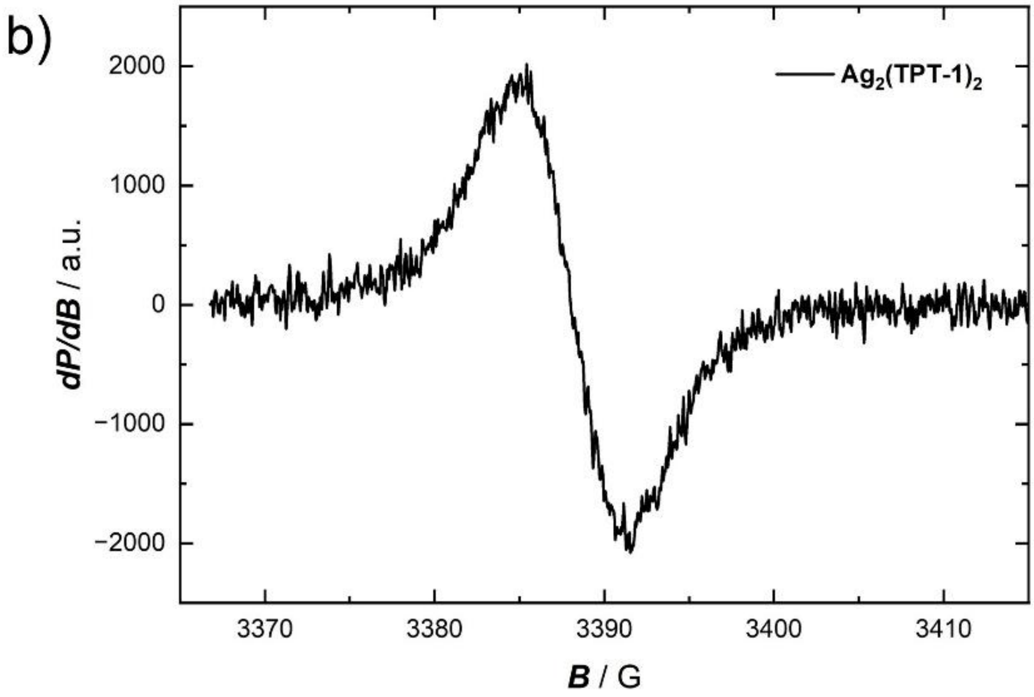

Figure S48. a) and b) EPR spectra of complexes **Ag(TPT-1)$_2$** and **Ag$_2$(TPT-1)$_2$** after reduction with KC_8 in THF at 298 K.

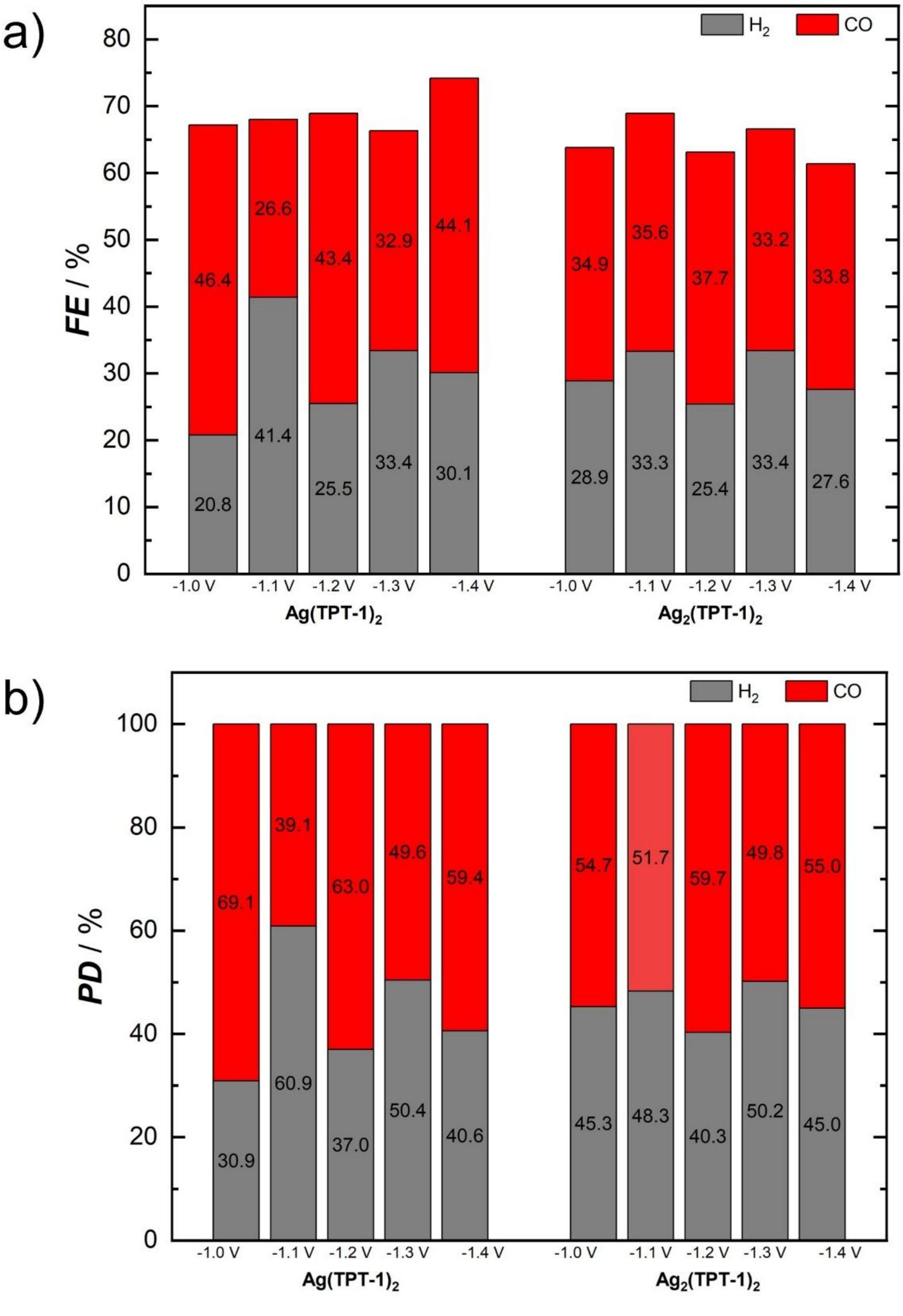

Figure S49. a) Faradaic efficiencies and b) product distributions for H_2 and CO obtained by chronoamperometry with **Ag(TPT-1)$_2$** and **Ag$_2$(TPT-1)$_2$** in an H-type electrochemical cell at -1.0, -1.1, -1.2, -1.3 and -1.4 V vs RHE.

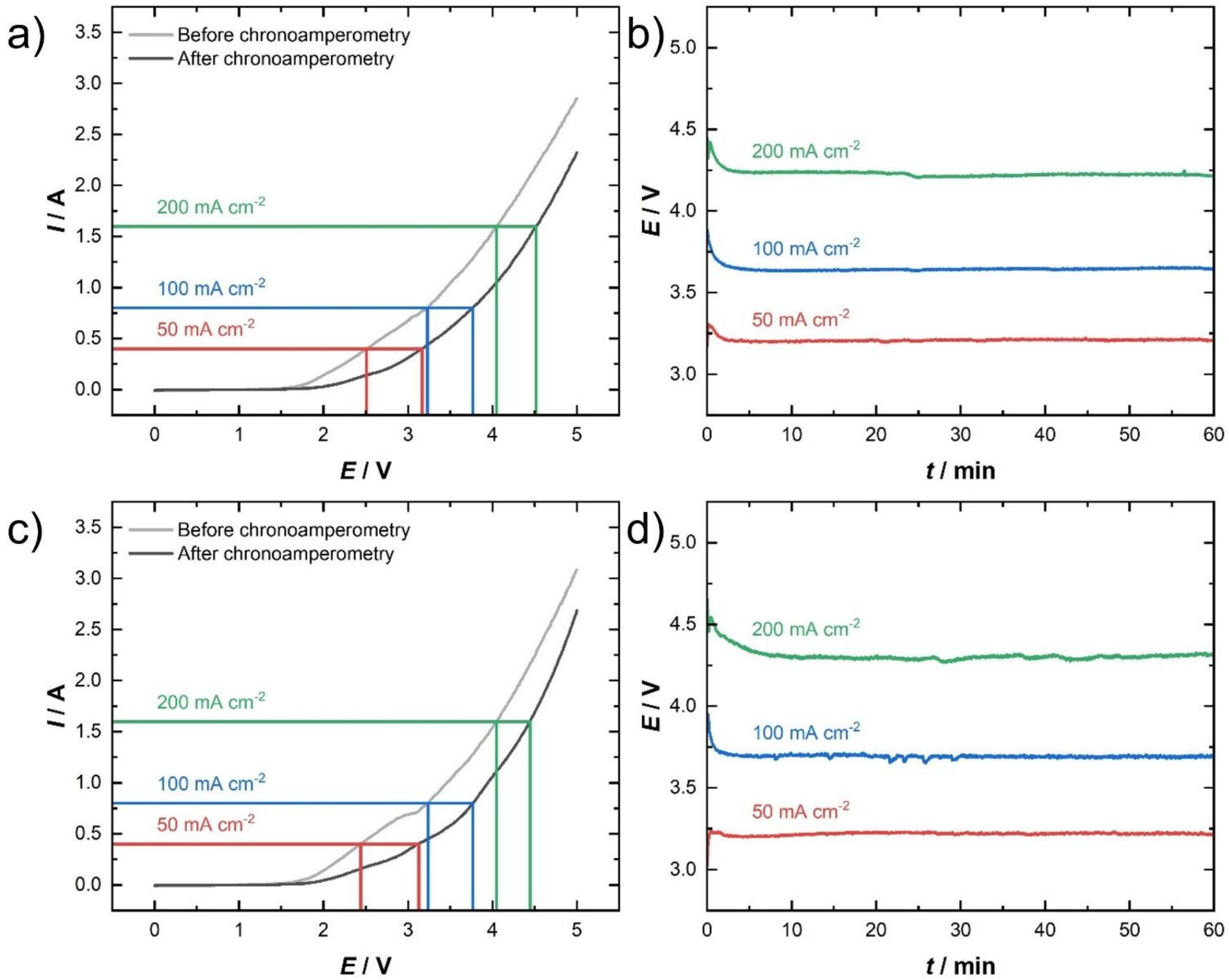

Figure S50. a) and c) Linear sweep voltammograms of complexes **Ag(TPT-1)$_2$** and **Ag$_2$(TPT-1)$_2$** recorded before and after chronopotentiometry, indicating the effect of extended electrolysis on the catalytic response. b and d) Chronopotentiometric potential-time curves (*E*-*t*) at constant current densities of 50, 100 and 200 mA cm^{-2}, showing the required operating potentials under different current loads.

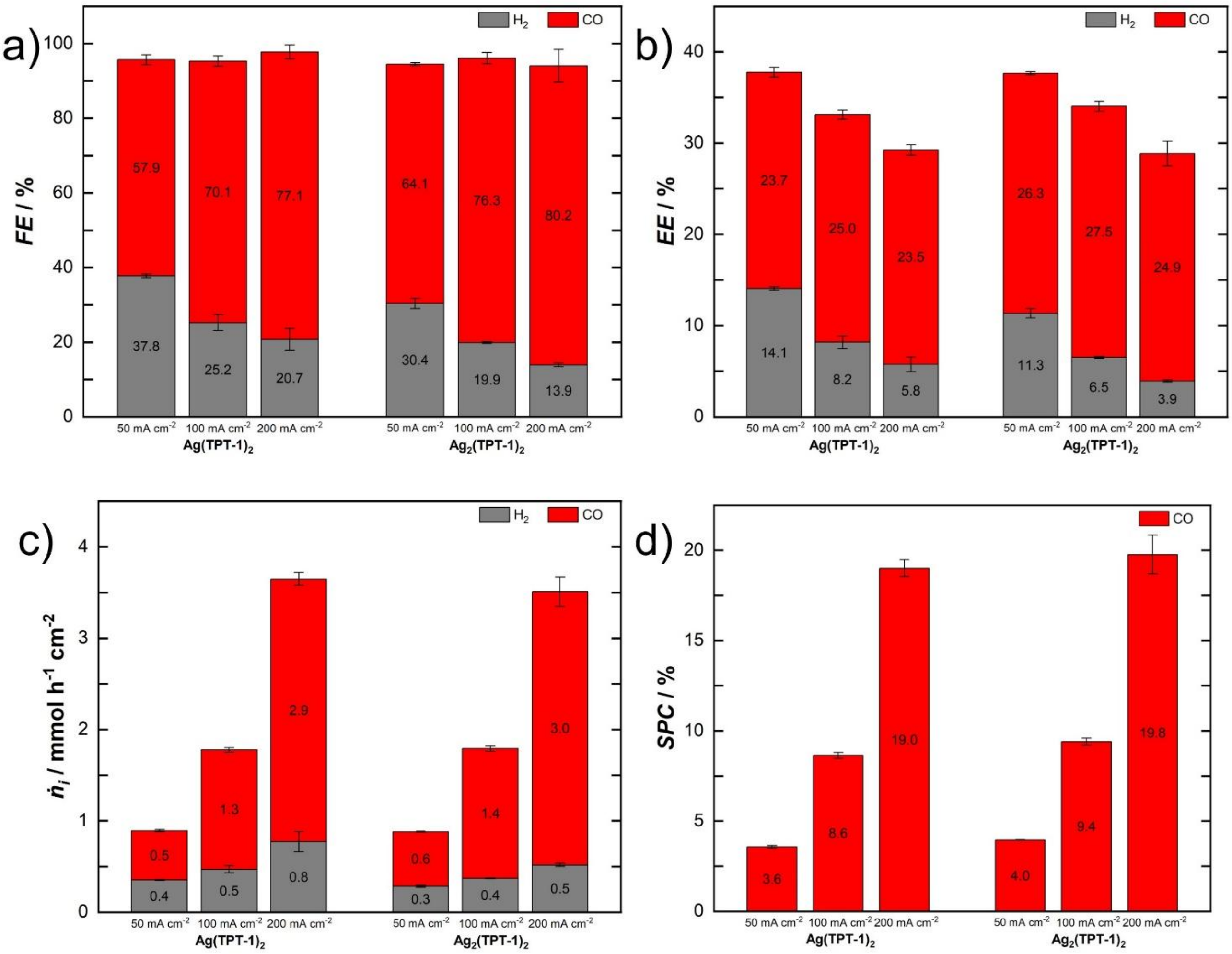

Figure S51. Faradaic efficiencies for H_2 and CO, demonstrating selective CO formation at higher current densities. b) Energy efficiencies for H_2 and CO, revealing the trade-off between product selectivity and energy input. c) Molar production rates of H_2 and CO normalized to the geometrical electrode area (8 cm^2), highlighting markedly increased CO formation at 200 mA cm^{-2}. d) Single-pass conversion for CO, reflecting increased CO_2 utilization at 200 mA cm^{-2}. Measurements were conducted in a custom-built CO_2 electrolyzer cell (JKU ZONA-Workshop) operated at 60 °C, with a CO_2 inlet stream of 50 mL min^{-1} at 50% *RH* and an anolyte flow rate of 100 mL min^{-1} 0.1 M CsOH. Taken together, graphs a-d) demonstrate the CO selectivity and effective CO_2 conversion of complexes **Ag(TPT-1)₂** and **Ag₂(TPT-1)₂** at elevated current densities, emphasizing their potential as efficient catalysts for electrochemical CO_2 reduction.

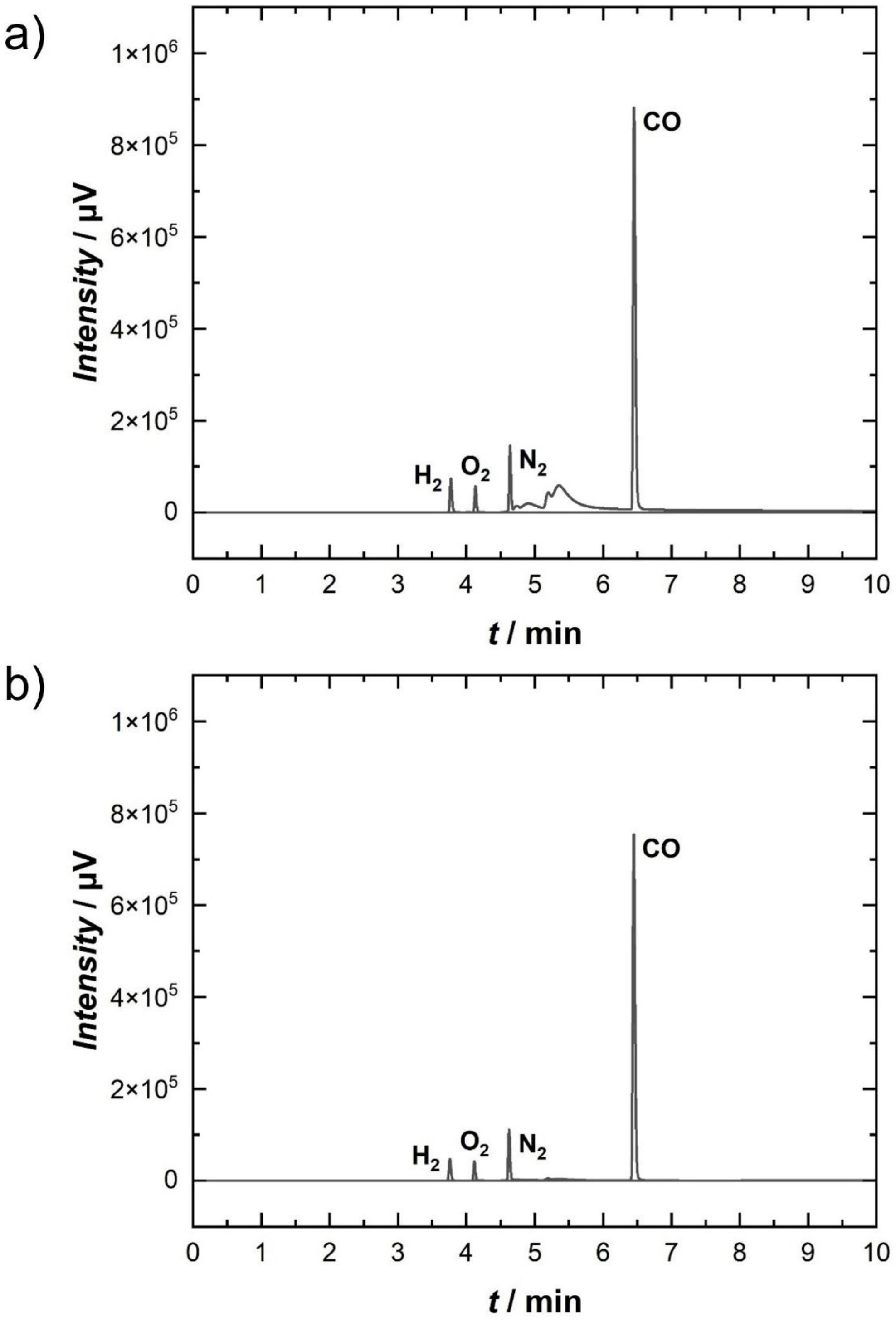

Figure S52. GC spectra of a) complex **Ag(TPT-1)$_2$** and b) complex **Ag$_2$(TPT-1)$_2$** for chronopotentiometry at 200 mA cm^{-2}.

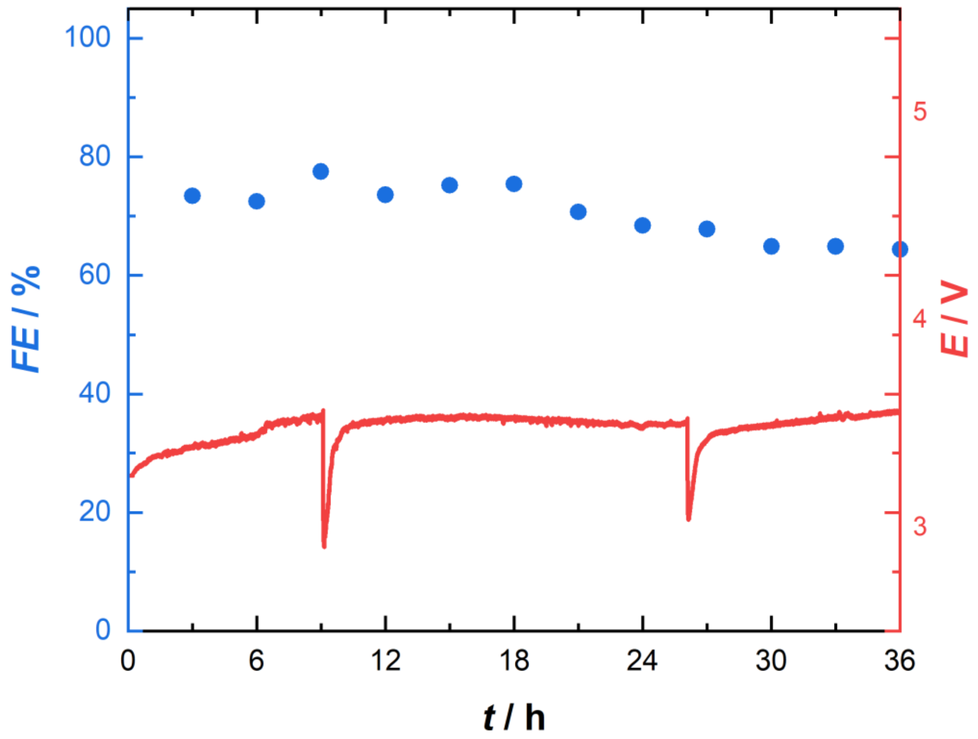

Figure S53. CO_2R performance of 1.0 mg cm^{-2} **Ag(TPT-1)$_2$** on a CT Carbon Cloth with MPL - W1S1011 in a zero-gap cell at 60 °C utilizing a serpentine flow field design. Faradaic efficiencies over a period of 30 h of galvanostatic electrolysis at a current density of 200 mA cm^{-2} in 0.1M CsOH and corresponding cell potential curve. The red spikes result from changing the analyte.

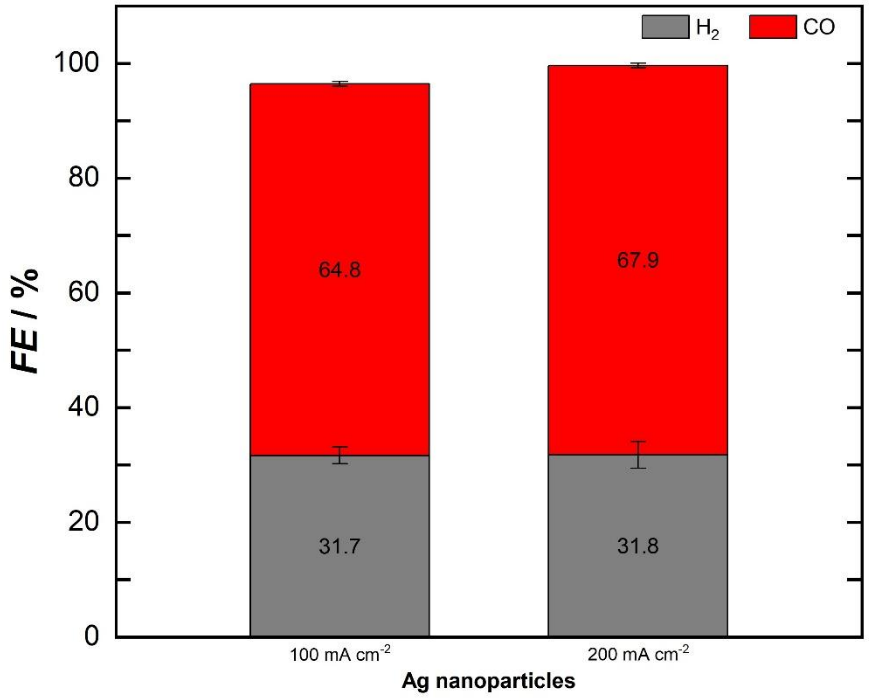

Figure S54. Faradaic efficiencies for H_2 and CO obtained for **AgNPs** at constant current densities of 100 and 200 mA cm^{-2}.

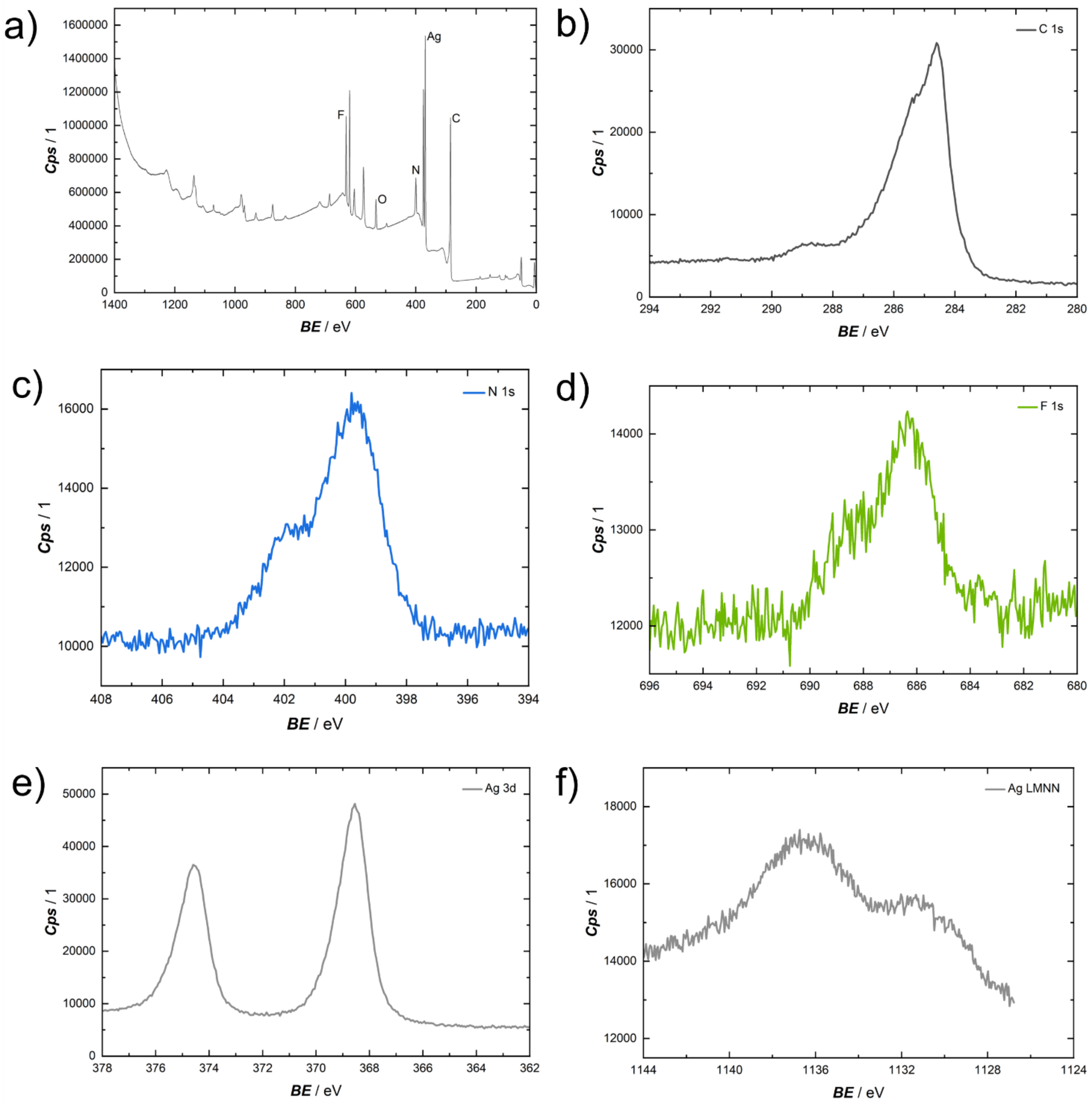

Figure S55. XPS analysis of 1.0 mg cm^{-2} **Ag(TPT-1)$_2$** on a CT Carbon Cloth with MPL - W1S1011 before zero-gap cell measurements.

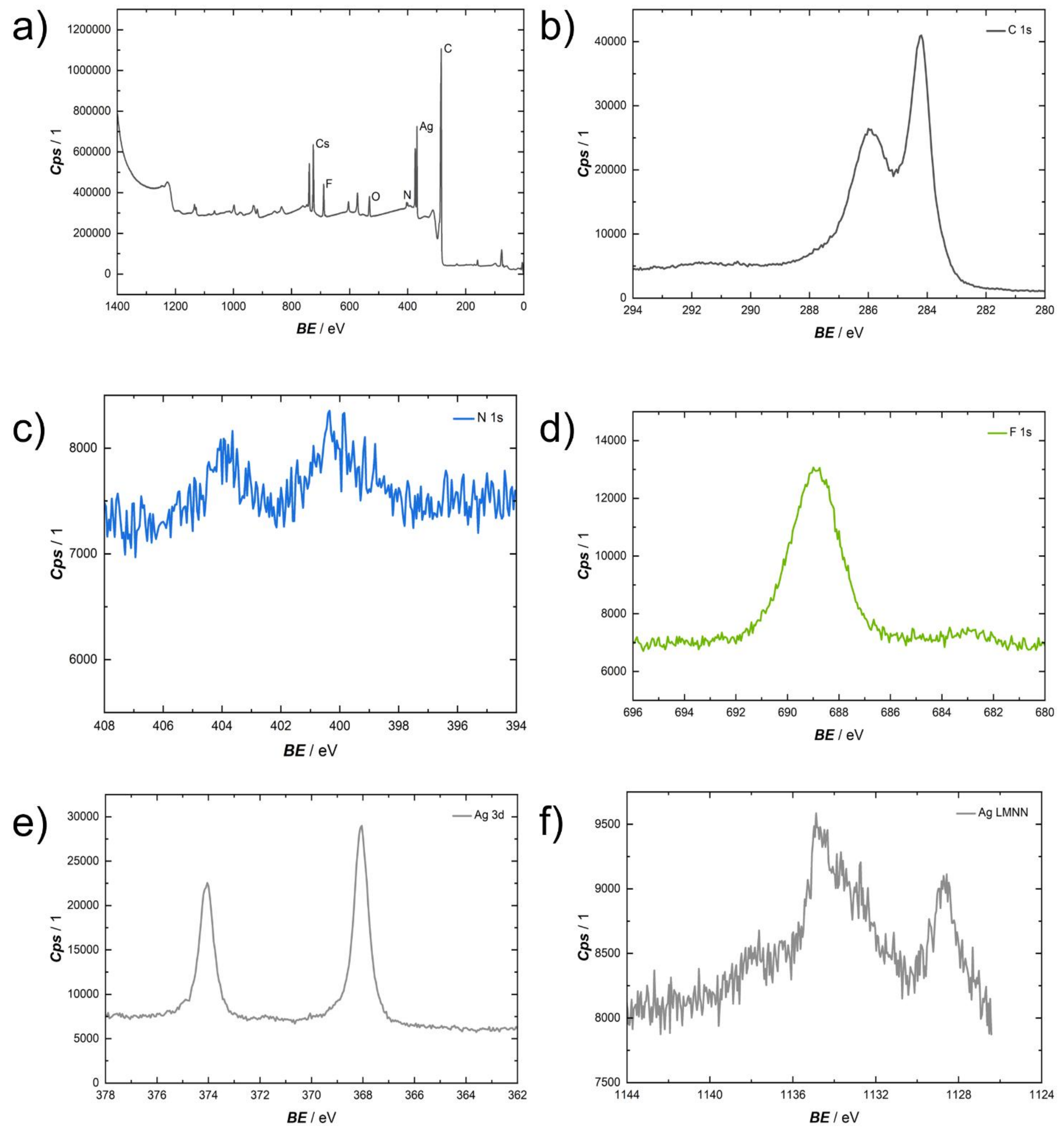

Figure S56. XPS post analysis of 1.0 mg cm^{-2} **Ag(TPT-1)$_2$** on a CT Carbon Cloth with MPL - W1S1011 after zero-gap cell measurements.

Table S1. Comparison of CO_2RR Catalysts at High Current Densities (CO Production)

Catalyst System	Cell Type	Current Density (mA cm^{-2})	CO Faradaic Efficiency (%)	Cell Voltage / Potential	Stability	Key Features / Notes	Ref.
Ag@C-d (defect-confined Ag)	Flow cell / MEA (acidic)	200	91.6	~3.15 V (cell)	>100 h	Near-unity FE across 50–500 mA cm^{-2}; industrially relevant operation	1
Ag@C-d (same system)	Flow cell (acidic)	500	~98	—	>100 h	Maintains high FE even at ultra-high current densities	1
Ag-decorated S–C_3N_4/CNT	Flow cell	>200	>80	−0.77 V vs RHE	—	Hybrid catalyst enabling high-rate CO_2RR in flow configuration	2
Ag nanoparticles (hydrophobic ligand modified)	Zero-gap electrolyzer	~100 (partial CO current)	High (CO dominant)	−1.0 V vs RHE	~4 h	Hydrophobicity improves CO selectivity at high current density	3

Defect-rich Ag nanosheets (GDE)	Flow-through GDE	>100	>90	−0.6 to −1.1 V vs RHE	—	Enhanced CO_2 availability via gas-fed architecture	[4]
Infinite Ag cluster catalyst	Flow cell (GDE)	100–350	91–99	−1.9 V vs RHE @ 350 mA cm^{-2}	12 h	Molecular/cluster catalyst bridging homogeneous–heterogeneous regimes	[5]
Ag nanoparticle GDE (PV-coupled system)	Flow electrolyzer	7–37	~90–94	—	multi-day	Stable operation under dynamic current conditions	[6]
This work (Ag(I)-TPT gel)	Zero-gap electrolyzer	200	~80-90	3.0-3.5 V	1 h	Molecular gel catalyst; ligand-centered mechanism; high-rate operation	This work

References

(1) Biao Zhang; Jinhan Zou; Zhouhui Chen; Wei Yan; Weidong Liu; Chengyuan Dong; Di Cai; Qinghong Zhang; Ye Wang; Shunji Xie. Defect-engineered carbon-confined silver for enhanced CO2 electrocatalytic reduction to CO in acidic media. *Next Nanotechnology* **2023**, *2*, 100014. DOI: 10.1016/j.nxnano.2023.100014.

(2) Chen, J.; Wang, Z.; Lee, H.; Mao, J.; Grimes, C. A.; Liu, C.; Zhang, M.; Lu, Z.; Chen, Y.; Feng, S.-P. Efficient electroreduction of CO2 to CO by Ag-decorated S-doped g-C3N4/CNT nanocomposites at industrial scale current density. *Materials Today Physics* **2020**, *12*, 100176. DOI: 10.1016/j.mtphys.2019.100176.

(3) Ko, Y.-J.; Lim, C.; Jin, J.; Kim, M. G.; Lee, J. Y.; Seong, T.-Y.; Lee, K.-Y.; Min, B. K.; Choi, J.-Y.; Noh, T.; Hwang, G. W.; Lee, W. H.; Oh, H.-S. Extrinsic hydrophobicity-controlled silver nanoparticles as efficient and stable catalysts for CO2 electrolysis. *Nature communications* **2024**, *15* (1), 3356. DOI: 10.1038/s41467-024-47490-3. Published Online: Apr. 18, 2024.

(4) Chen, G.; Hong, M.; Ma, B.; Kuang, Y.; Rabiee, H.; Xu, X.; Dorosti, F.; Yan, P.; Shah, N.; Nanjundan, A. K.; Zhu, Z.; Wang, H.; Ge, L. Which dominates CO2 to CO electroreduction from low to industrial current density: Catalyst activity or CO2 availability? *Applied Catalysis B: Environment and Energy* **2026**, *381*, 125902. DOI: 10.1016/j.apcatb.2025.125902.

(5) Curet, L.; Lafargue dit-Hauret, W.; Benet-Buchholz, J.; Martínez-Belmonte, M.; Foix, D.; Palomares, E.; Billon, L.; Begué, D.; Viterisi, A. Self-assembled infinite silver cluster with atomic precision as a scalable catalyst for CO 2 -electroreduction under industry-relevant reaction rates. *EES Catal.* **2025**, *3* (2), 286–296. DOI: 10.1039/D4EY00160E.

(6) Cibaka, T.; Merdzhanova, T.; Astakhov, O.; Shcherbachenko, S.; Liu, G.; van Pham, C.; Rau, U.; Strasser, P. Persistent CO2 Reduction Performance of an Ag Nanoparticle Gas Diffusion Electrode in Realistic Dynamic PV-Driven Operation. *Energy & fuels : an American Chemical Society journal* **2026**, *40* (1), 811–818. DOI: 10.1021/acs.energyfuels.5c03523. Published Online: Sep. 24, 2025.
